# The Evolutionary History of Common Genetic Variants Influencing Human Cortical Surface Area

**DOI:** 10.1093/cercor/bhaa327

**Published:** 2020-12-09

**Authors:** Amanda K Tilot, Ekaterina A Khramtsova, Dan Liang, Katrina L Grasby, Neda Jahanshad, Jodie Painter, Lucía Colodro-Conde, Janita Bralten, Derrek P Hibar, Penelope A Lind, Siyao Liu, Sarah M Brotman, Paul M Thompson, Sarah E Medland, Fabio Macciardi, Barbara E Stranger, Lea K Davis, Simon E Fisher, Jason L Stein

**Affiliations:** Language and Genetics Department, Max Planck Institute for Psycholinguistics, Nijmegen, 6500 AH, Netherlands; Mark and Mary Stevens Neuroimaging and Informatics Institute, Keck School of Medicine, University of Southern California, Marina del Rey, CA 90292, USA; Department of Medicine, Section of Genetic Medicine & Institute for Genomics and Systems Biology, University of Chicago, Chicago, IL 60637, USA; Computational Sciences, Janssen Pharmaceuticals, Spring House, PA 19477, USA; Department of Genetics, University of North Carolina, Chapel Hill, NC 27599, USA; UNC Neuroscience Center, University of North Carolina, Chapel Hill, NC 27599, USA; Psychiatric Genetics, QIMR Berghofer Medical Research Institute, Brisbane, QLD 4006, Australia; Mark and Mary Stevens Neuroimaging and Informatics Institute, Keck School of Medicine, University of Southern California, Marina del Rey, CA 90292, USA; Psychiatric Genetics, QIMR Berghofer Medical Research Institute, Brisbane, QLD 4006, Australia; Psychiatric Genetics, QIMR Berghofer Medical Research Institute, Brisbane, QLD 4006, Australia; Radboud University Medical Center, 6525 XZ Nijmegen, Netherlands; Genentech, Inc., South San Francisco, CA 94080, USA; Psychiatric Genetics, QIMR Berghofer Medical Research Institute, Brisbane, QLD 4006, Australia; Department of Genetics, University of North Carolina, Chapel Hill, NC 27599, USA; UNC Neuroscience Center, University of North Carolina, Chapel Hill, NC 27599, USA; Department of Genetics, University of North Carolina, Chapel Hill, NC 27599, USA; UNC Neuroscience Center, University of North Carolina, Chapel Hill, NC 27599, USA; Mark and Mary Stevens Neuroimaging and Informatics Institute, Keck School of Medicine, University of Southern California, Marina del Rey, CA 90292, USA; Psychiatric Genetics, QIMR Berghofer Medical Research Institute, Brisbane, QLD 4006, Australia; Department of Psychiatry and Human Behavior, University of California, Irvine, CA 92697, USA; Department of Medicine, Section of Genetic Medicine & Institute for Genomics and Systems Biology, University of Chicago, Chicago, IL 60637, USA; Department of Pharmacology, Center for Genetic Medicine, Northwestern University Feinberg School of Medicine, Chicago, IL 60611, USA; Department of Medicine, Division of Medical Genetics, Vanderbilt University Medical Center, Nashville, TN 37232, USA; Department of Psychiatry and Behavioral Sciences, Vanderbilt University Medical Center, Nashville, TN 37232, USA; Vanderbilt University Medical Center, Vanderbilt Genetics Institute, Nashville, TN 37232, USA; Language and Genetics Department, Max Planck Institute for Psycholinguistics, Nijmegen, 6500 AH, Netherlands; Donders Institute for Brain, Cognition and Behaviour, Radboud University, Nijmegen, 6500 HB, Netherlands; Department of Genetics, University of North Carolina, Chapel Hill, NC 27599, USA; UNC Neuroscience Center, University of North Carolina, Chapel Hill, NC 27599, USA

**Keywords:** cortical surface area, genome-wide association study, human gained enhancers, polygenic selection

## Abstract

Structural brain changes along the lineage leading to modern *Homo sapiens* contributed to our distinctive cognitive and social abilities. However, the evolutionarily relevant molecular variants impacting key aspects of neuroanatomy are largely unknown. Here, we integrate evolutionary annotations of the genome at diverse timescales with common variant associations from large-scale neuroimaging genetic screens. We find that alleles with evidence of recent positive polygenic selection over the past 2000–3000 years are associated with increased surface area (SA) of the entire cortex, as well as specific regions, including those involved in spoken language and visual processing. Therefore, polygenic selective pressures impact the structure of specific cortical areas even over relatively recent timescales. Moreover, common sequence variation within human gained enhancers active in the prenatal cortex is associated with postnatal global SA. We show that such variation modulates the function of a regulatory element of the developmentally relevant transcription factor *HEY2* in human neural progenitor cells and is associated with structural changes in the inferior frontal cortex. These results indicate that non-coding genomic regions active during prenatal cortical development are involved in the evolution of human brain structure and identify novel regulatory elements and genes impacting modern human brain structure.

## Introduction

**Figure 1:**
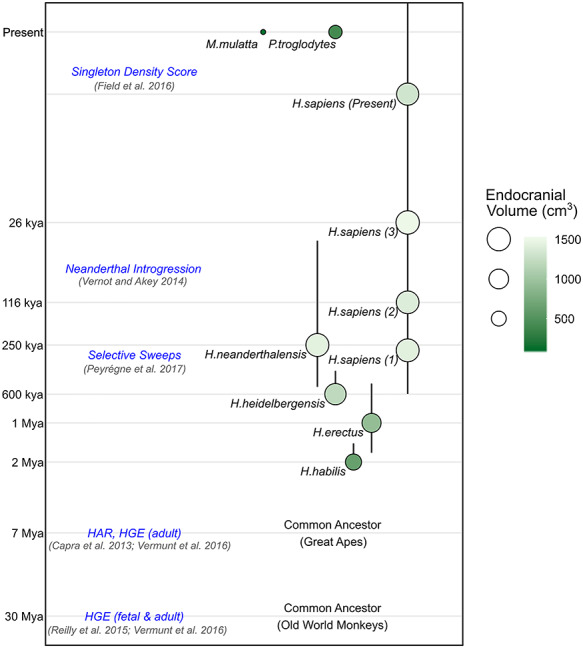
Overview of human evolution and timeframes captured by different sets of analyses in this study. Size and shading of circles indicates average endocranial volume [*H. habilis* volume from Klein (2009), all other hominin volumes are averaged from Neubauer et al. (2018). *P. troglodytes* volume from Neubauer et al. (2012) and *M. mulatta* volume is an average from Isler et al. (2008)], and their vertical position indicates the age of the specimen [an average in the case of the Neubauer et al. (2018) hominin endocasts]. Numbers next to the *H. sapiens* circles indicate the geologic age group from Neubauer et al. (2018). Vertical lines reflect the approximate timeframe of the hominin species (right). Evolutionary time is presented in log_10_ scale (Mya, million years ago; kya, thousand years ago). Different types of evolutionary annotations are indicated, identifying genomic loci that underwent changes over different time frames (left, blue text). Horizontal spacing between species does not convey genetic distance, geological time, or any other metric.

The size, shape, and neural architecture of the modern human brain reflect the cumulative effects of selective pressures over evolutionary history. Analyses of fossilized skulls indicate that endocranial volume has increased dramatically on the lineage that led to *Homo sapiens* in the over 6 million years since our last common ancestor with chimpanzees (Fig. 1; Henneberg 1988; Lee and Wolpoff 2003; Jantz and Jantz 2016; Moorjani et al. 2016; Du et al. 2018). It is thought that these volumetric increases were mainly driven by expansions of neocortical surface area (SA) (Rakic 2009; Lui et al. 2011; Geschwind and Rakic 2013), although changes in other brain structures, including the cerebellum, also likely played a significant role (Barton and Venditti 2014; Miller et al. 2019). Beyond overall size differences, skull endocasts of archaic hominins suggest that human-specific refinements to brain structure occurred during the last 300 000 years, most notably the shift toward a more globular shape (Hublin et al. 2017; Gunz et al. 2019). A commonly held view is that differential expansion of distinct regions of the neocortex contributed to the evolution of the distinctive cognitive and social abilities of our species (Rakic 2009; Lui et al. 2011; Geschwind and Rakic 2013). Neuroanatomical changes in our ancestors were accompanied by increasingly sophisticated tool use, the emergence of proficient spoken language, world-wide migrations, and the development of agriculture, among other innovations (Pääbo 2014).

Several studies have identified fixed genomic differences that may have impacted aspects of brain structure along our lineage (Enard 2016; Sousa et al. 2017; Mitchell and Silver 2018), but the genetic variation that shaped the cortex across human evolution is still largely undetermined. In the present study, we adopt a novel strategy to uncover genetic variants that have contributed to anatomical features of the modern human brain. To do so, we identify loci of defined evolutionary relevance in the genome and assess the effects of those loci on cortical structure through large-scale neuroimaging genetics. Comparative genomic and population genetic annotations from multiple sources have been used to identify evolutionarily relevant loci in the human genome across diverse time scales (Fig. 1; Pollard, Salama, King, et al. 2006a; Vernot and Akey 2014; Reilly et al. 2015; Field et al. 2016; Simonti et al. 2016; Vermunt et al. 2016; Nielsen et al. 2017; Peyrégne et al. 2017). Two annotations of particular note capture distinct periods in human history. The singleton density score (SDS) uses genome sequencing data to identify haplotypes with a decreased accumulation of singleton variants in the population being studied, providing evidence for polygenic natural selection acting over the past ~2000–3000 years (Field et al. 2016). On a deeper time scale, human-gained enhancers (HGEs) represent gene regulatory elements that display stronger histone acetylation or methylation marks of promoters or enhancers in human cortical tissue compared with extant primates or mice (Reilly et al. 2015; Vermunt et al. 2016), arising after our last common ancestor with Old World monkeys about 30 million years ago (Mya).

By themselves, these indices suggest loci of likely evolutionary significance in the human genome but are not informative for defining which loci (if any) influence the structure of the human brain. We hypothesize that, for evolutionarily relevant genetic variants that have not reached fixation, data from genome-wide association studies (GWAS) of cortical structure can help determine their potential functional impacts on brain structure. We reason that GWAS data may shed light on the evolution of cortical structure by: 1) determining if alleles under selective pressure are associated with variation in neural anatomy and 2) revealing if interindividual variation in defined genomic regions of evolutionary significance is associated with variation in neural anatomy. Crucially, this novel approach for studying human brain evolution depends on the availability of large datasets of many thousands of individuals in which structural neuroimaging measures have been coupled to genome-wide genotyping. In this regard, we take advantage of recent large-scale GWAS work from the Enhancing NeuroImaging Genetics through Meta Analysis (ENIGMA) consortium (Grasby et al. 2020), including data from the UK Biobank (Elliott et al. 2018), which identified hundreds of genetic loci associated with interindividual variability in human cortical structure in living populations. Thus, here, we integrate genomic annotations spanning 30 million years of our evolutionary history with data from a GWAS meta-analysis of cortical SA in over 33 000 modern humans (Grasby et al. 2020) to assess the aggregate impact of each annotation on modern variation in cortical SA and identify genetic variants within these annotations with notable effects on human neural development.

## Materials and Methods

### Genome-Wide Association Summary Statistics

Summary statistics for 35 cortical SA phenotypes (global SA and average bilateral SA for 34 regions) were obtained from a European ancestry discovery sample of the ENIGMA cortical SA meta-analysis (Grasby et al. 2020) including data from the UK Biobank (UKBB) (Elliott et al. 2018). For comparative purposes, corresponding summary statistics for cortical thickness were also obtained from the same source. We focused our analyses on SA given its particular expansion during hominid evolution, well established in prior literature, but as a comparison also show results from analyses of thickness in the Supplementary Materials. Details of image segmentation, genotyping, imputation, association, and meta-analysis are found in the primary GWAS meta-analysis reference (Grasby et al. 2020). Briefly, magnetic resonance images of the brain were segmented with FreeSurfer (Dale et al. 1999) using a gyrally defined atlas (Desikan et al. 2006), and visually quality checked based on guidelines provided at the ENIGMA website (http://enigma.ini.usc.edu/research/gwasma-of-cortical-measures/). Imputation of genome-wide genotyping arrays was conducted to the 1000 Genomes phase 1 release v3 reference panel. When conducting associations of gyrally defined regions, the global measure of SA was included as a covariate, in order to test for genetic influences that were specific to each region. The original association models also included four multidimensional scaling components to help control for ancestry, as well as linear and nonlinear corrections for age and sex, diagnostic status, and scanner. Fixed effects meta-analysis was used to combine effects across all sites contributing to the analysis (Willer et al. 2010). All analyses were performed on summary statistics without genomic control (Bacanu et al. 2000) correction applied.

### Ancestry Regression

**Figure 2:**
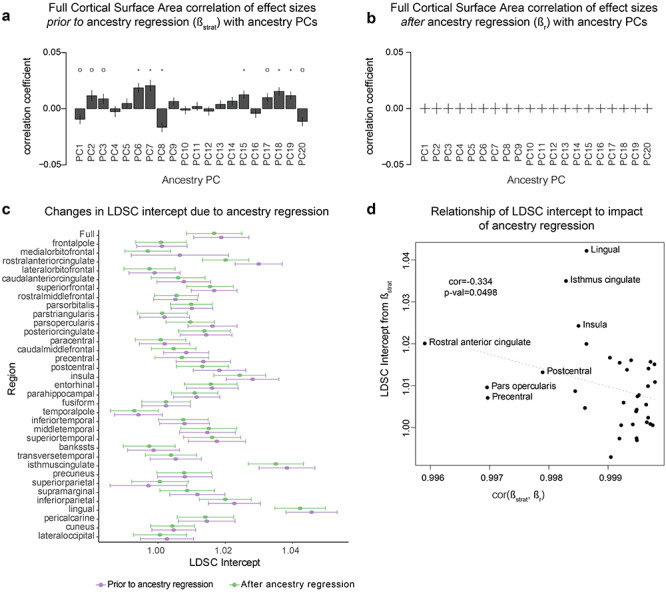
Identifying and correcting for the effects of subtle population stratification on GWAS results. (*a*) Correlations between SNP loadings on ancestry PCs (Beta_PCs) and GWAS effect sizes for full SA (Beta_Strat) demonstrate evidence for subtle population stratification (* indicates Bonferroni corrected significant correlation *P*-value < 0.0025, and o indicates a nominally significant correlation, *P*-value < 0.05). (*b*) Subtle population stratification is reduced after ancestry regression. (*c*) LD-score regression (LDSC) intercepts, standard measures of population stratification, are generally decreased after ancestry regression. An absence of population stratification and cryptic relatedness would be indicated by an LDSC intercept value of 1. (*d*) There is an inverse relationship between the degree of subtle population stratification (LDSC intercept prior to ancestry regression) and the amount of change caused by ancestry regression (cor[Beta_strat, Beta_r]). Error bars represent standard errors.

We first determined the impact of subtle population stratification on each GWAS summary statistics dataset, in light of studies showing that such stratification can confound estimates of selection (Berg et al. 2018; Sohail et al. 2019). First, all unrelated subjects [defined in Gazal et al. (2015)] were selected from 1000 Genomes Phase 3 data (1000 Genomes Project Consortium et al. 2015). We then selected single nucleotide polymorphisms (SNPs) that had a minor allele frequency (MAF) > 5% in 1000 Genomes and that were not located in the major histocompatibility complex (MHC) locus, the chromosome 8 inversion region, or regions of long linkage disequilibrium (LD). LD-independent SNPs (*r*^2^ < 0.2) were selected via pruning using a window size 500 kb and a slide of 100 kb (PLINK—indep-pairwise 500 100 0.2). Principal component (PC) analysis was performed in PLINK (Chang et al. 2015) on the 264 339 remaining SNPs. In order to obtain SNP PC loadings for all SNPs in the 1000 genomes project (MAF < 0.05, MHC locus, the chromosome 8 inversion region, or regions of long LD removed), we performed linear regressions of the PC scores on the genotype allele count of each SNP (after controlling for sex) and used the resulting regression coefficients as the SNP PC loading estimates. This procedure followed that used in previous work (Sohail et al. 2019). For the first 20 PCs, the weighting of the PCs for each subject was used as a trait and tested for association with each subject’s genotype in PLINK. For each SNP, across all 20 PCs, we identified the degree of association of that SNP to population frequency differences along that principal axis of variation (Beta_PCs). After merging summary statistics of each SA GWAS without genomic control (Bacanu et al. 2000) correction (Beta_strat) with Beta_PC values, ensuring beta values were with respect to the same effect allele, and sorting based on chromosomal position, a block jackknife correlation with 1000 blocks approach was used to assess the correlation between Beta_strat and Beta_PCs, shown in Figure 2*a* and Supplementary Figure 1.

We then implemented an ancestry regression procedure following previous work (Bhatia et al. 2016). We used a regression model fitting each set of SA GWAS summary statistics without genomic control correction (Beta_strat) simultaneously to the 20 Beta_PC values calculated as described above using the lm() function in R (v3.2.3). The residuals of this model (Beta_r) were used as ancestry-corrected effect sizes. Ancestry-corrected standard errors and *P*-values were calculated following the same prior work (Bhatia et al. 2016). The same block jackknife correlation method was used to assess the impact of subtle population stratification by correlating Beta_r with Beta_PC in Figure 2*b* and Supplementary Figure 2. The same analyses were completed for cortical thickness (Supplementary Figs 3 and 4).

We evaluated an additional measure of population stratification, the LD-score regression (LDSC) intercept (Bulik-Sullivan, Finucane, et al. 2015a), before and after ancestry regression (Fig. 2*c*, Supplementary Fig. 5). The summary statistics (with or without ancestry regression, as above) were first written into a standard format using munge_sumstats.py. Then, precomputed LD scores from 1000 Genomes Phase 3 (using only HapMap3 SNPs, excluding the MHC region) were downloaded from the LDSC website (https://github.com/bulik/ldsc) and implemented according to the guidelines given there.

### Genetic Correlations

Genetic correlations of ancestry-regressed cortical structure with height (Supplementary Fig. 6) were calculated using LDSC regression (Bulik-Sullivan, Finucane, et al. 2015a). Summary statistics for height were acquired from previously published work (Wood et al. 2014).

### SDS Implementation

**Figure 3:**
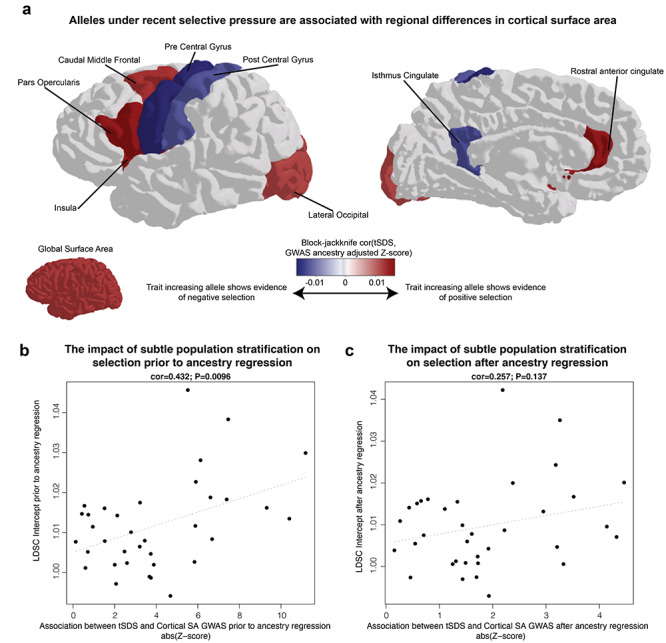
Evidence for haplotypes under recent polygenic selection (~2000–3000 years) impacting cortical structure. (*a*) A block-jackknife correlation of ancestry regressed effect sizes from GWAS (*Z*-scores) with scores of recent selection (tSDS) demonstrates evidence for polygenic alleles under selective pressure also influencing both global and regional SAs (colored regions indicate FDR < 0.05). Colder colors indicate that the trait increasing alleles (associated with increased SA) are generally associated with negative selection (decreasing allele frequencies in the population), whereas warmer colors indicate that trait increasing alleles are associated with positive selection. (*b*) Subtle population stratification, measured via LD-score regression (LDSC) intercept, is associated with stronger evidence of selection prior to ancestry regression. (*c*) Ancestry regression reduces the relationship between measures of selection and population stratification.

SDSs (Field et al. 2016) for each SNP were downloaded from https://datadryad.org/resource/doi:10.5061/dryad.kd58f. Ancestry-regressed summary statistics (without any significance thresholding) were merged with SDS scores by rsID and ensured that the SDS value describes the trait increasing allele (tSDS). The ancestry-regressed *Z*-score was calculated as the ancestry-regressed beta divided by the ancestry-regressed standard error. Merged files were then sorted by chromosomal position, and block jackknife Spearman’s correlation with 100 blocks was used to determine the relationship between ancestry-regressed *Z*-scores and tSDS values. The Benjamini Hochberg false discovery rate (FDR) correction was used to correct for multiple comparisons across each of the 35 GWASs used. Results were plotted on a representative brain surface using the R/plotly package, where the correlation values were only shown for significant associations after FDR correction (FDR adjusted *P*-value < 0.05; see Fig. 3*a*). These analyses were run in two additional ways: 1) without ancestry regression on the full ENIGMA SA GWASs in Figure 3*b*; and 2) without ancestry regression in the UKBB dataset subset to only European individuals, which is less susceptible to the impact of population stratification due to the combination of effects across many sites, as in the larger ENIGMA analysis (*N* = 9923; Supplementary Fig. 7). The same analyses were also completed for cortical thickness in Supplementary Figure 8.

### Partitioned Heritability

The contributions of each SNP set to the total SNP heritability of each trait were determined using partitioned heritability analyses as implemented in the LDSC software package (Finucane et al. 2015). Enrichment of heritability within HARs (Capra et al. 2013), selective sweep regions (Peyrégne et al. 2017), Neanderthal-introgressed SNPs (Vernot and Akey 2014), and Neanderthal-depleted regions (Vernot et al. 2016) all controlled for the baselineLD v2 model from the original LDSC study (Finucane et al. 2015). Heritability enrichment in fetal brain HGEs (Reilly et al. 2015) controlled for both the baseline model and a set of fetal brain active regulatory elements (E081) from the Epigenomics Roadmap resource. Heritability enrichment in adult brain HGEs (Vermunt et al. 2016) controlled for both the baseline model and adult brain active regulatory elements (E073) from the Epigenomics Roadmap resource. Active regulatory elements were defined using chromHMM (Ernst and Kellis 2012) marks from the 15 state models including all the following annotations: 1_TssA, 2_TssAFlnk, and 7_Enh, 6_EnhG.

### Gene Annotations

Gene sets impacted by genetic variation within any HGE were derived separately for 1) global SA or 2) any of the 34 regional SA loci. We first identified all SNPs within 10 000 kb of a nominally significant (*P*-value < 5 × 10^−8^) GWAS locus with *r*^2^ > 0.6 in the 1000G EUR population to the index SNP, using PLINK 1.9. With this extended list of SNPs in LD with the GWAS index SNP, we looked for overlaps with HGEs defined in any human brain region or developmental time period (Reilly et al. 2015). For those genome-wide significant loci that also overlapped with HGEs, we then recorded known functional impacts on gene expression using adult brain expression quantitative trait loci (eQTLs) from the PsychENCODE dataset (Wang et al. 2018), downloaded from http://adult.psychencode.org/ selecting the dataset thresholded by the following parameters (FDR < 0.05, expression > 0.1 FPKM in at least 10 samples). Gene biotype annotations (e.g., protein coding) were called using ENSEMBL via biomaRt.

Pathway enrichment was performed for each gene list using the *gost* function from the “gprofiler2” package (version 0.1.3). Electronic gene ontology (GO) annotations (evidence code IEA) were excluded, the sources were limited to GO, KEGG, and Reactome pathways, and FDR correction was applied with a significance threshold of 0.05.

### Chromatin Accessibility Quantitative Trait Locus (caQTL) Mapping at the HEY2 Locus

caQTL data were acquired from our previous work (Liang et al. 2020). Briefly, we generated chromatin accessibility profiles from primary human neural progenitor cell lines (*N*_donors cultured_ = 73) and their differentiated neuronal progeny (*N*_donors cultured_ = 61) using ATAC-seq (Buenrostro et al. 2013). We genotyped the same cell lines using an Illumina HumanOmni2.5 or HumanOmni2.5Exome platform and imputed to 1000 Genomes Phase 3 reference panel. We performed a caQTL analysis separately for progenitors and neurons using a mixed effects model including a kinship matrix for SNPs 100kb up- and downstream from the center of each chromatin accessibility peak. Allele-specific chromatin accessibility was performed in DESeq2 (Love et al. 2014) after utilizing WASP to reduce mapping bias (van de Geijn et al. 2015).

### Data Visualization

**Figure 4:**
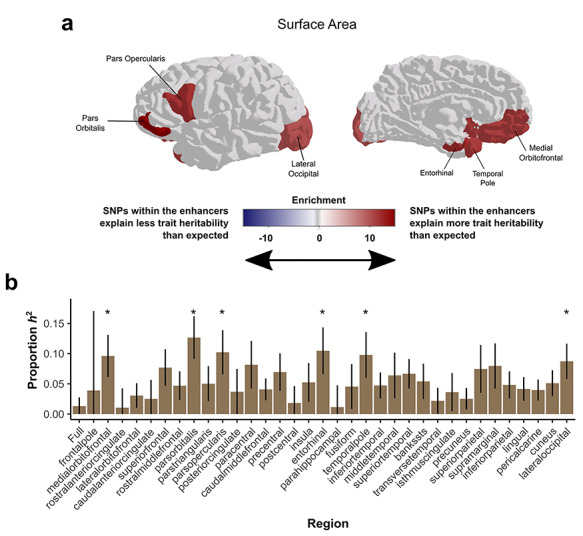
SNPs within HGEs active at 7 weeks postconception explain a significant proportion of the heritability of SA for multiple brain regions. (*a*) Shading indicates the enrichment values of SNP-heritability explained by HGEs active at 7 weeks postconception for the SA of each region, nonsignificant values are shaded in gray. (*b*) Proportion of SNP-heritability explained by 7 PCW HGEs for SA of each cortical region. Asterisks label regions with FDR-corrected *P*-values < 0.05, error bars represent standard errors.

Genomic loci plots were constructed using the R package “GViz”, with evolutionary annotation data sourced from the references given in Data Availability. Brain plots (Figs 3 and 4) were made using the “plotly” package. All other plots were made in R using “ggplot2” and related packages.

### Data and Code Availability

Code used to perform analyses is available at https://bitbucket.org/jasonlouisstein/enigmaevolma6/src/master/. Genomic regions that underwent rapid change on the human lineage (human accelerated regions, HARs) were combined from several sources (Pollard, Salama, Lambert, et al. 2006b; Prabhakar et al. 2006; Bird et al. 2007; Bush and Lahn 2008; Lindblad-Toh et al. 2011). BED files listing fetal brain enhancer elements not found in macaques or mice were obtained from previous work (Reilly et al. 2015). Adult brain enhancer elements arising since our last common ancestor with the macaque or chimpanzee were obtained from (Vermunt et al. 2016). A refined list of SNPs gained through introgression with Neanderthals was obtained from previous work (Simonti et al. 2016). Genomic regions depleted of introgressed Neanderthal DNA were obtained from previous work (Vernot et al. 2016). Ancient selective sweep regions identified using extended lineage sorting were obtained from previous work (Peyrégne et al. 2017). A summary of all annotations is found in Supplementary Table 1.

## Results

### Reducing the Impact of Subtle Population Stratification

The ENIGMA consortium recently conducted a GWAS meta-analysis identifying hundreds of common variants associated with variability in SA and cortical thickness in European populations (*N* = 33 992 individuals from cohorts across the lifespan) (Grasby et al. 2020). Given the massive expansion of SA in modern humans and only subtle increases in cortical thickness as compared with extant mammalian species (Rakic 2009), we chose SA as the primary focus for the present study. Nevertheless, for comparative purposes, we performed a matching set of analyses for thickness associations, and these are shown in the Supplementary Materials.

Population stratification is the existence of systematic differences in allele frequencies between populations. Unbalanced representations of multiple populations in genetic association studies can lead to false-positive findings that are driven by allele frequency differences between populations rather than true association with a trait (Balding 2006). Moreover, subtle population stratification in GWAS statistics can inflate the assessment of polygenic selection impacting a trait (Berg et al. 2018; Novembre and Barton 2018; Barton et al. 2019; Sohail et al. 2019). We first tested whether subtle population stratification was influencing meta-analysis effect sizes in the cortical GWAS data, even after applying the accepted standard correction for multidimensional scaling components of ancestry prior to meta-analysis (Grasby et al. 2020). PC analysis enabled us to identify major axes of variation in allele frequency across current human populations using unrelated individuals of all ancestries from the 1000 Genomes Phase 3 data. Then, we tested the association of each SNP to the top 20 PCs (each treated as a separate trait) within the 1000 Genomes population, yielding an estimate of the degree to which each SNP contributes to population frequency differences along each principal axis of variation (Beta_PCs) (Sohail et al. 2019). Finally, using Pearson’s correlation, the Beta_PCs were correlated with the effect sizes from the GWAS meta-analysis for each trait, which may be impacted by population stratification (Beta_Strat). To assess the significance of the correlation in the context of LD, a block jackknife approach was employed to calculate the standard errors for the correlation (Kunsch 1989; Busing et al. 1999). Significant correlations between Beta_PCs (consistent allele frequency differences differentiating human populations) and Beta_Strat (effect sizes of variants on human brain structure from GWAS) are indicative of subtle, uncorrected population stratification (Berg et al. 2018; Sohail et al. 2019). As shown in Figure 2*a*, we detected significant relationships between Beta_Strat and PCs 6, 7, 8, 15, 18, and 19 for global SA, indicating subtle residual population stratification affecting the GWAS summary statistics. This analysis also showed subtle population stratification affecting summary statistics for each of the regional SAs, to varying degrees (Supplementary Fig. 1). We note that another measure of population stratification, the LDSC intercept (Bulik-Sullivan, Loh, et al. 2015b), gave values that were uniformly less than 1.05 (a commonly used threshold for ruling out stratification) for global SA and all regional SAs (Fig. 2*c*).

To correct for this subtle population stratification, we implemented an ancestry regression procedure based on GWAS summary statistics (Bhatia et al. 2016). The residuals (Beta_r) of a model fitting GWAS effect sizes (Beta_Strat) with the first 20 PC weightings (Beta_PC) were used as ancestry-corrected estimates of effect sizes. As expected, these ancestry-corrected estimates (Beta_r) showed much reduced correlations with PC weights (Fig. 2*b*, Supplementary Fig. 2). Additionally, LDSC intercepts for phenotypes after ancestry regression were generally slightly decreased, consistent with diminished effects of subtle population stratification on common variant associations to SA (Fig. 2*c*). Furthermore, correlations between effect size measurements after ancestry regression (Beta_r) and effect size measurements prior to ancestry regression (Beta_Strat) were all extremely high, indicating that ancestry regression did not strongly change the association statistics (correlations all >0.995; Fig. 2*d*). There were 343 genome-wide significant loci (*P*-values < 5 × 10^−8^; clumping *r*^2^ < 0.2) prior to ancestry regression impacting global SA or any of the regional SAs and 303 genome-wide significant loci after ancestry regression. Finally, those brain regions that showed the highest LDSC intercepts prior to ancestry regression, indicative of being most affected by subtle residual population stratification, were also those that showed the largest changes in GWAS effect sizes following ancestry regression (*r* = −0.334; *P*-value = 0.0498; Fig. 2*d*). The ancestry regression procedure was also carried out for cortical thickness GWAS (Supplementary Figs 3–5). For all subsequent analyses, we used the ancestry-corrected effect size estimates, standard errors, and *P*-values, thereby minimizing the impact of population stratification on the results of our evolutionary assessments.

### Specificity of GWAS Results to Brain versus Body Size

To investigate whether GWAS results of cortical structure revealed specific influences on the brain as compared with global body size, we performed genetic correlations with a GWAS of height (Wood et al. 2014). As was noted in previous work, and shown in Supplementary Figure 6, there is a partially shared genetic basis between height and global cortical SA (*r*_g_ = 0.21) (Grasby et al. 2020). However, our previous work also indicated the genetic correlations between intracranial volume controlling for height and global SA (*r*_g_ = 0.81) are much stronger than genetic correlations between height and global cortical SA (*r*_g_ = 0.21), which demonstrates that the genetic signal discovered in our global cortical SA GWAS is mostly brain specific and not driven entirely by body size (Grasby et al. 2020). In the association model of each of the 34 cortical regions, we control for global SA to identify specific effects on that region, so we do not expect to observe a large degree of shared genetics with body size. Indeed, we did not observe any significant (FDR < 0.05) genetic correlations between height and the 34 regional SA measurements (Supplementary Fig. 6). We performed the same analyses for thickness and only detected one region with a significant (FDR < 0.05) genetic correlation with height, inferior temporal gyrus, which was not implicated in any of our subsequent evolutionary analyses. In sum, our findings are largely brain-specific.

### Evidence for Polygenic Selection Impacting Human Cortical Structure

In our evolutionary analyses, we first assessed how alleles that show evidence of recent selective pressure impact cortical SA. The SDS reveals haplotypes under recent positive/negative selection in the human genome by identifying those that harbor fewer/greater singleton variants (presumed to have arisen recently) near any given SNP (Field et al. 2016). This metric, together with data from a suitable GWAS, can be used to infer whether a trait of interest has been subject to highly polygenic selection on an evolutionarily recent timescale, over the past ~2000–3000 years. We found that alleles with evidence of positive selection over this recent timescale have a small but detectable influence on increasing global SA in the GWAS datasets (block jackknife correlation = 0.0129, FDR adjusted *P*-value = 0.0038; Fig. 3*a*). In addition, our results showed that alleles undergoing polygenic selection over the past ~2000–3000 years are associated with variation in cortical SA of individual gyrally defined brain regions (Fig. 3*a*). Notably, based on the cortical region-specific GWASs, there is a detectable relationship between alleles under positive polygenic selective pressure (increasing in allele frequency over time) and increased cortical SA in regions known to be important for speech/language functions (*pars opercularis*, part of the inferior frontal gyrus) and visual processing (lateral occipital cortex). Conversely, alleles under negative polygenic selective pressure (decreasing allele frequency over time) are associated with increased cortical SA in the pre- and postcentral gyrus, regions involved in somatosensation and movement.

We conducted secondary analyses to investigate the potential impacts of the subtle population stratification, described above, because it was recently shown that SDS correlations can be highly influenced by this confounder (Berg et al. 2018; Sohail et al. 2019). Exploratory analyses of GWAS data that were uncorrected for ancestry showed a clear relationship between the LDSC intercept (a measure of the degree of population stratification) and the level of correlation between SDS and the GWAS *Z*-scores (cor = 0.432, *P*-value = 0.0096; Fig. 3*b*). In contrast, for analyses of GWAS data that had undergone ancestry regression, there was no significant relationship between SDS and GWAS *Z*-scores (cor = 0.257, *P*-value = 0.137; Fig. 3*c*), indicating that the ancestry regression procedure is effective in diminishing confounding effects of population stratification. We note that although the ancestry regression procedure attenuates the signals of polygenic selection impacting SA, nevertheless several regions are robust to such adjustment (FDR < 0.05 are colored and labeled in Fig. 3*a*). Finally, we show that SDS correlations within the UKBB European population alone, which is less susceptible to the impacts of subtle population stratification than meta-analysis of data from consortia (Berg et al. 2018; Sohail et al. 2019), show a highly consistent SDS relationship with the ancestry-corrected meta-analysis results (cor = 0.635, *P*-value = 4.2 × 10^−5^; Supplementary Fig. 7).

After implementing the ancestry regression procedures, we also performed the evolutionary analyses on cortical thickness. We found no significant correlation between global thickness and selective pressures over the past 2000–3000 years. We detected two significant associations between recent selective pressures and cortical thickness in the precuneus and superior parietal cortex. In these regions, alleles inferred to have increased in frequency over the past 2000–3000 years are associated with increased cortical thickness (Supplementary Fig. 8). These regions have been independently proposed in prior studies as relevant for human brain evolution (Bruner et al. 2017; Pereira-Pedro et al. 2020).

### Significant Heritability Enrichment within HGEs (30 Mya)

We went on to assess deeper evolutionary time scales, targeting human fetal brain enhancer elements that emerged since our last common ancestor with macaques, commonly referred to in the literature as HGEs (Reilly et al. 2015). These elements were detected by comparing post-translational modifications of histone tails indicative of enhancers and promoters (H3K27ac and H3K4me2) across humans, macaques, and mice. Using brain tissue from similar developmental time points across the three species, regulatory elements (peaks in the histone modification signals) were identified that were present in human fetal brain at 7 postconception weeks (PCW), but to a significantly lesser degree in developing macaque or mouse brain tissue (Reilly et al. 2015). The enhancer activity of HGEs has recently been experimentally tested using a multiplex parallel reporter assay in human neural progenitor cells (Uebbing et al. 2019). In this assay, 43% of HGEs were found to be active enhancers, providing important experimental validation that histone post-translational modification marks are functionally active. To understand how these HGEs influence cortical SA in modern humans, we measured their relative contribution to total SNP heritability. A trait’s SNP-based heritability is the total amount of variance in the trait (e.g., global SA) that can be attributed to common variation across the genome, and it can be estimated from GWAS summary statistics. This genome-wide SNP heritability can be partitioned into categories to measure how specific genomic regions of interest (in this case, evolutionary annotations) contribute to the heritability of the trait.

We assessed how common variants within HGEs contribute to the SNP-based heritability of cortical SAs, testing for enrichment using LDSC partitioned heritability (Finucane et al. 2015), with FDR correction for the 35 traits (34 regions plus global SA). Furthermore, because SNPs within regulatory elements that are active during fetal development are known to make significant impacts on both intracranial volume and cortical SA (de la Torre-Ubieta et al. 2018; Grasby et al. 2020), we controlled for a global category of fetal brain active regulatory elements [derived from the Epigenomics Roadmap (Roadmap Epigenomics Consortium et al. 2015)] in the analysis. This is in addition to the 97 categories included in the baselineLD v2 model, which span a wide range of functional elements. These additional control categories make it possible to assess the contribution of evolution-focused annotations with a high degree of specificity. SNPs within HGE elements made significantly enriched contributions to cortical SA heritability for 6 out of 34 gyrally defined regions after controlling for global SA (Fig. 4*a*). The enrichment signal was strongest for the *pars orbitalis*, part of the inferior frontal gyrus (Enrichment = 14.96, FDR corrected *P*-value = 0.0053). As the regional GWAS results were controlled for global SA, the heritability enrichment signals detected in each region are independent of global SA. Our findings indicate that SNPs within these HGEs have effects beyond those of general fetal enhancers. Altogether, the data suggest that a key set of neural enhancer regions that became functional since our split from Old World monkeys contribute an unusually large amount to the heritability of regional cortical SA in adult humans. This influence on SA in the adult brain may be realized through common genetic variation within these HGEs impacting gene regulation during fetal brain development. In order to assess the specificity of these findings to brain-related phenotypes, we tested heritability enrichment of irritable bowel disease (Jostins et al. 2012) for these evolutionary annotations, as it is a non-neural human trait with a GWAS meta-analysis of comparable sample size to the cortical structure GWASs. We found no significant enrichments across the same set of evolution-focused annotations, applying the same controls described above (Supplementary Table 3). The same partitioned heritability analysis was performed for global and regional cortical thickness, but no significant enrichment was identified (Supplementary Fig. 10).

### Other Classes of Evolutionary Annotations are not Enriched for Cortical SA or Thickness Heritability

We examined the contributions of several other evolution-focused annotations (namely, HGEs active in the adult brain based on comparison to either macaque or chimpanzee, human accelerated regions, selective sweeps, and Neanderthal introgressed or depleted regions) to the heritability of cortical SA, finding no significant positive enrichment (Supplementary Fig. 9). The results suggest that these particular sets of genomic regions do not contribute more to the heritability of cortical SA than expected, given their size.

The same partitioned heritability analysis was performed for global and regional cortical thickness, with the only positive enrichment surviving FDR correction being for Neanderthal lineage depleted regions in the superior parietal region (Enrichment = 0.20, FDR-corrected *P*-value = 0.042, Supplementary Fig. 10).

**Figure 5:**
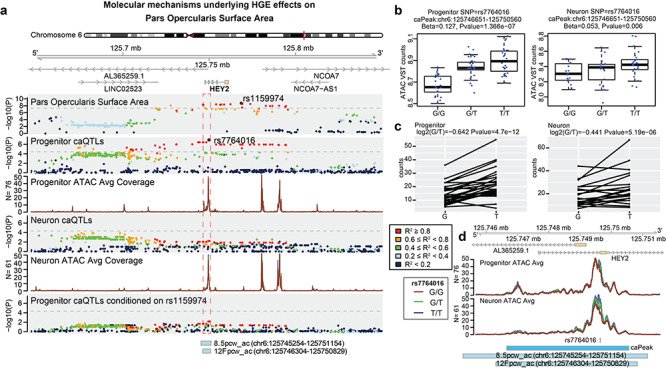
Fine mapping and molecular mechanisms underlying genetic variation at HGE associated with SA of *pars opercularis* in the inferior frontal gyrus. (*a*) Regional plots showing associations to *pars opercularis* SA, associations to the boxed chromatin accessibility peak in human neural progenitors, the average chromatin accessibility in progenitors, association to the boxed chromatin accessibility peak in differentiated neurons, the average chromatin accessibility in neurons, and an analysis demonstrating a co-localization through observation that controlling for the *pars opercularis* SA index SNP abolishes the progenitor caQTL signal. The *y*-axis on the nonshaded tracks represents chromatin accessibility as average normalized read density across ATAC-seq libraries for either neurons or progenitors. (*b*) Boxplots demonstrating the caQTL association observed in progenitors and neurons for the index caQTL SNP. (*c*) Allele specific chromatin accessibility is also observed at the same SNP. (*d*) Chromatin accessibility separated by genotype at the boxed peak in (*a*) overlapping the human-gained enhancer peaks. The blue bar represents the chromatin accessibility peak defined in this dataset and the gray bars represent human gained enhancer peaks. The *y*-axis again represents chromatin accessibility as average normalized read density across ATAC-seq libraries. Prior to ancestry regression, the SNP rs7764016 has association to *pars opercularis* SA with *P* = 2.1e−09. However, this SNP is not present in the 1000 Genomes Phase 3 data used for ancestry regression so is not present as a dot in (*a*).

### Linking GWAS Results, Regulatory Elements, Genes, and Evolutionary History

To further understand how gene regulation is impacted by common variation within HGEs, we established which of the genome-wide significant SA loci (*P*-value < 5 × 10^−8^ including SNPs in LD at *r*^2^ > 0.6) fall within HGEs and also modulate gene expression in adult cortical tissue (Wang et al. 2018) (expression quantitative trait loci—eQTLs—at FDR < 0.05). Seven of twenty-four genome-wide significant global SA loci overlapped (directly or with an LD-associated SNP) with an HGE. Four of those seven loci also have a significant eQTL impacting 18 protein-coding genes, *eGenes*, defined as the genes whose expression is associated with the genetic variation. These eGenes included developmentally relevant genes *FOXO3*, *ERBB3*, and *WNT3* (a full list is found in Supplementary Table 2). One SNP in LD with rs2802295 (rs9400239, *r*^2^ = 0.715), associated with global SA, maps to a 7 PCW fetal brain HGE and is located within an intron of the *FOXO3* gene on chromosome 6q21. The derived allele (G) at rs2802295 is associated with increased global cortical SA. The Human Genome Dating atlas estimates the derived allele to be 26 353 (23 115.3–29 770.7 95% confidence interval) generations old (Albers and McVean 2020). Assuming 25 years per generation, the estimated age of the derived allele is 658 (578–744) kya. rs2802295 has also been associated with interindividual variation in general intelligence (Sniekers et al. 2017) (marked by rs2490272 index SNP, *r*^2^ = 1.0 with rs2802295), with the SA increasing allele also associated with higher scores on tests of intelligence. The SNP also functions as a cortical eQTL for *FOXO3* [FDR adjusted *P*-value = 0.0051, derived from the adult brain PsychENCODE dataset (Wang et al. 2018)]. *FOXO3* encodes a transcription factor that regulates neuronal stem cell homeostasis (Renault et al. 2009), among other roles. Considering the 279 genome-wide significant regional SA loci, there were 46 that overlapped (directly or with an LD-associated SNP) with an HGE. Out of those 46 loci, 30 also have a significant eQTL, impacting a total of 47 protein-coding eGenes. These eGenes include known genes involved in areal identity including *LMO4* (Huang et al. 2009) as well as developmentally relevant transcription factors like *HEY2* (a full list is found in Supplementary Table 2).

We focused on understanding potential mechanisms by which evolutionarily relevant genetic variation may be associated with changes in inferior frontal brain structure, given this region’s strong HGE partitioned heritability enrichment and involvement in language. For a locus significantly associated with *pars opercularis* SA, 26 SNPs in LD (*r*^2^ > 0.6) with index SNP rs1159974 map to fetal brain HGEs, with the locus centered on the promoter of the *HEY2* gene on chromosome 6q22 (Fig. 5*a*). The strongest cortical eQTL for *HEY2* of a SNP within an HGE is rs10457469 (FDR adjusted *P*-value = 7.09 × 10^−44^ derived from the adult brain PsychENCODE dataset (Wang et al. 2018), *r*^2^_rs10457469:rs1159974_ = 1), which regulates neural progenitor proliferation during neurogenesis (Sakamoto et al. 2003).

Next, we leveraged our recently generated dataset of chromatin accessibility (ca) QTLs in human cortical neural progenitors and their differentiated neuronal progeny (Liang et al. 2020) to further understand the influence of genetic variation on gene regulatory elements in the developing brain. Using this dataset, we identified a chromatin accessibility peak at the promoter of *HEY2* (chr6:125746611–125750660) that overlapped with multiple HGEs and had significantly higher accessibility in progenitors than in neurons (logFC = 0.484, FDR adjusted *P*-value = 5.98 × 10^−31^; Fig. 5*a*–*d*). A SNP associated with differences in chromatin accessibility (caSNP) within this peak (rs7764016) was in high LD (*r*^2^ = 0.823 calculated using the donors of the caQTL dataset; *r*^2^ = 0.988 calculated in 1000G phase 3 EUR dataset) with the index SNP associated with *pars opercularis* SA (rs1159974). The allele linked to decrease in SA and increased *HEY2* gene expression (T) was associated with higher chromatin accessibility of the promoter peak in progenitors (*P*-value = 3.99 × 10^−8^) but not in neurons (*P*-value = 0.68; Fig. 5*a*,*b*). To provide further support for these findings, we used an alternative method for inferring allelic effects on chromatin accessibility within heterozygous donors (allele specific chromatin accessibility) at rs7764016 and found that the T allele was associated with higher chromatin accessibility in both progenitors (*P*-value = 1.51 × 10^−10^) and neurons (*P*-value = 7.45 × 10^−8^; Fig. 5*c*). We controlled for the GWAS index SNP in the progenitor caQTL analysis which abolished the caQTL signal, demonstrating that these variants mark the same locus (co-localization; Fig. 5*a*). Overall, we suggest a causal variant (rs7764016) where the T allele is associated with increased chromatin accessibility in neural progenitors at an HGE near the promoter of *HEY2*, increased gene expression of *HEY2*, and decreased cortical SA of the *pars opercularis*. Conversely, the derived allele (G) of rs7764016 is associated with reduced *HEY2* expression and increased cortical SA for this region. The Human Genome Dating atlas estimates this derived allele to be 2993.7 (2660.5–3316.7, 95% confidence interval) generations old (Albers and McVean 2020). Assuming 25 years per generation, the estimated age of the derived allele is 74 (66–82) kya. These results indicate that genetically mediated alteration of the function of a regulatory element with specific activity in the developing human brain impacts adult inferior frontal cortical SA. The findings also suggest a specific gene and regulatory element involved in shaping inferior frontal gyrus cortical structure in humans, acting within a polygenic framework.

Likely due to the limited number of eGenes identified, no significant (FDR < 0.05) gene ontology terms with greater than 5 intersections with HGE regulated genes were identified. Nevertheless, this analysis points to specific developmentally interesting genes regulated by HGEs which have shaped both the overall SA of the cortex and specific regions. Plots of all genome-wide significant loci that overlapped with one or more of the evolutionary annotations considered in this study are provided in Supplementary Figure 11.

## Discussion

By integrating genomic annotations of primate evolutionary history with the largest available genome-wide association analysis of neuroanatomy in living populations (Grasby et al. 2020), we are able to map genetic variation shaping cortical SA across different time periods on the lineage that led to modern humans. We find evidence of polygenic selection influencing global SA over the past 2000–3000 years. Notably, the signals of polygenic selection for increased SA in parts of the inferior frontal gyrus highlight cortical regions known to be important for the production of spoken language. These results are interesting in light of a recent study that used paleoanthropology, speech biomechanics, ethnography, and historical linguistics to show that changes in human bite configuration and speech-sound inventories occurred after the Neolithic period, potentially due to advances in food-processing technologies (Blasi et al. 2019). Thus, it is plausible that the consequent increases in the diversity of sounds produced may have led to a subtle, but consistent, polygenic selection of alleles increasing cortical SA in brain regions with relevance for speech. If this hypothesis is confirmed, it would represent a novel example of gene-culture co-evolution on the human lineage (Laland et al. 2010).

Considering a deeper evolutionary timescale, our analyses also reveal that common variation found within human-gained enhancers that are active during fetal development has effects on cortical SA measured largely in adults. Of note, regions of the inferior frontal gyrus were again among the most significant cortical areas implicated by our analyses, suggesting that they have been subject to evolutionary processes at multiple distinct timepoints on the lineage that led to modern humans. These findings implicate neural progenitor proliferation and differentiation as processes critical to evolutionary expansion of cortical SA on the human lineage. Such a relationship is consistent with the radial unit hypothesis (Rakic 2009), which posits that cortical expansion is driven by an increase in the progenitor pool present during development. In addition, through the integration of multi-omic QTLs, brain structure GWAS, and evolutionarily relevant genomic annotations, we identify a regulatory element near the promoter of *HEY2* with activity specific to humans where sequence variation in that locus impacts the cortical structure of the inferior frontal gyrus. We note that the decreased expression of *HEY2* is associated with increased cortical SA. Work in mice links this gene to neural progenitor proliferation (Sakamoto et al. 2003). The effect of allelic regulation of HEY2 expression levels on progenitor proliferation and cortical areal size will depend on spatiotemporal patterns of HEY2 expression and interactions with other factors that are co-expressed with it in the different regions. We believe that this represents a novel approach to identify the functional impact of evolutionarily relevant regulatory elements on brain structure. Intriguingly, a rare single gene duplication of *HEY2* was identified in a child with cardiac and neurodevelopmental deficits, including disrupted speech development (Jordan et al. 2015). Although this case report requires further support from identification and characterization of additional mutation carriers, it is consistent with our association of *HEY2* promoter variants with changes in cortical SA of inferior frontal regions, as these brain areas are known to be hubs in distributed circuits involved in speech and language processing.

Our study should be carefully interpreted in light of some limitations. First, in this study, we were only able to assess a subset of genetic variation that is important for human cortical SA expansion and refinement through human evolution. Specifically, we assess alleles that are both common and polymorphic in current human populations, with a bias toward European ancestry. It is almost certain that derived alleles that are now fixed in modern human populations (and therefore not detectable in GWAS) also made substantial contributions to the shaping of cortical SA during hominid evolution. So far, relatively few of these variants are known (Sousa et al. 2017), but future studies, for example introducing fixed chimpanzee or Neanderthal alleles into human neural progenitor cells, will help to assess the impacts of this class of genetic variants (Ryu et al. 2018). Second, our study is limited to understanding selective pressures within defined historical windows from evolutionarily relevant genomic annotations (Fig. 1). Our SDS correlations suggest that polygenic selective forces impacted human cortical structure but are uninformative about the timepoint that polygenic selective forces first acted because SDS does not provide information concerning evolutionary periods preceding 3000 years ago. Third, subtle population stratification can influence the inferences of polygenic selection impacting a trait (Berg et al. 2018; Sohail et al. 2019). Prior to analyses of GWAS data, we implemented an ancestry regression procedure to correct for subtle population stratification (Bhatia et al. 2016). We show that this procedure reduces the impact of population stratification, as evaluated by two independent methods (ancestry PC correlations and LDSC intercept). However, LDSC intercepts were not uniformly at 1 (indicative of no population stratification) suggesting that some residual population stratification remains. Allele frequency differences across populations may not be independent of selective pressures, so our procedure may also over-correct leading to diminished evidence of selective effects. Even using our conservative ancestry regression approach, robust signals of polygenic selection were detected for cortical SA, giving us confidence in the results. Nevertheless, replication of our findings in future genetic association studies of brain structure in sufficiently large family-based populations that are less susceptible to impacts of population stratification (Spielman and Ewens 1996; Hemani et al. 2013) would allow further verification of the results presented here. Finally, future studies focusing on understanding genetic influences on behavioral and cognitive traits (language, motor skills) (Deriziotis and Fisher 2017) combined with GWAS of their neurobiological substrates (like this one) may provide a more complete picture of how shifts in genetic variation across time might yield changes in brain structure and behavior.

These findings provide new insights into a number of long-standing debates about the genetic basis for brain size and cortical SA expansion in modern humans. First, consistent with the idea that noncoding genetic variation is a large driver of human brain evolution (King and Wilson 1975), we note that genomic annotations of evolutionary history in which cortical SA heritability enrichment was observed are not derived from protein-coding variations. Instead, these come largely from noncoding intergenic or specifically regulatory sequences. Second, our work refutes prior claims that an evolutionary change in just one gene (or perhaps a small handful of genes) can fully account for the distinctive nature of the modern human brain. For example, it was previously proposed that a single genetic variant of strong effect was sufficient to cause the expansion of human brains and cognitive abilities around 50 kya (Klein 2002). Here, we not only show that variation in multiple human-gained enhancers influences cortical SA in aggregate, but also find evidence of much more recent polygenic selection acting on these traits. We clarified molecular mechanisms for one of the genes contributing to the overall polygenic signal, *HEY2*, by integration of multi-omic datasets. Thus, multiple alleles each of small effect have contributed to the shaping of modern human cortical SA across different evolutionary timescales, even within the last 2000–3000 years, supporting the importance of gene-culture co-evolution in explaining our biology. In sum, selective pressures over the last 30 million years of human evolution appear to have shaped different aspects of modern human brain structure, from ancient effects on broad growth patterns through to much more recent influences on a number of cortical regions, including those linked to our capacity for spoken language.

## Notes

We thank Leo Zsembik and Shana Hall for initial work on polygenic selection analyses. We also thank Dr Philipp Gunz for many helpful discussions during the development of Figure 1. S.E.F. is a member of the Center for Academic Research and Training in Anthropogeny (CARTA). *Conflict of Interest*: D.P.H. is a full-time employee of Genentech, Inc.

## Funding

Foundation of Hope (to J.L.S.); the Brain Research Foundation (to J.L.S.); the National Institutes of Health (R01 MH118349, R00 MH102357, U54 EB020403 to J.L.S.); the National Science Foundation (ACI-16449916 to J.L.S.); the Max Planck Society (to S.E.F.); APP1173025 (to K.L.G.).

## Author Contributions

S.E.F. and J.L.S. originated the project and oversaw the work. A.K.T., S.E.F., and J.L.S. drafted the manuscript. A.K.T. performed partitioned heritability analyses. A.K.T., D.L., and J.L.S. identified specific genes and regulatory elements impacted by HGE. J.L.S. implemented ancestry regression. S.L., S.M.B., and J.L.S. implemented recent polygenic selection analyses. E.A.K., B.E.S., and L.K.D. provided data for performing ancestry regression and independently implemented analyses. K.L.G., N.J., J.P., L.C.C., J.B., D.P.H., P.A.L., P.M.T., S.E.M., and J.L.S. provided the SA GWAS summary statistics. All authors edited the manuscript.

## Supplementary Material

SUPPLEMENTAL_INFORMATION_ENIGMA_Evol_bhaa327

FigS11_bhaa327

TableS2_bhaa327
