# Supplementary material for: The Evolutionary History of Common Genetic Variants Influencing Human Cortical Surface Area
Source: Cereb Cortex. 2020 Dec 9;31(4):1873–87. doi: 10.1093/cercor/bhaa327 (PMC7945014; doi:10.1093/cercor/bhaa327)
Supplement: SUPPLEMENTAL_INFORMATION_ENIGMA_Evol_bhaa327 [file supplemental_information_enigma_evol_bhaa327.pdf]

SUPPLEMENTAL INFORMATION

Table S1: Characteristics of human-evolution focused genome annotations in 1000 Genomes Phase 3 (EUR)

Name	# Regions	Total Size (kb)	# 1KG† SNPs	Avg. MAF†	Reference
Human Accelerated Regions	2,737	702	1,874	0.213	(Capra et al. 2013)
Human Gained Enhancers - 7PCW	7,742	22,195	57,059	0.205	(Reilly et al. 2015)
Human Gained Enhancers - 8.5PCW	5,101	12,277	29,985	0.203	(Reilly et al. 2015)
Human Gained Enhancers - 12PCW (Frontal)	3,110	8,079	20,741	0.203	(Reilly et al. 2015)
Human Gained Enhancers - 12PCW (Occipital)	4,994	12,297	29,344	0.203	(Reilly et al. 2015)
Human Gained Enhancers - Adult human vs macaque (prefrontal cortex)	2852	12,692	32,009	0.206	(Vermunt et al. 2016)
Human Gained Promoters - Adult human vs macaque (prefrontal cortex)	358	2,048	5,111	0.213	(Vermunt et al. 2016)

Human Gained Enhancers - Adult human vs chimpanzee (prefrontal cortex)	435	1,826	4,749	0.212	(Vermunt et al. 2016)
Human Gained Promoters - Adult human vs chimpanzee (prefrontal cortex)	28	129	329	0.242	(Vermunt et al. 2016)
Neanderthal Introgression SNPs	5,851	722,954	24,331	0.115	(Simonti et al. 2016)
Neanderthal Lineage Depleted Regions	6	84,440	175,377	0.208	(Vernot et al. 2016)
Selective Sweeps	314	19,064	23,744	0.196	(Peyrégne et al. 2017)

†Based on 1000 Genomes, phase 3 SNPs that were included in the ENIGMA3 meta-analysis

Table S2. eGenes impacted by loci within HGEs that are also associated with Regional or Global SA. See separate excel file.

Table S3. Irritable bowel disease heritability is not enriched in any of the evolution-focused annotations considered in this study. P-values are FDR-corrected for the number of annotations tested.

**Annotation**	**Prop. *h2***	**Prop. *h2* SE**	**Enrichment**	**Enrichment SE**	**FDR-corrected Enrichment *p***
HGE 12pcw - Frontal	-0.002	0.012	-0.715	3.840	0.833
HGE 12pcw - Occipital	0.025	0.017	5.602	3.945	0.629
HGE 7pcw	0.059	0.019	7.014	2.194	0.072
HGE 8.5pcw	0.024	0.017	5.463	3.797	0.629
Chimp PFC enhancers	0.001	0.004	1.781	5.344	0.884
Chimp PFC promoters	0.000	0.001	4.824	24.631	0.884
Macaque PFC enhancers	-0.007	0.012	-1.416	2.426	0.629
Macaque PFC promoters	-0.001	0.005	-1.668	6.811	0.833
HAR	-0.003	0.005	-11.108	18.704	0.775
Neanderthal lineage depleted regions	0.018	0.006	0.645	0.222	0.629
Neanderthal SNPs	0.002	0.004	0.309	0.664	0.629
Selective sweeps	0.001	0.003	0.288	0.788	0.629

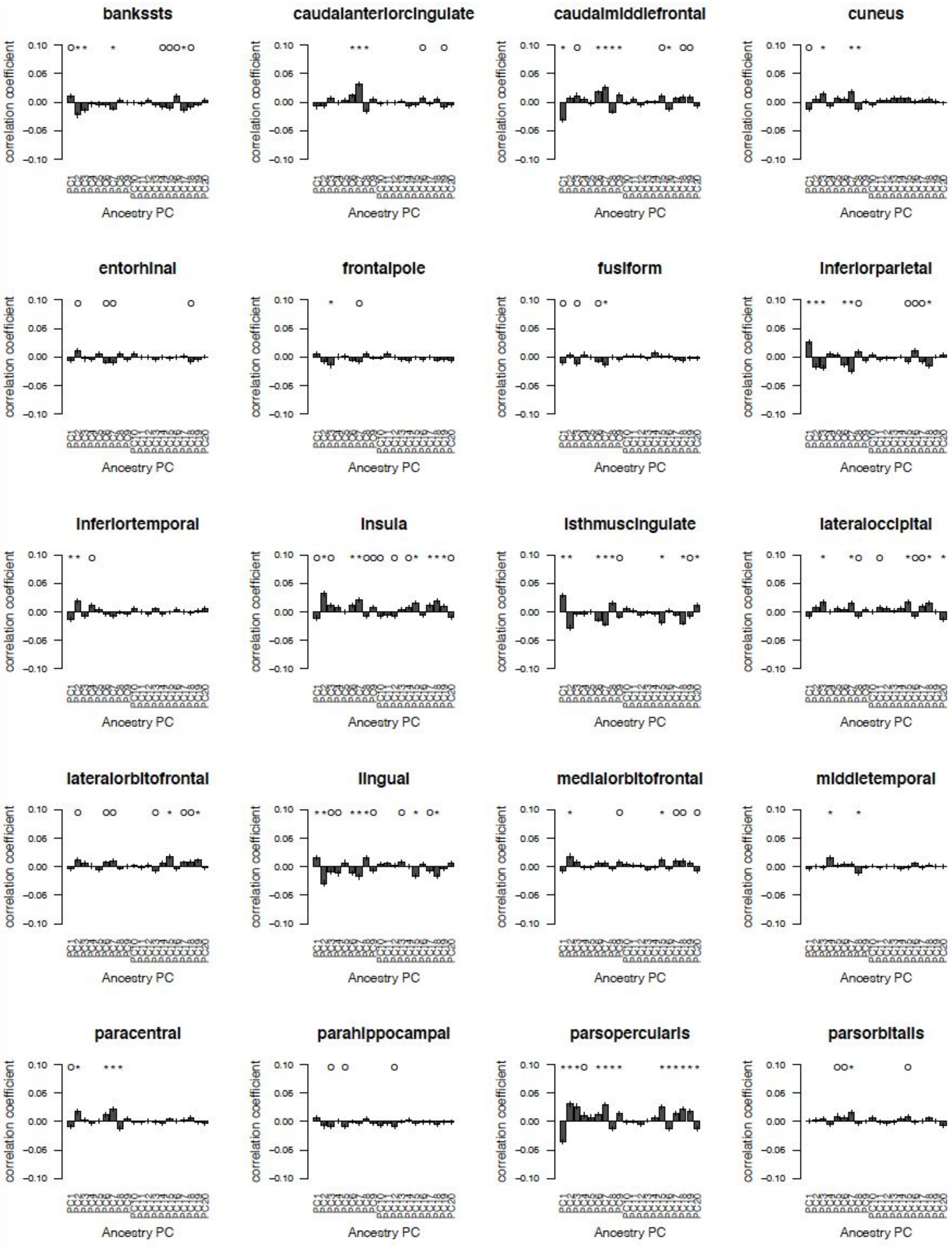

bankssts
caudalanteriorcingulate
caudalmiddlefrontal
cuneus
entorhinal
frontalpole
fusiform
inferiorparietal
inferiortemporal
insula
isthmuscingulate
lateraloccipital
lateralorbitofrontal
lingual
medialorbitofrontal
middletemporal
paracentral
parahippocampal
parsopercularis
parsorbitalis
correlation coefficient
0.10
0.05
0.00
-0.05
-0.10
Ancestry PC

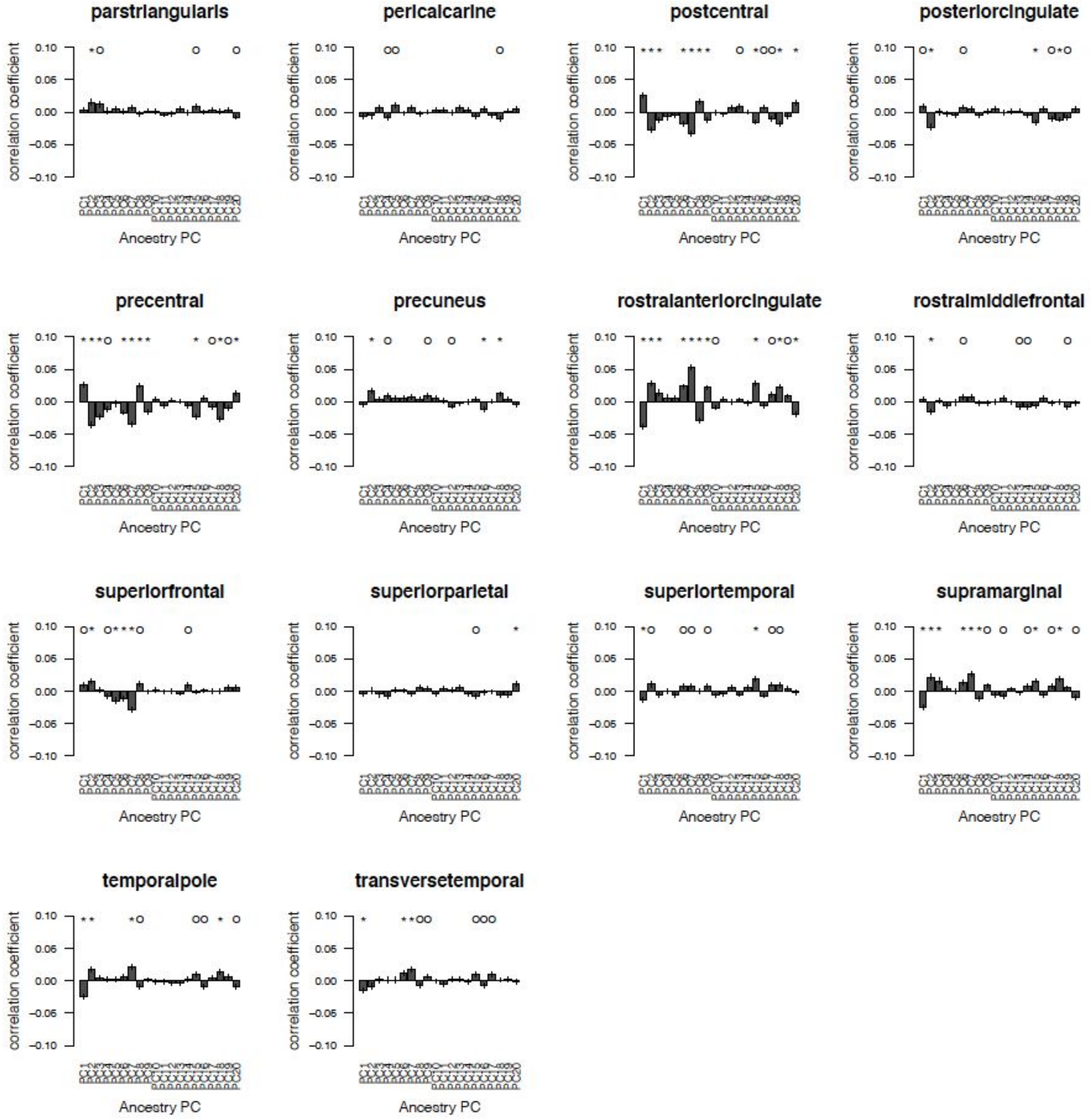

Figure S1. Detecting subtle ancestry regression in cortical SA regional GWASs. Error bars are standard errors.

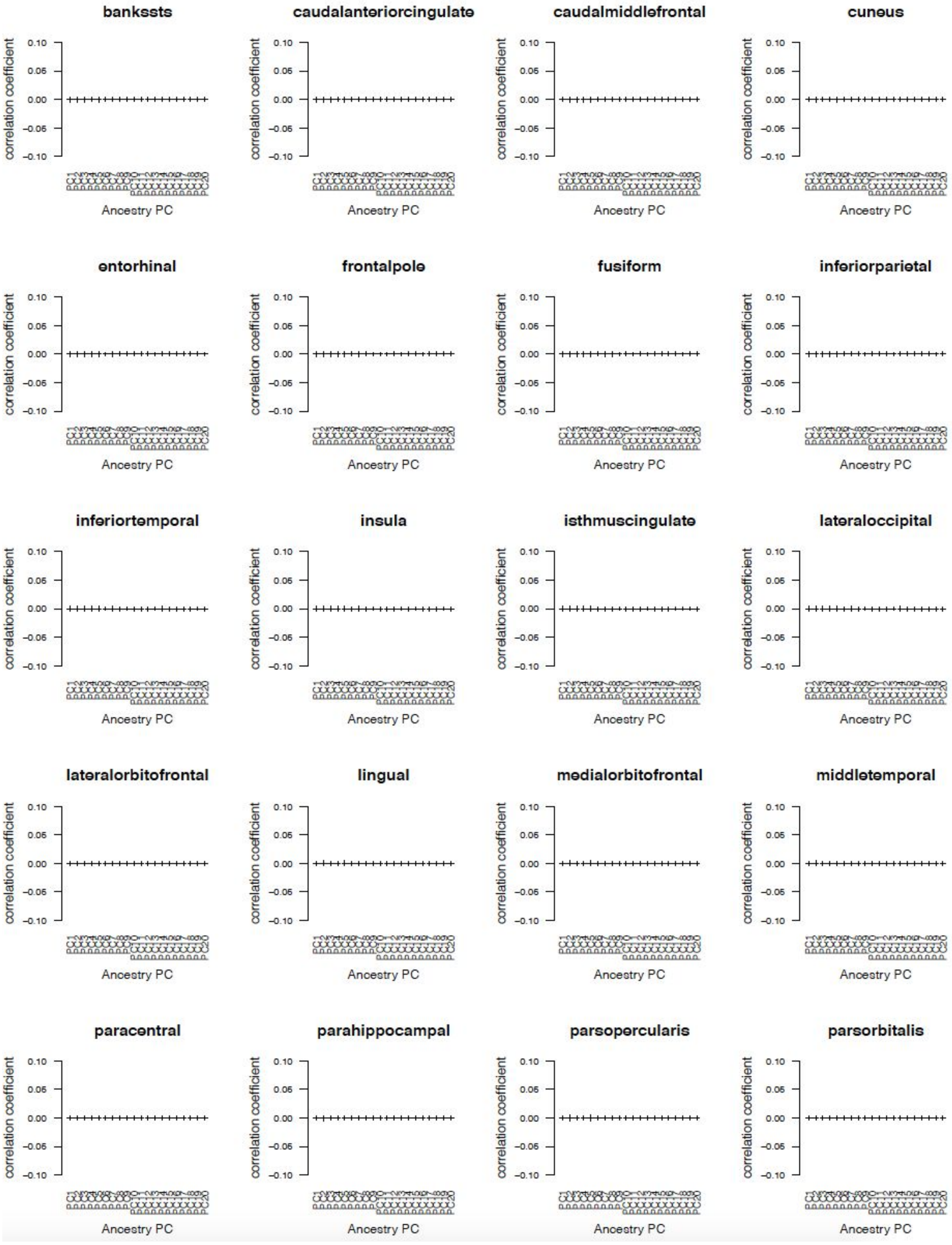
bankssts
caudalanteriorcingulate
caudalmiddlefrontal
cuneus
entorhinal
frontalpole
fusiform
inferiorparietal
inferiortemporal
insula
isthmuscingulate
lateraloccipital
lateralorbitofrontal
lingual
medialorbitofrontal
middletemporal
paracentral
parahippocampal
parsopercularis
parsorbitalis
correlation coefficient
0.10
0.05
0.00
−0.05
−0.10
Ancestry PC

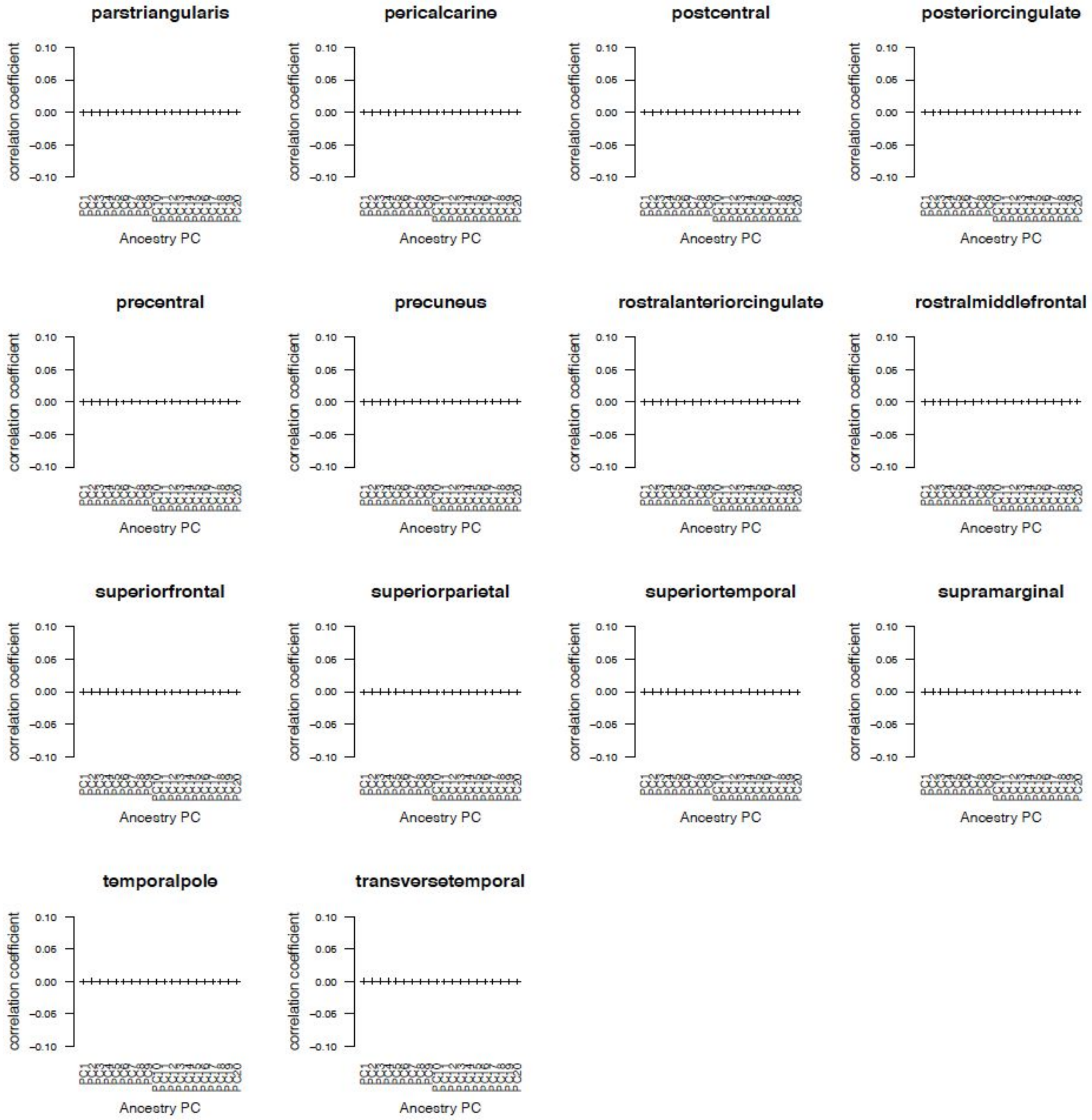

Figure S2. Ancestry regressed cortical SA regional GWASs show diminished effects of subtle population stratification. Error bars are standard errors.

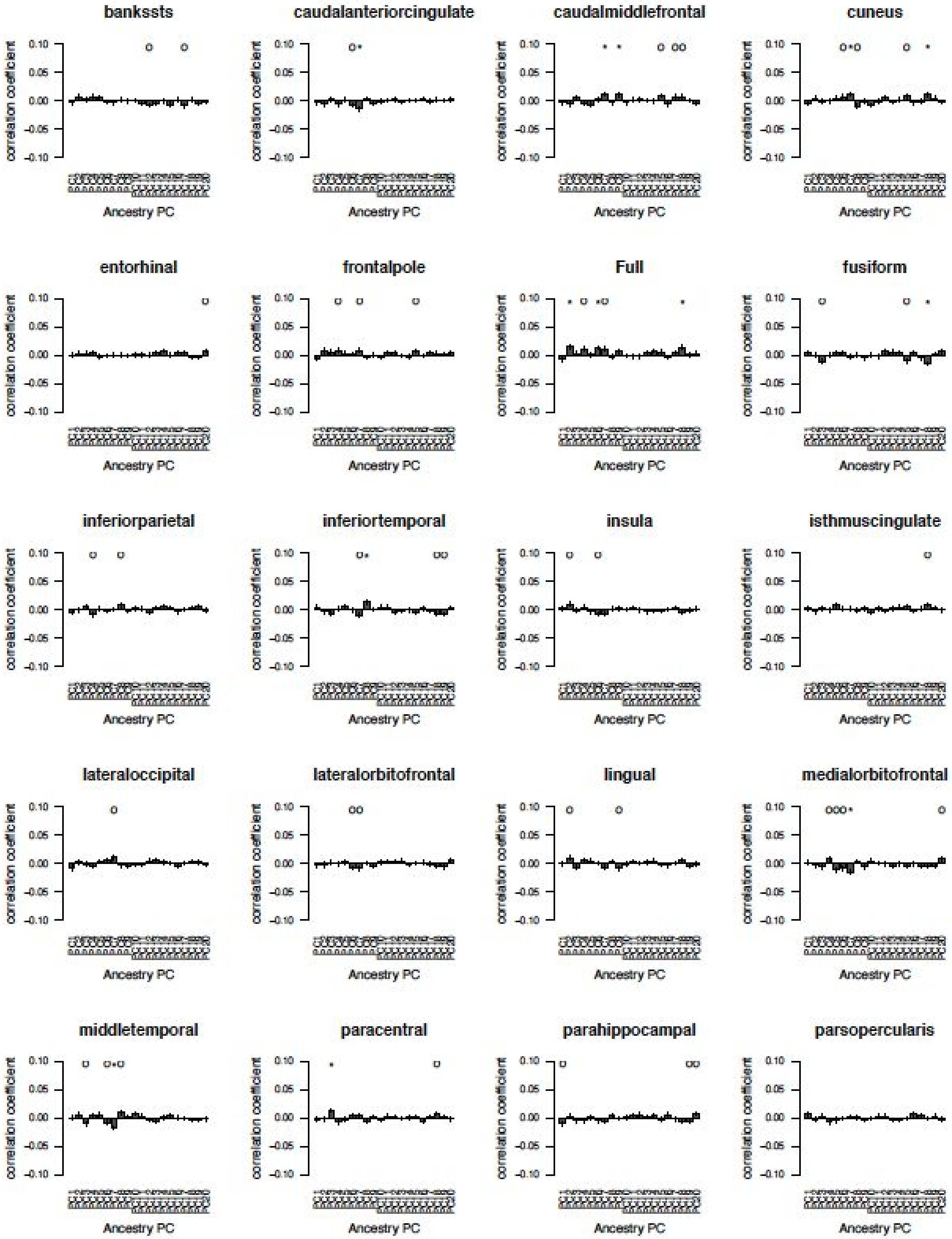
bankssts
caudalanteriorcingulate
caudalmiddlefrontal
cuneus
entorhinal
frontalpole
Full
fusiform
inferiorparietal
inferiortemporal
insula
isthmuscingulate
lateraloccipital
lateralorbitofrontal
lingual
medialorbitofrontal
middletemporal
paracentral
parahippocampal
parsopercularis
correlation coefficient
0.10
0.05
0.00
−0.05
−0.10
Ancestry PC

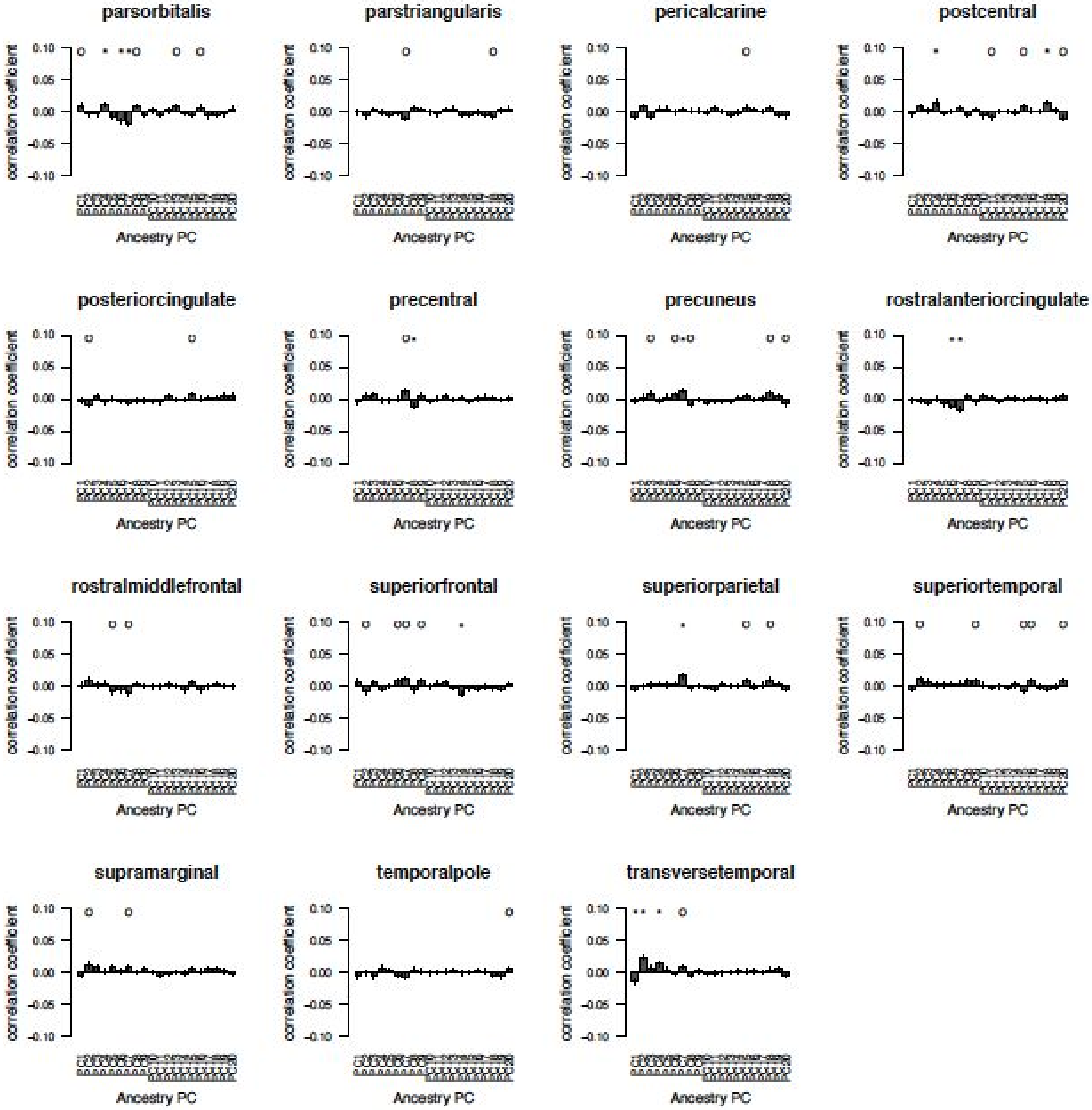

Figure S3. Detecting subtle ancestry regression in cortical thickness regional GWASs. Error bars are standard errors.

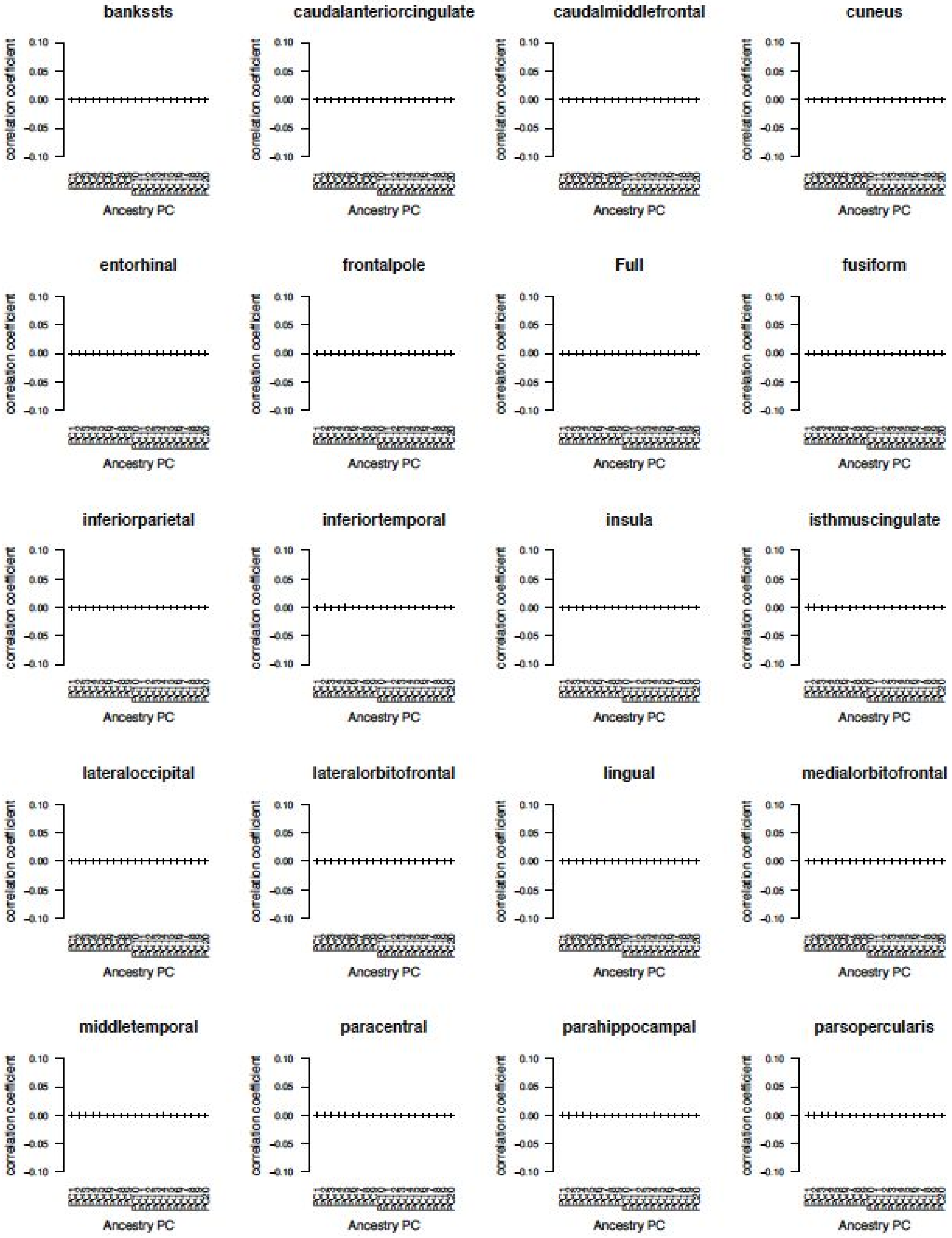

bankssts
caudalanteriorcingulate
caudalmiddlefrontal
cuneus
entorhinal
frontalpole
Full
fusiform
inferiorparietal
inferiortemporal
insula
isthmuscingulate
lateraloccipital
lateralorbitofrontal
lingual
medialorbitofrontal
middletemporal
paracentral
parahippocampal
parsopercularis
correlation coefficient
0.10
0.05
0.00
-0.05
-0.10
Ancestry PC

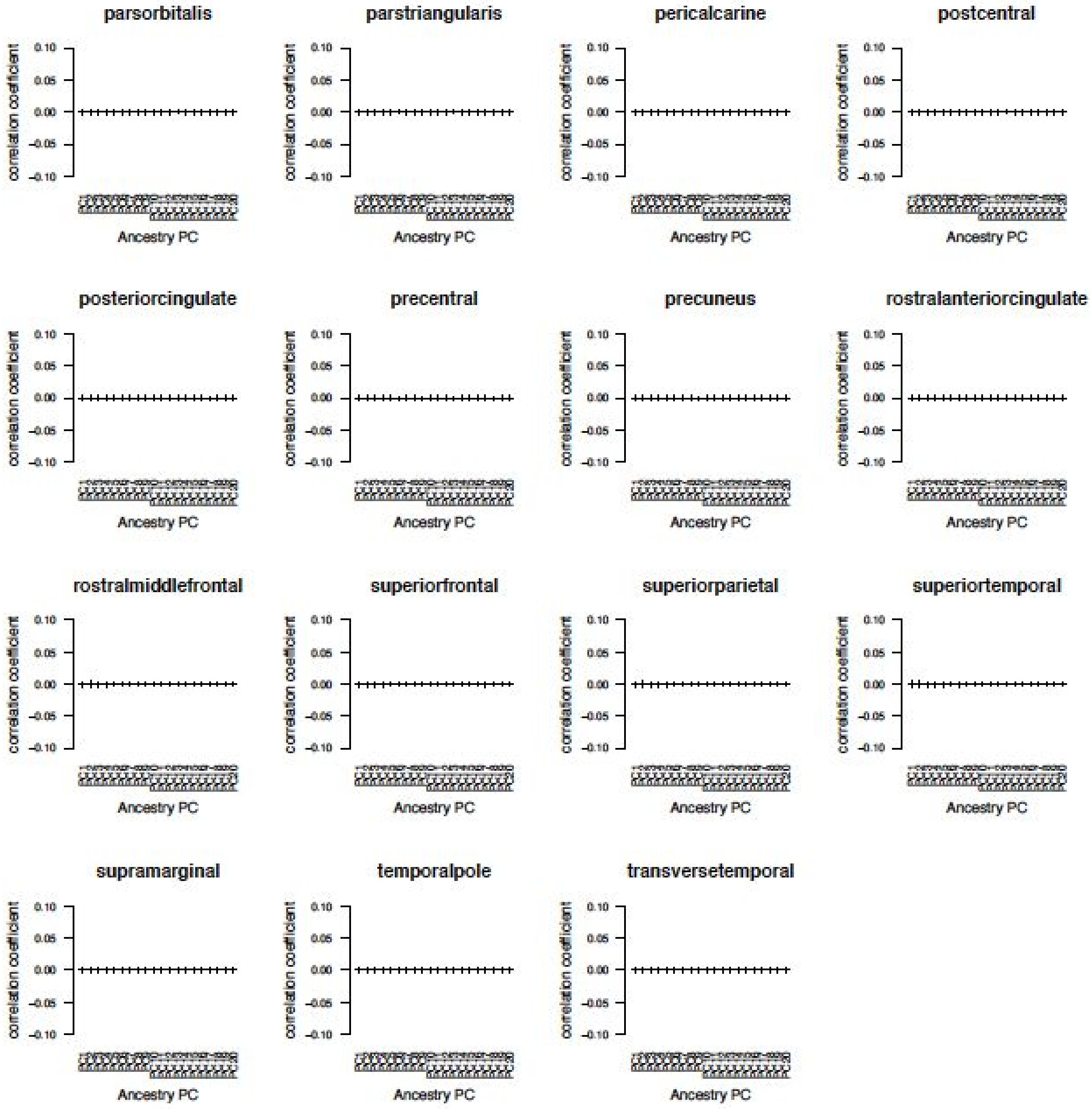

Figure S4. Ancestry regressed cortical thickness regional GWASs show diminished effects of subtle population stratification. Error bars are standard errors.

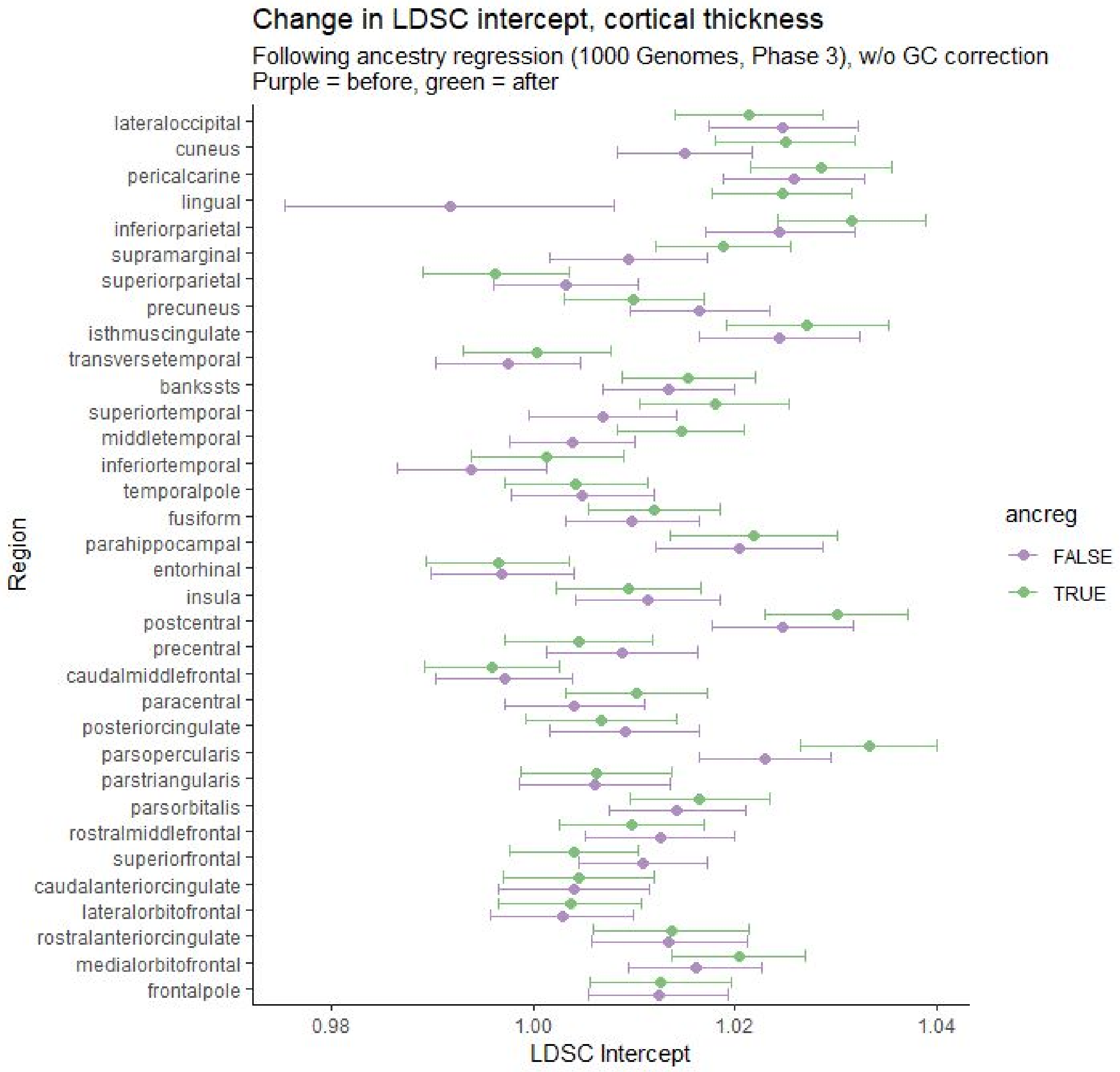

Figure S5. LD-score regression (LDSC) intercepts before and after ancestry regression for cortical thickness phenotypes.

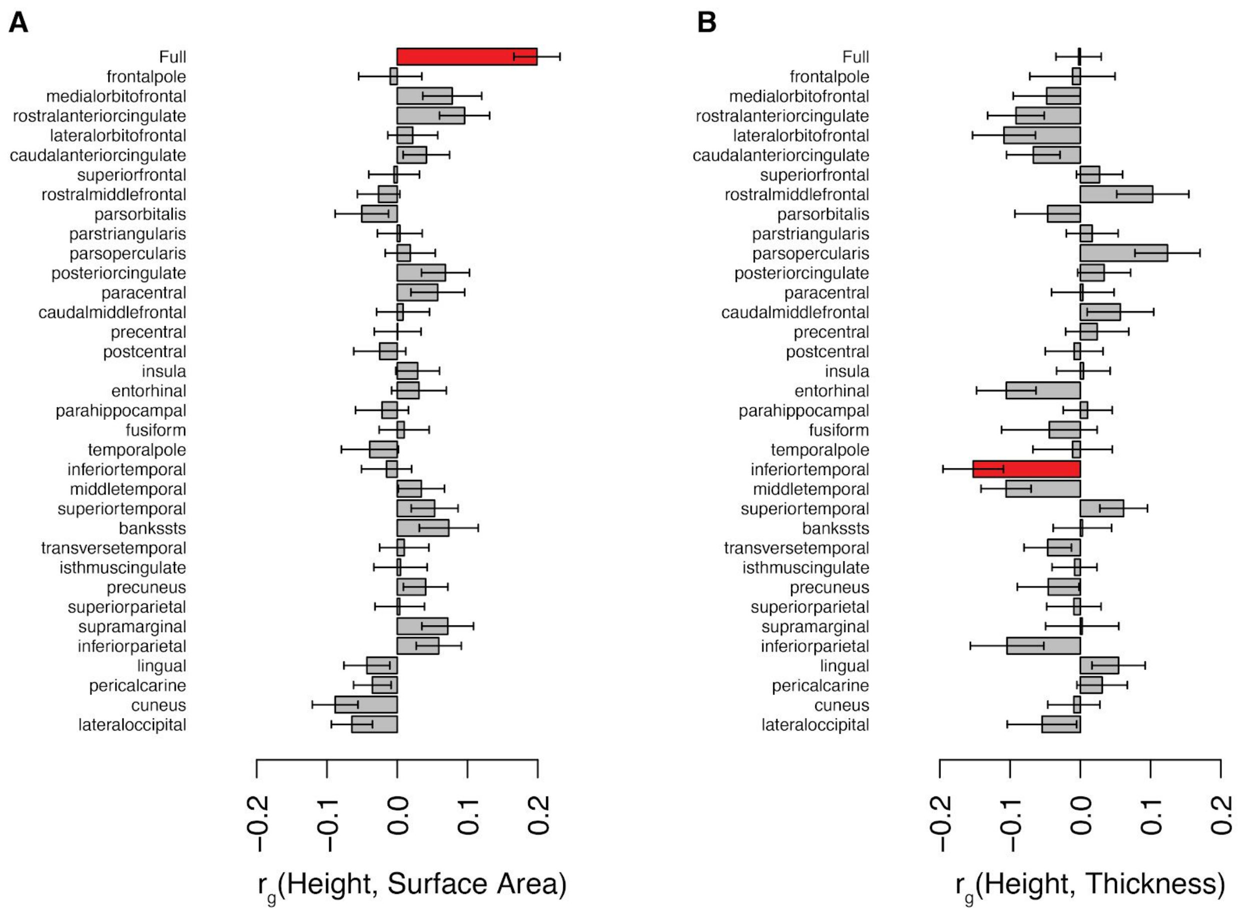

Figure S6. Genetic correlations between cortical surface area **(a)** and thickness **(b)** with height (Wood et al. 2014). Red bars indicate a significant correlation after FDR correction for multiple comparisons within the 35 traits of either surface area or thickness.

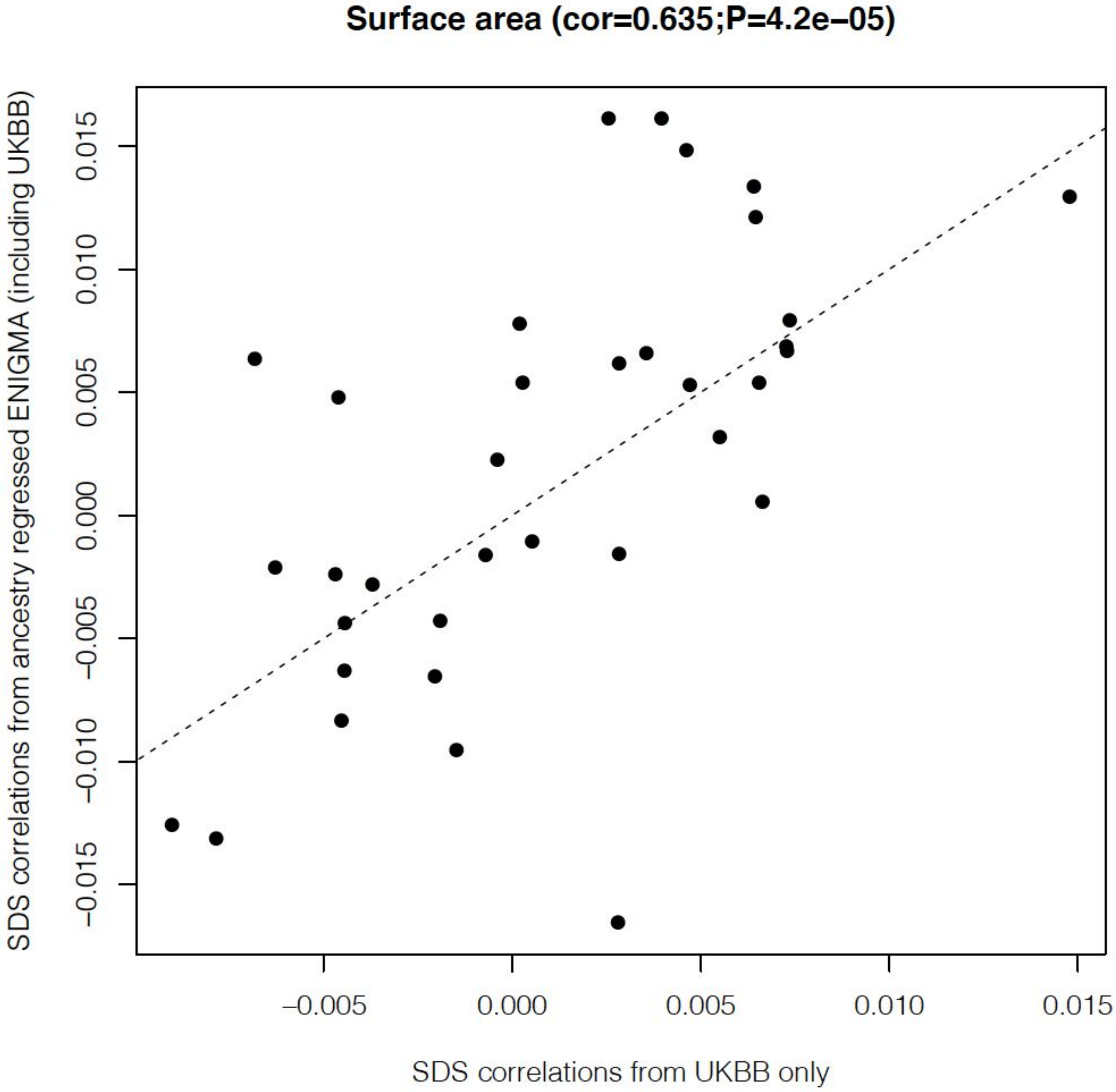

Figure S7. SDS correlations to cortical surface area in a population less susceptible to subtle population stratification (UKBB EUR) and the ancestry regressed ENIGMA data. The y=x line is shown.

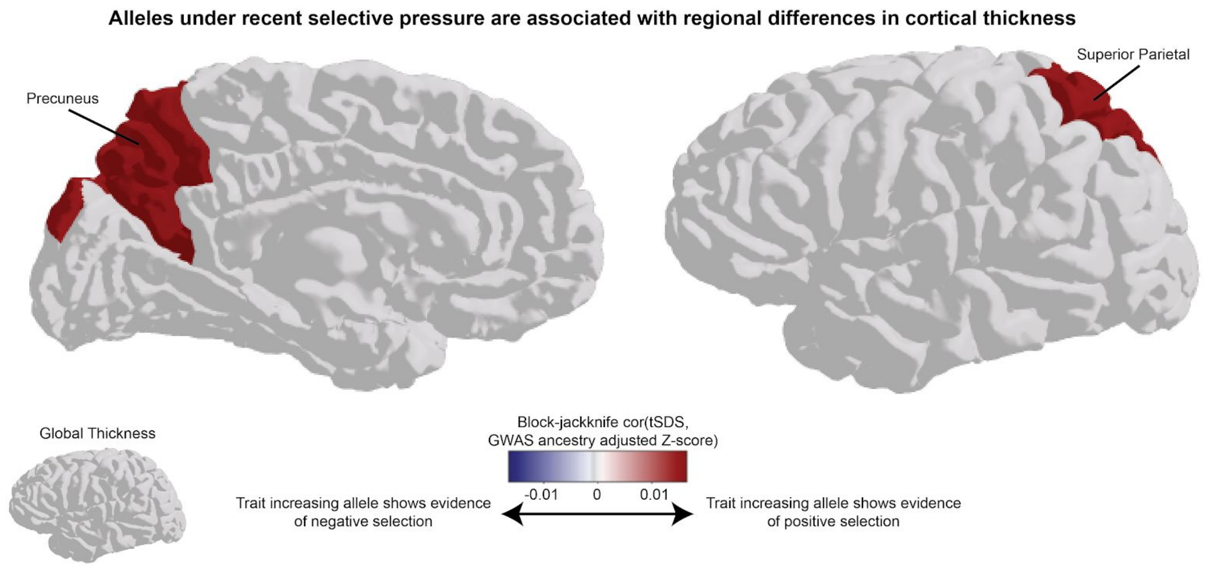

Figure S8. Evidence for haplotypes under recent polygenic selection (~2000-3000 years) impacting cortical thickness. (a) A block-jackknife correlation of ancestry regressed effect sizes from GWAS (Z-scores) with scores of recent selection (tSDS) demonstrates evidence for polygenic alleles under selective pressure also influencing both global and regional thickness (colored regions indicate FDR < 0.05). Colder colors indicate that the trait increasing alleles (associated with increased thickness) are generally associated with negative selection (decreasing allele frequencies in the population), whereas warmer colors indicate that trait increasing alleles are associated with positive selection.

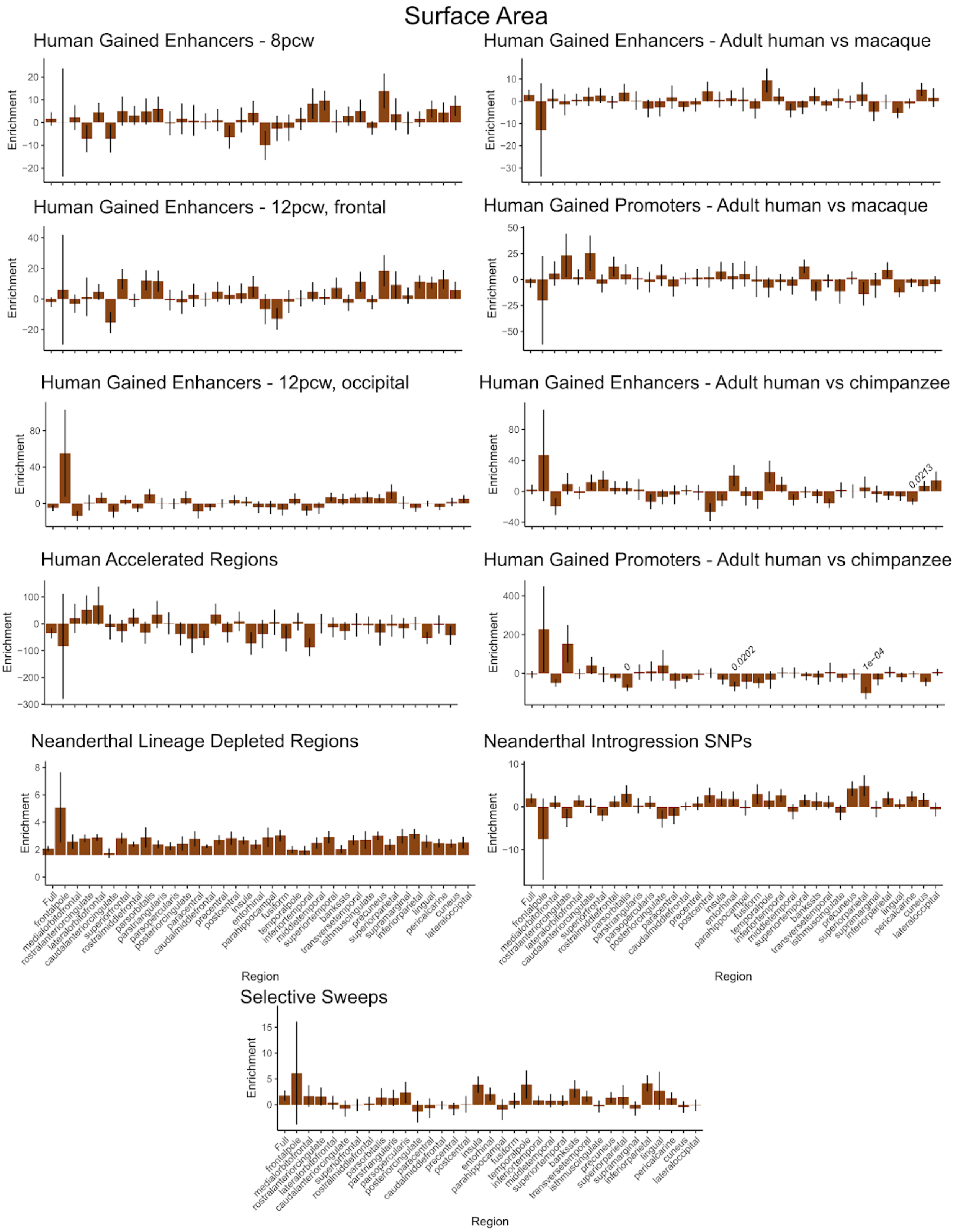

Surface Area
Human Gained Enhancers - 8pcw
Human Gained Enhancers - Adult human vs macaque
Human Gained Enhancers - 12pcw, frontal
Human Gained Promoters - Adult human vs macaque
Human Gained Enhancers - 12pcw, occipital
Human Gained Enhancers - Adult human vs chimpanzee
0.0213
Human Accelerated Regions
Human Gained Promoters - Adult human vs chimpanzee
0
0.0202
1e−04
Neanderthal Lineage Depleted Regions
Neanderthal Introgression SNPs
Selective Sweeps
Enrichment
Region
Full
frontalpole
medialorbitofrontal
rostralanteriorcingulate
lateralorbitofrontal
caudalanteriorcingulate
superiorfrontal
rostralmiddlefrontal
parsorbitalis
parstriangularis
parsopercularis
posteriorcingulate
paracentral
caudalmiddlefrontal
precentral
postcentral
insula
entorhinal
parahippocampal
fusiform
temporalpole
inferiortemporal
middletemporal
superiortemporal
bankssts
transversetemporal
isthmuscingulate
precuneus
superiorparietal
supramarginal
inferiorparietal
lingual
pericalcarine
cuneus
lateraloccipital

Figure S9. Cortical surface area enrichment scores for Human Accelerated Regions (HARs), fetal and adult HGEs, selective sweeps, Neanderthal introgressed regions, and Neanderthal depleted regions. Error bars represent standard errors, numbers above bars indicate FDR corrected p-values less than 0.05.

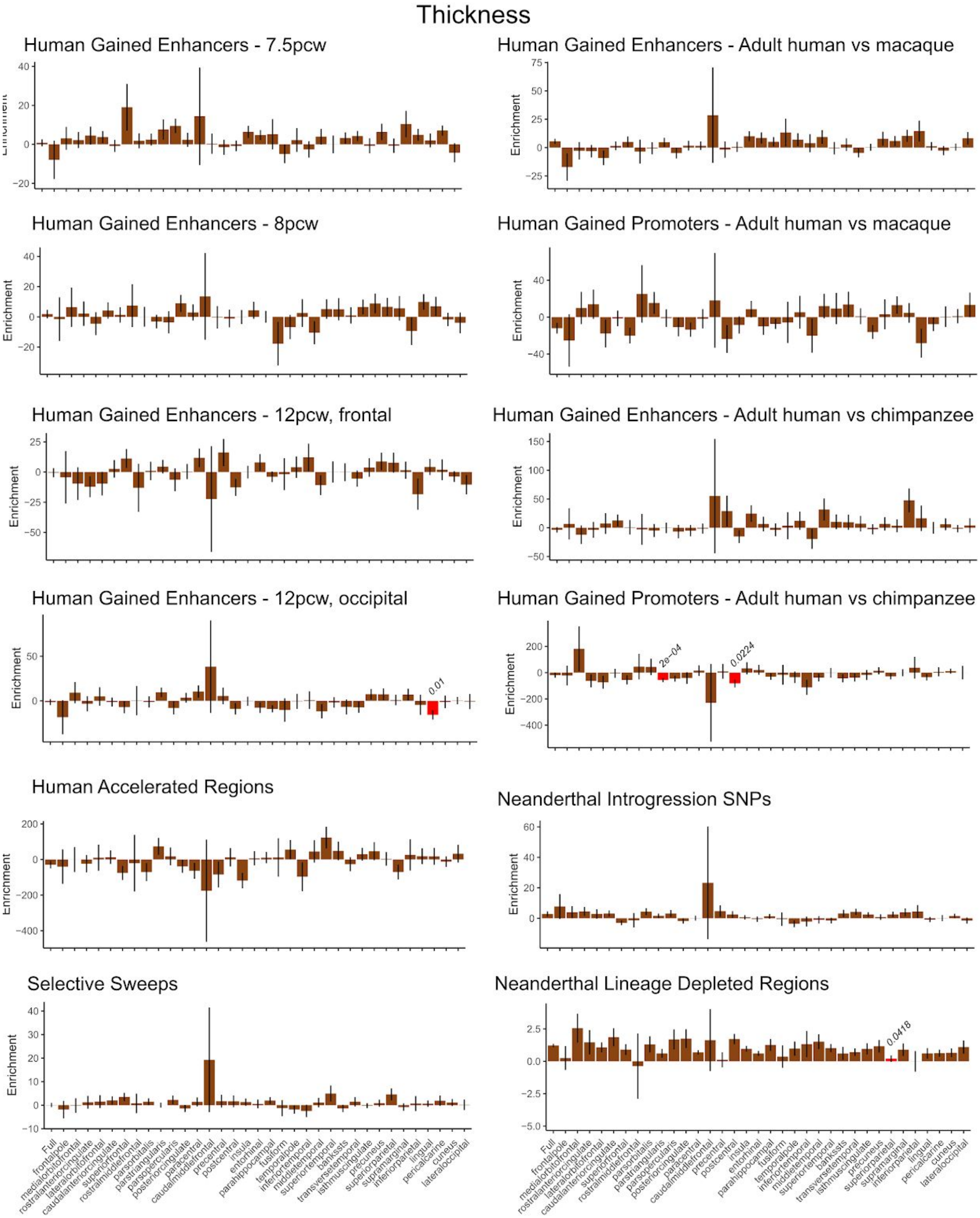
Thickness
Human Gained Enhancers - 7.5pcw
Human Gained Enhancers - Adult human vs macaque
Human Gained Enhancers - 8pcw
Human Gained Promoters - Adult human vs macaque
Human Gained Enhancers - 12pcw, frontal
Human Gained Enhancers - Adult human vs chimpanzee
Human Gained Enhancers - 12pcw, occipital
0.01
Human Gained Promoters - Adult human vs chimpanzee
2e-04
0.0224
Human Accelerated Regions
Neanderthal Introgression SNPs
Selective Sweeps
Neanderthal Lineage Depleted Regions
0.0418
Enrichment
Full
frontalpole
medialorbitofrontal
rostralanteriorcingulate
lateralorbitofrontal
caudalanteriorcingulate
superiorfrontal
rostralmiddlefrontal
parsorbitalis
parstriangularis
parsopercularis
posteriorcingulate
paracentral
caudalmiddlefrontal
precentral
postcentral
insula
entorhinal
parahippocampal
fusiform
temporalpole
inferiortemporal
middletemporal
superiortemporal
bankssts
transversetemporal
isthmuscingulate
precuneus
superiorparietal
supramarginal
inferiorparietal
lingual
pericalcarine
cuneus
lateraloccipital

Figure S10. Cortical thickness enrichment scores for Human Accelerated Regions (HARs), fetal and adult HGEs, selective sweeps, Neanderthal introgressed regions, and Neanderthal depleted regions. Error bars represent standard errors, numbers above bars indicate FDR corrected p-values less than 0.05. Negative significant enrichment scores are labeled with the FDR corrected p-values, but are not discussed due to difficulty in interpretation.

Figure S11. GViz plots for 60 GWAS loci are available as a separate PDF.

References

Capra JA, Erwin GD, McKinsey G, Rubenstein JLR, Pollard KS. 2013. Many human accelerated regions are developmental enhancers. Philos Trans R Soc Lond B Biol Sci. 368:20130025.

Peyrégne S, Boyle MJ, Dannemann M, Prüfer K. 2017. Detecting ancient positive selection in humans using extended lineage sorting. Genome Res. 27:1563–1572.

Reilly SK, Yin J, Ayoub AE, Emera D, Leng J, Cotney J, Sarro R, Rakic P, Noonan JP. 2015. Evolutionary genomics. Evolutionary changes in promoter and enhancer activity during human corticogenesis. Science. 347:1155–1159.

Simonti CN, Vernot B, Bastarache L, Bottinger E, Carrell DS, Chisholm RL, Crosslin DR, Hebbring SJ, Jarvik GP, Kullo IJ, Li R, Pathak J, Ritchie MD, Roden DM, Verma SS, Tromp G, Prato JD, Bush WS, Akey JM, Denny JC, Capra JA. 2016. The phenotypic legacy of admixture between modern humans and Neandertals. Science. 351:737–741.

Vermunt MW, Tan SC, Castelijns B, Geeven G, Reinink P, de Bruijn E, Kondova I, Persengiev S, Netherlands Brain Bank, Bontrop R, Cuppen E, de Laat W, Creyghton MP. 2016. Epigenomic annotation of gene regulatory alterations during evolution of the primate brain. Nat Neurosci. 19:494–503.

Vernot B, Tucci S, Kelso J, Schraiber JG, Wolf AB, Gittelman RM, Dannemann M, Grote S, McCoy RC, Norton H, Scheinfeldt LB, Merriwether DA, Koki G, Friedlaender JS, Wakefield J, Pääbo S, Akey JM. 2016. Excavating Neandertal and Denisovan DNA from the genomes of Melanesian individuals. Science. 352:235–239.

Wood AR, Esko T, Yang J, Vedantam S, Pers TH, Gustafsson S, Chu AY, Estrada K, Luan J 'an, Kutalik Z, Amin N, Buchkovich ML, Croteau-Chonka DC, Day FR, Duan Y, Fall T, Fehrmann R, Ferreira T, Jackson AU, Karjalainen J, Lo KS, Locke AE, Mägi R, Mihailov E, Porcu E, Randall JC, Scherag A, Vinkhuyzen AAE, Westra H-J, Winkler TW, Workalemahu T, Zhao JH, Absher D, Albrecht E, Anderson D, Baron J, Beekman M, Demirkan A, Ehret GB, Feenstra B, Feitosa MF, Fischer K, Fraser RM, Goel A, Gong J, Justice AE, Kanoni S, Kleber ME, Kristiansson K, Lim U, Lotay V, Lui JC, Mangino M, Mateo Leach I, Medina-Gomez C, Nalls MA, Nyholt DR, Palmer CD, Pasko D, Pechlivanis S, Prokopenko I, Ried JS, Ripke S, Shungin D, Stancáková A, Strawbridge RJ, Sung YJ,

Tanaka T, Teumer A, Trompet S, van der Laan SW, van Setten J, Van Vliet-Ostaptchouk JV, Wang Z, Yengo L, Zhang W, Afzal U, Arnlöv J, Arscott GM, Bandinelli S, Barrett A, Bellis C, Bennett AJ, Berne C, Blüher M, Bolton JL, Böttcher Y, Boyd HA, Bruinenberg M, Buckley BM, Buyske S, Caspersen IH, Chines PS, Clarke R, Claudi-Boehm S, Cooper M, Daw EW, De Jong PA, Deelen J, Delgado G, Denny JC, Dhonukshe-Rutten R, Dimitriou M, Doney ASF, Dörr M, Eklund N, Eury E, Folkersen L, Garcia ME, Geller F, Giedraitis V, Go AS, Grallert H, Grammer TB, Gräßler J, Grönberg H, de Groot LCPGM, Groves CJ, Haessler J, Hall P, Haller T, Hallmans G, Hannemann A, Hartman CA, Hassinen M, Hayward C, Heard-Costa NL, Helmer Q, Hemani G, Henders AK, Hillege HL, Hlatky MA, Hoffmann W, Hoffmann P, Holmen O, Houwing-Duistermaat JJ, Illig T, Isaacs A, James AL, Jeff J, Johansen B, Johansson Å, Jolley J, Juliusdottir T, Junttila J, Kho AN, Kinnunen L, Klopp N, Kocher T, Kratzer W, Lichtner P, Lind L, Lindström J, Lobbens S, Lorentzon M, Lu Y, Lyssenko V, Magnusson PKE, Mahajan A, Maillard M, McArdle WL, McKenzie CA, McLachlan S, McLaren PJ, Menni C, Merger S, Milani L, Moayyeri A, Monda KL, Morken MA, Müller G, Müller-Nurasyid M, Musk AW, Narisu N, Nauck M, Nolte IM, Nöthen MM, Oozageer L, Pilz S, Rayner NW, Renstrom F, Robertson NR, Rose LM, Roussel R, Sanna S, Scharnagl H, Scholtens S, Schumacher FR, Schunkert H, Scott RA, Sehmi J, Seufferlein T, Shi J, Silventoinen K, Smit JH, Smith AV, Smolonska J, Stanton AV, Stirrups K, Stott DJ, Stringham HM, Sundström J, Swertz MA, Syvänen A-C, Tayo BO, Thorleifsson G, Tyrer JP, van Dijk S, van Schoor NM, van der Velde N, van Heemst D, van Oort FVA, Vermeulen SH, Verweij N, Vonk JM, Waite LL, Waldenberger M, Wennauer R, Wilkens LR, Willenborg C, Wilsgaard T, Wojczynski MK, Wong A, Wright AF, Zhang Q, Arveiler D, Bakker SJL, Beilby J, Bergman RN, Bergmann S, Biffar R, Blangero J, Boomsma DI, Bornstein SR, Bovet P, Brambilla P, Brown MJ, Campbell H, Caulfield MJ, Chakravarti A, Collins R, Collins FS, Crawford DC, Cupples LA, Danesh J, de Faire U, den Ruijter HM, Erbel R, Erdmann J, Eriksson JG, Farrall M, Ferrannini E, Ferrières J, Ford I, Forouhi NG, Forrester T, Gansevoort RT, Gejman PV, Gieger C, Golay A, Gottesman O, Gudnason V, Gyllensten U, Haas DW, Hall AS, Harris TB, Hattersley AT, Heath AC, Hengstenberg C, Hicks AA, Hindorff LA, Hingorani AD, Hofman A, Hovingh GK, Humphries SE, Hunt SC, Hypponen E, Jacobs KB, Jarvelin M-R, Jousilahti P, Jula AM, Kaprio J, Kastelein JJP, Kayser M, Kee F, Keinanen-Kiukaanniemi SM, Kiemeney LA, Kooner JS, Kooperberg C, Koskinen S, Kovacs P, Kraja AT, Kumari M, Kuusisto J, Lakka TA, Langenberg C, Le Marchand L, Lehtimäki T, Lupoli S, Madden PAF, Männistö S, Manunta P, Marette A, Matise TC, McKnight B, Meitinger T, Moll FL, Montgomery GW, Morris AD, Morris AP, Murray JC, Nelis M, Ohlsson C, Oldehinkel AJ, Ong KK, Ouwehand WH, Pasterkamp G, Peters A, Pramstaller PP, Price JF, Qi L, Raitakari OT, Rankinen T, Rao DC, Rice TK, Ritchie M, Rudan I, Salomaa V, Samani NJ, Saramies J, Sarzynski MA, Schwarz PEH, Sebert S, Sever P, Shuldiner AR, Sinisalo J, Steinthorsdottir V, Stolk RP, Tardif J-C, Tönjes A, Tremblay A, Tremoli E, Virtamo J, Vohl M-C, Electronic Medical Records and Genomics (eMEMERGEGE) Consortium, MIGen Consortium, PAGEGE Consortium, LifeLines Cohort Study, Amouyel P, Asselbergs FW, Assimes TL, Bochud M, Boehm BO, Boerwinkle E, Bottinger EP, Bouchard C, Cauchi S, Chambers JC, Chanock SJ, Cooper RS, de Bakker PIW, Dedoussis G, Ferrucci L, Franks PW, Froguel P, Groop LC, Haiman CA, Hamsten A, Hayes MG, Hui J, Hunter DJ, Hveem K, Jukema JW, Kaplan RC, Kivimaki M, Kuh D, Laakso M, Liu Y, Martin NG, März W, Melbye M, Moebus S, Munroe PB, Njølstad I, Oostra BA, Palmer CNA, Pedersen NL, Perola M, Pérusse L, Peters U, Powell JE, Power C, Quertermous T, Rauramaa R, Reinmaa E, Ridker PM, Rivadeneira F, Rotter JI, Saaristo TE, Saleheen D, Schlessinger D, Slagboom PE, Snieder H, Spector TD, Strauch K, Stumvoll M, Tuomilehto J, Uusitupa M, van der Harst P, Völzke H, Walker M, Wareham NJ, Watkins H, Wichmann H-E, Wilson JF, Zanen P, Deloukas P, Heid IM, Lindgren CM, Mohlke KL, Speliotes EK, Thorsteinsdottir U, Barroso I, Fox CS, North KE, Strachan DP, Beckmann JS, Berndt SI, Boehnke M, Borecki IB, McCarthy MI, Metspalu A, Stefansson K,

Uitterlinden AG, van Duijn CM, Franke L, Willer CJ, Price AL, Lettre G, Loos RJF, Weedon MN, Ingelsson E, O'Connell JR, Abecasis GR, Chasman DI, Goddard ME, Visscher PM, Hirschhorn JN, Frayling TM. 2014. Defining the role of common variation in the genomic and biological architecture of adult human height. Nat Genet. 46:1173–1186.
