# Supplementary material for: The Evolutionary History of Common Genetic Variants Influencing Human Cortical Surface Area
Source: Cereb Cortex. 2020 Dec 9;31(4):1873–87. doi: 10.1093/cercor/bhaa327 (PMC7945014; doi:10.1093/cercor/bhaa327)
Supplement: FigS11_bhaa327 [file figs11_bhaa327.pdf]

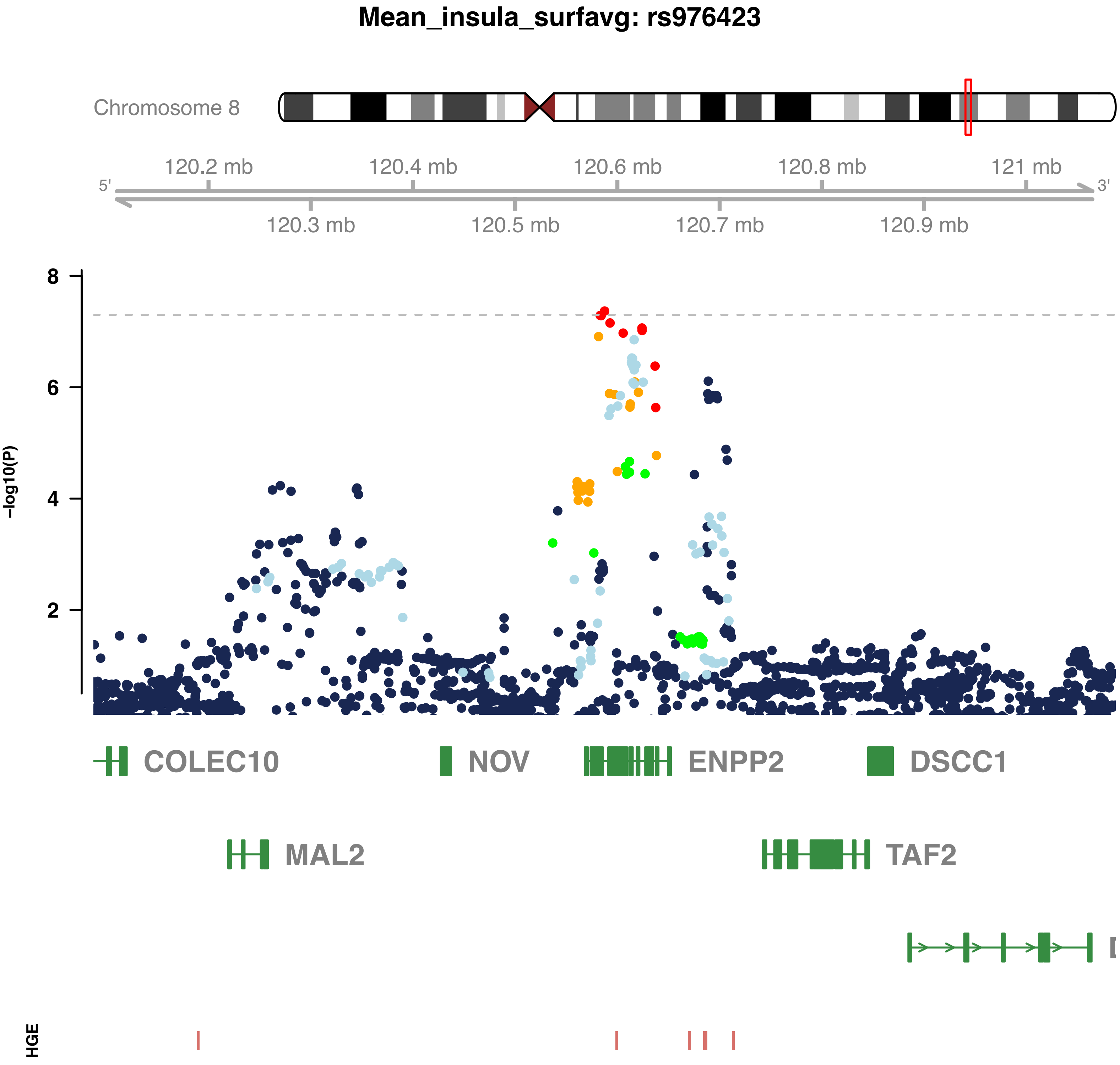

Mean_insula_surfavg: rs976423
Chromosome 8
120.2 mb
120.4 mb
120.6 mb
120.8 mb
121 mb
120.3 mb
120.5 mb
120.7 mb
120.9 mb
5'
3'
−log10(P)
8
6
4
2
COLEC10
NOV
ENPP2
DSCC1
MAL2
TAF2
HGE

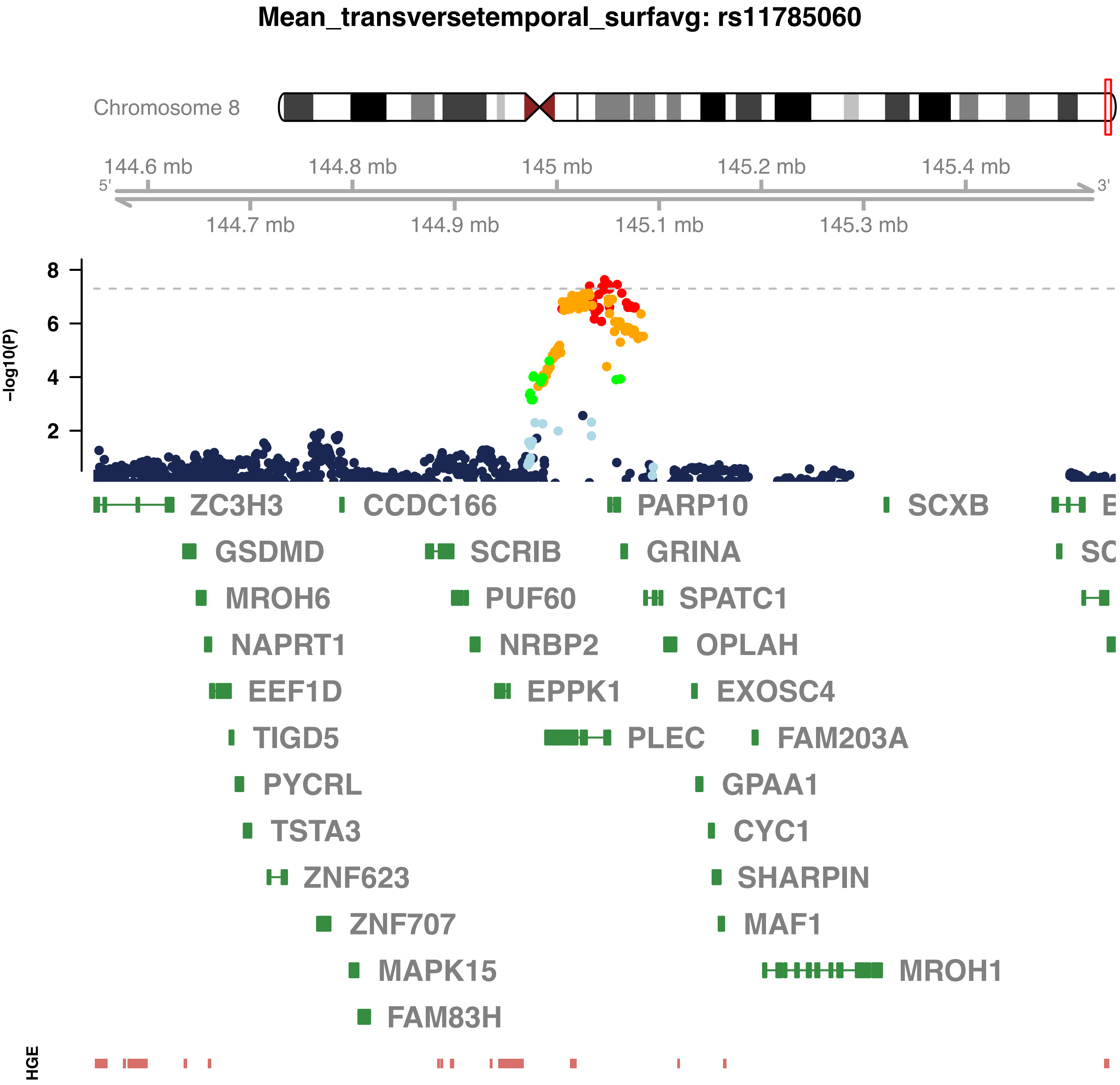

Mean_transversetemporal_surfavg: rs11785060
Chromosome 8
144.6 mb
144.8 mb
145 mb
145.2 mb
145.4 mb
5'
3'
144.7 mb
144.9 mb
145.1 mb
145.3 mb
-log10(P)
8
6
4
2
ZC3H3
CCDC166
PARP10
SCXB
GSDMD
SCRIB
GRINA
MROH6
PUF60
SPATC1
NAPRT1
NRBP2
OPLAH
EEF1D
EPPK1
EXOSC4
TIGD5
PLEC
FAM203A
PYCRL
GPAA1
TSTA3
CYC1
ZNF623
SHARPIN
ZNF707
MAF1
MAPK15
MROH1
FAM83H
HGE

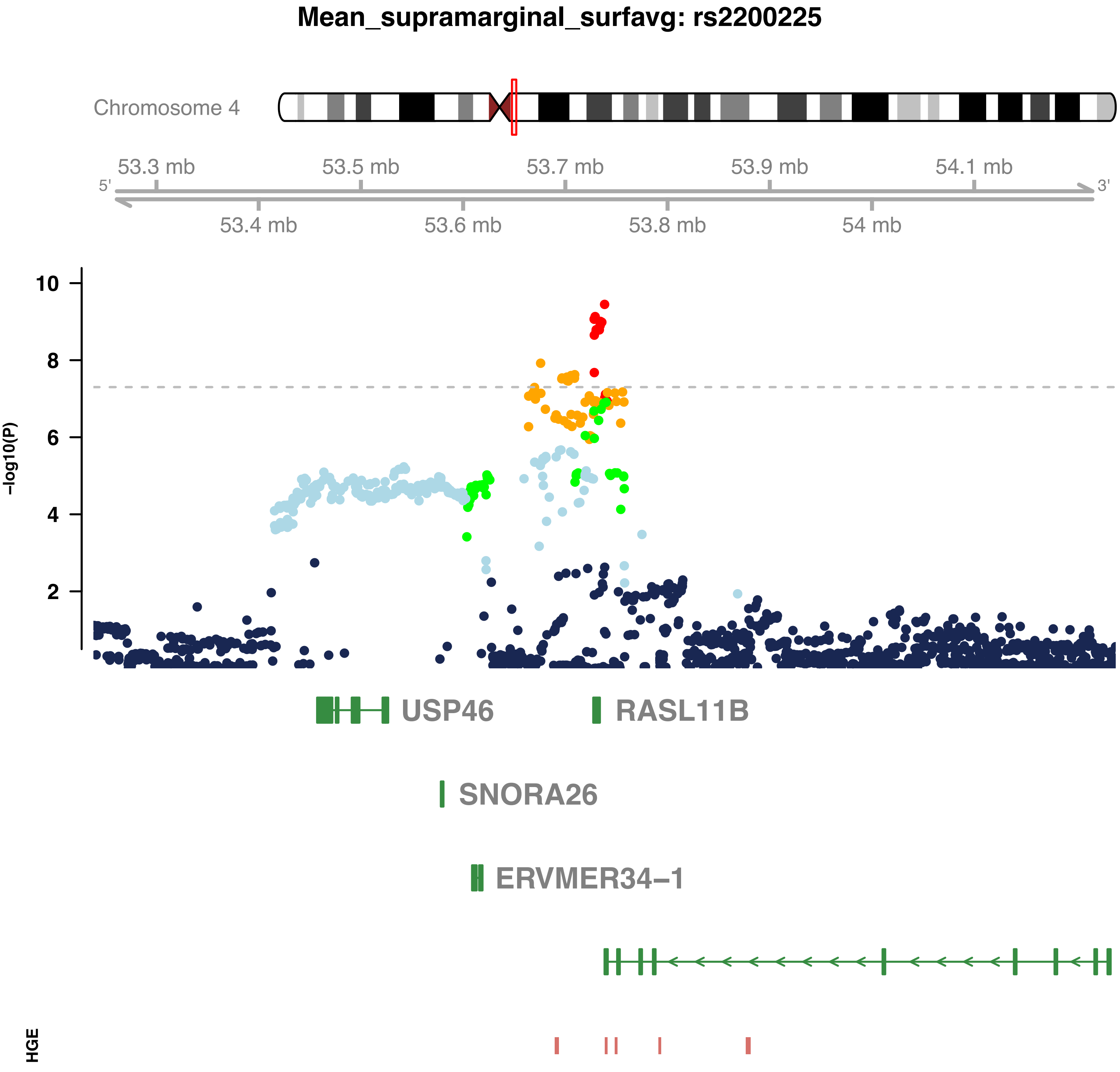
Mean_supramarginal_surfavg: rs2200225
Chromosome 4
53.3 mb
53.5 mb
53.7 mb
53.9 mb
54.1 mb
5'
3'
53.4 mb
53.6 mb
53.8 mb
54 mb
10
8
6
4
2
-log10(P)
USP46
RASL11B
SNORA26
ERVMER34-1
HGE

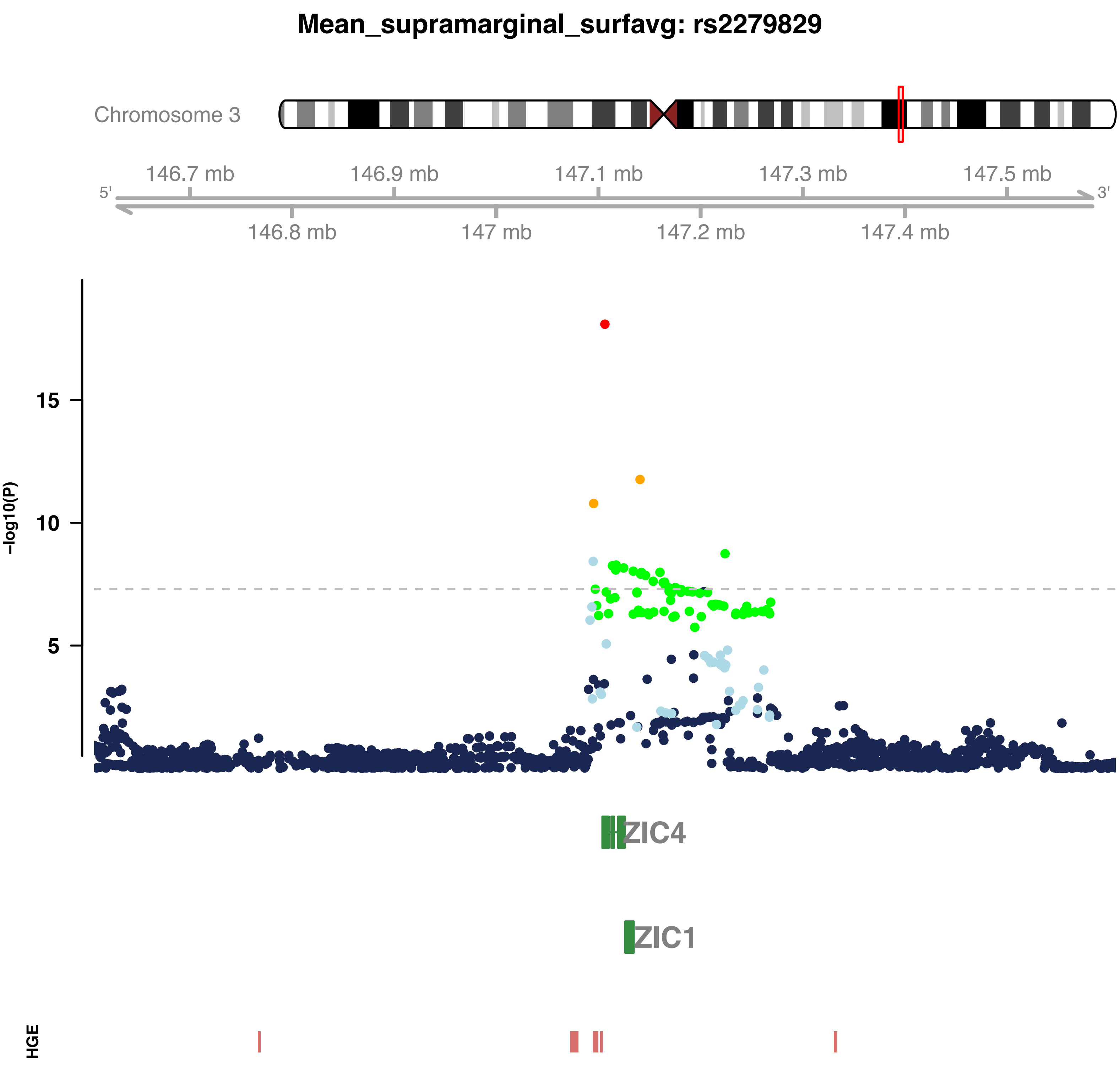
Mean_supramarginal_surfavg: rs2279829
Chromosome 3
146.7 mb
146.9 mb
147.1 mb
147.3 mb
147.5 mb
5'
3'
146.8 mb
147 mb
147.2 mb
147.4 mb
-log10(P)
15
10
5
ZIC4
ZIC1
HGE

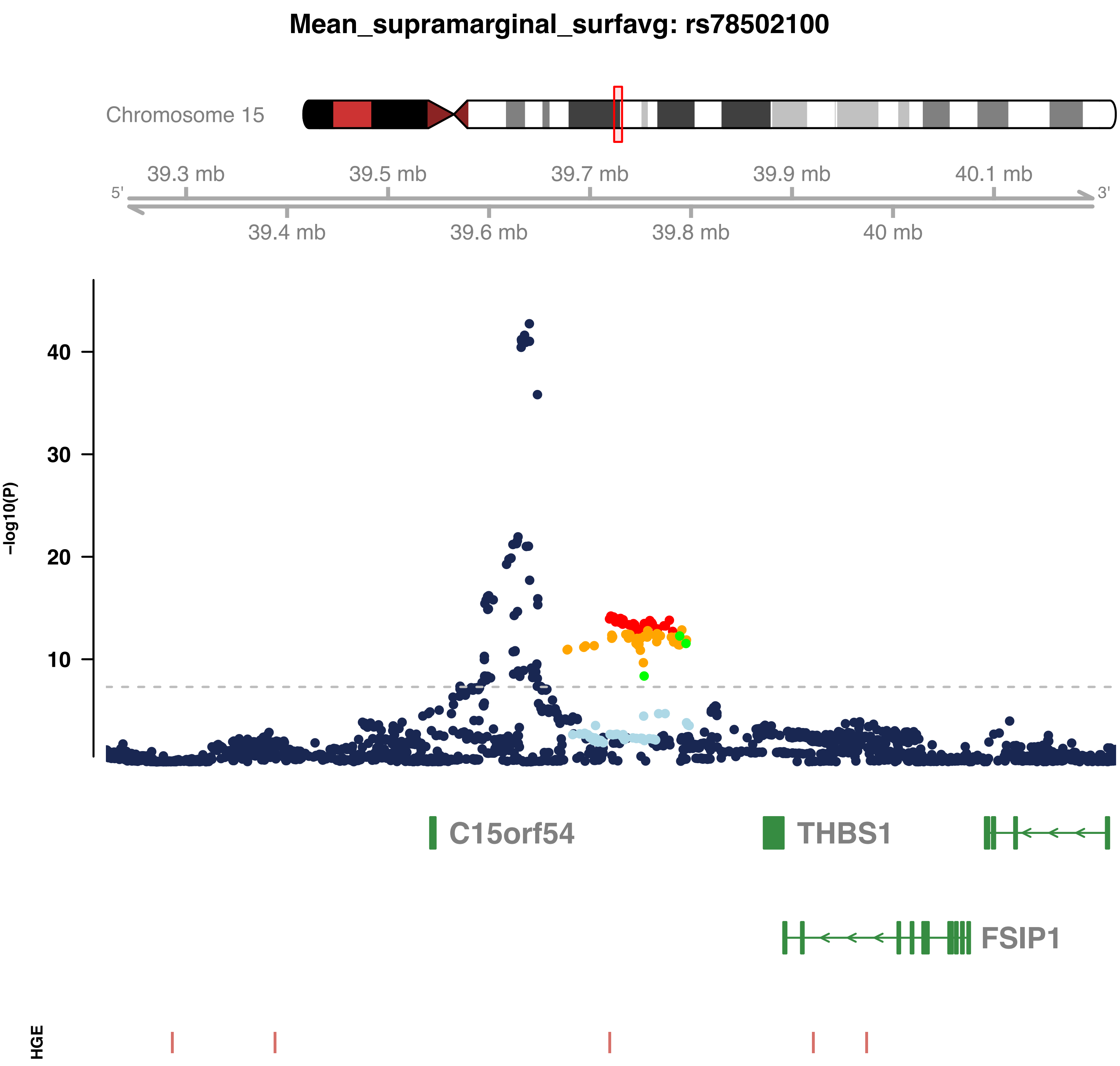

Mean_supramarginal_surfavg: rs78502100
Chromosome 15
39.3 mb
39.5 mb
39.7 mb
39.9 mb
40.1 mb
5'
3'
39.4 mb
39.6 mb
39.8 mb
40 mb
-log10(P)
40
30
20
10
C15orf54
THBS1
FSIP1
HGE

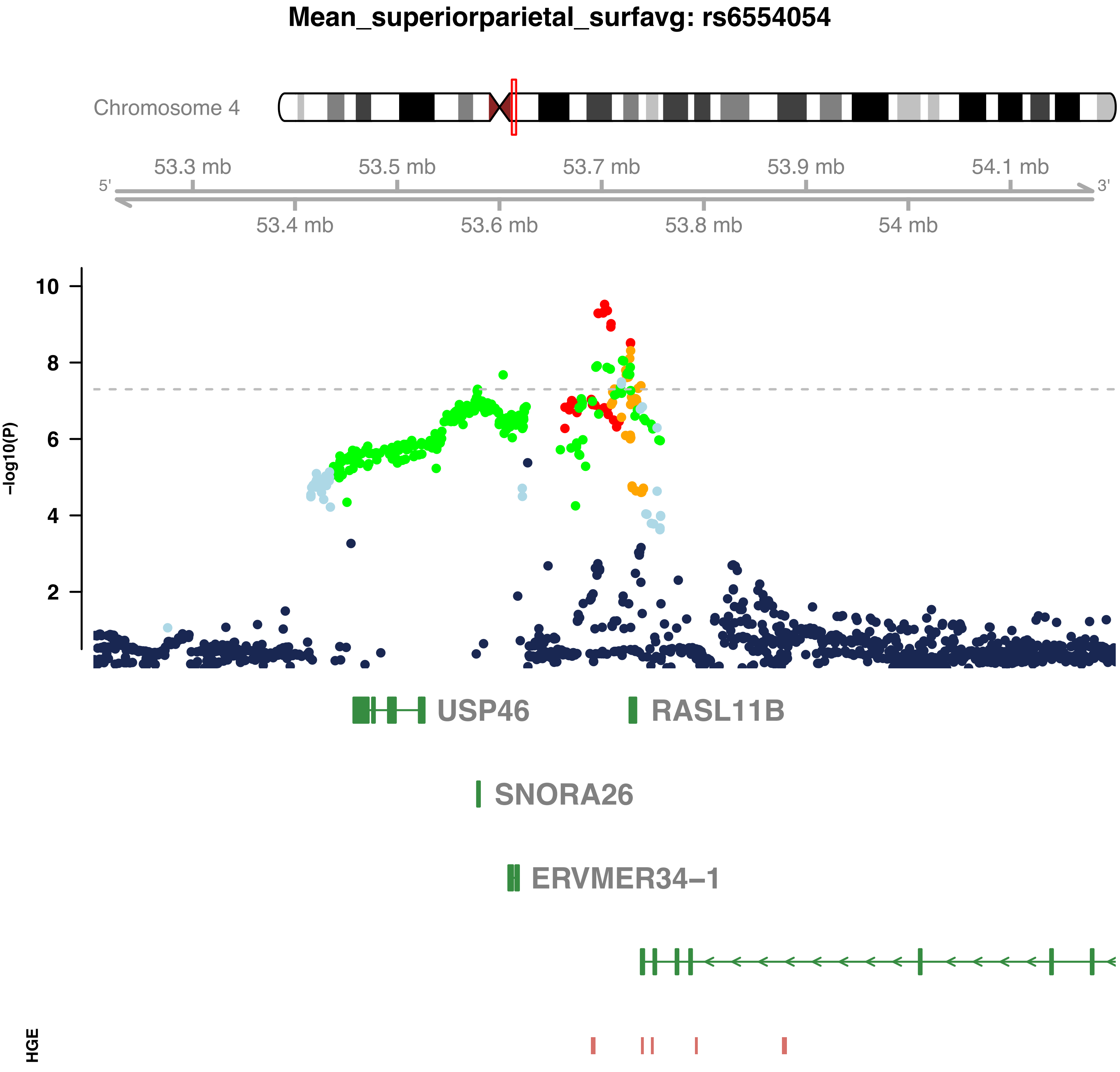

Mean_superiorparietal_surfavg: rs6554054
Chromosome 4
53.3 mb
53.5 mb
53.7 mb
53.9 mb
54.1 mb
5'
3'
53.4 mb
53.6 mb
53.8 mb
54 mb
10
8
6
4
2
-log10(P)
USP46
RASL11B
SNORA26
ERVMER34−1
HGE

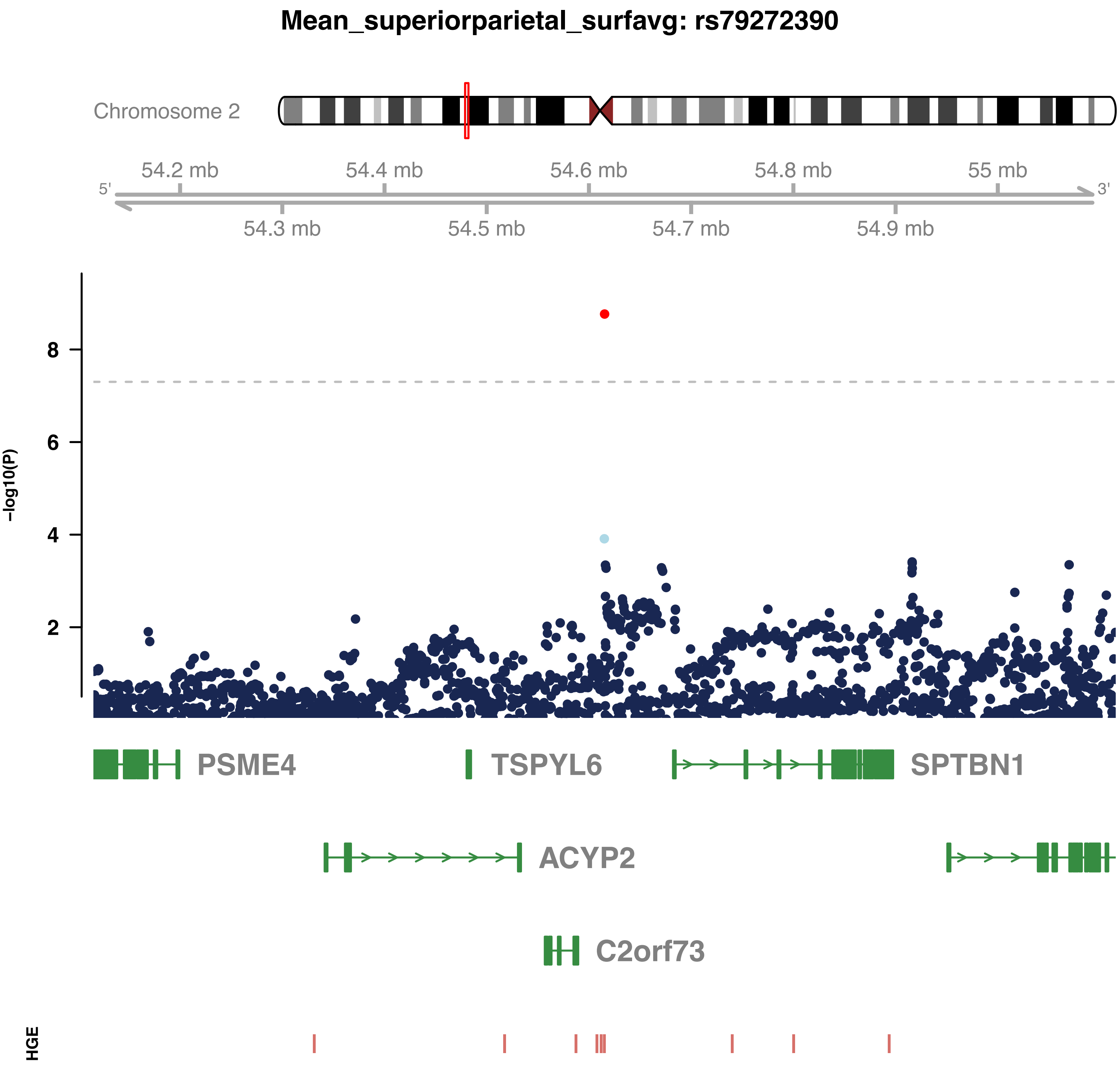

Mean_superiorparietal_surfavg: rs79272390
Chromosome 2
54.2 mb
54.4 mb
54.6 mb
54.8 mb
55 mb
5'
3'
54.3 mb
54.5 mb
54.7 mb
54.9 mb
-log10(P)
8
6
4
2
PSME4
TSPYL6
SPTBN1
ACYP2
C2orf73
HGE

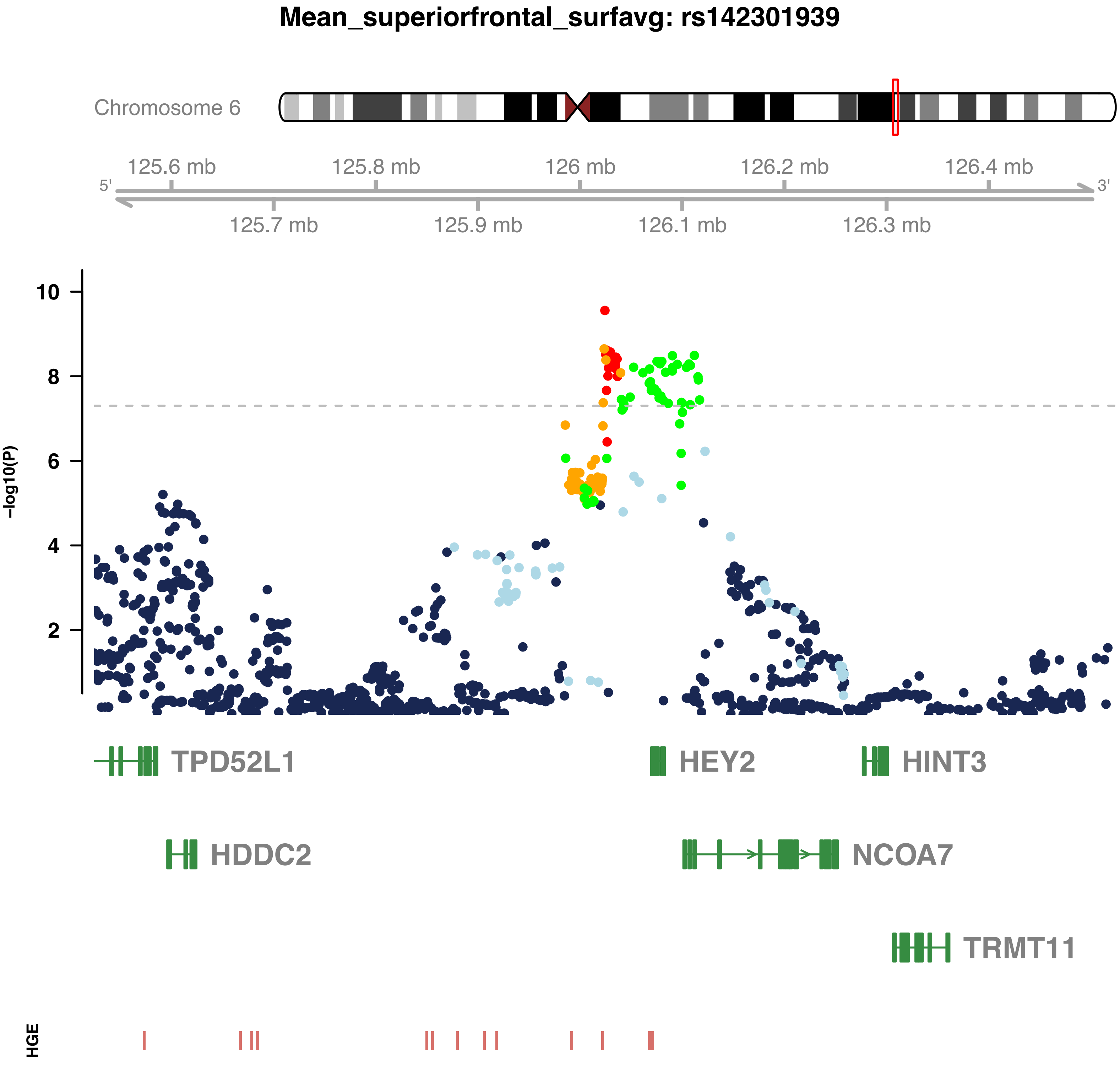

Mean_superiorfrontal_surfavg: rs142301939
Chromosome 6
125.6 mb
125.8 mb
126 mb
126.2 mb
126.4 mb
5'
3'
125.7 mb
125.9 mb
126.1 mb
126.3 mb
-log10(P)
10
8
6
4
2
TPD52L1
HEY2
HINT3
HDDC2
NCOA7
TRMT11
HGE

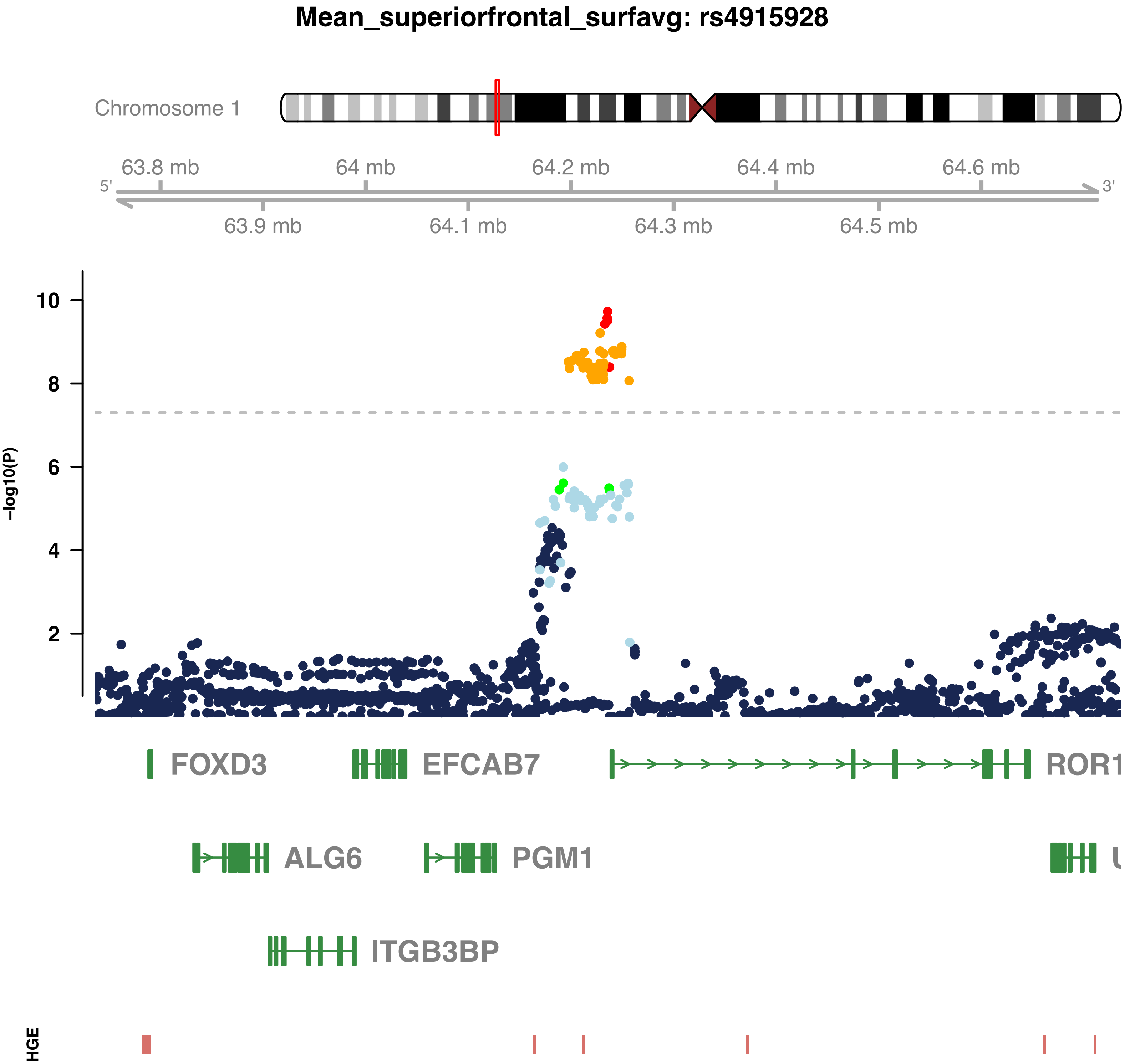
Mean_superiorfrontal_surfavg: rs4915928
Chromosome 1
63.8 mb
64 mb
64.2 mb
64.4 mb
64.6 mb
5'
3'
63.9 mb
64.1 mb
64.3 mb
64.5 mb
-log10(P)
10
8
6
4
2
FOXD3
EFCAB7
ROR1
ALG6
PGM1
ITGB3BP
HGE

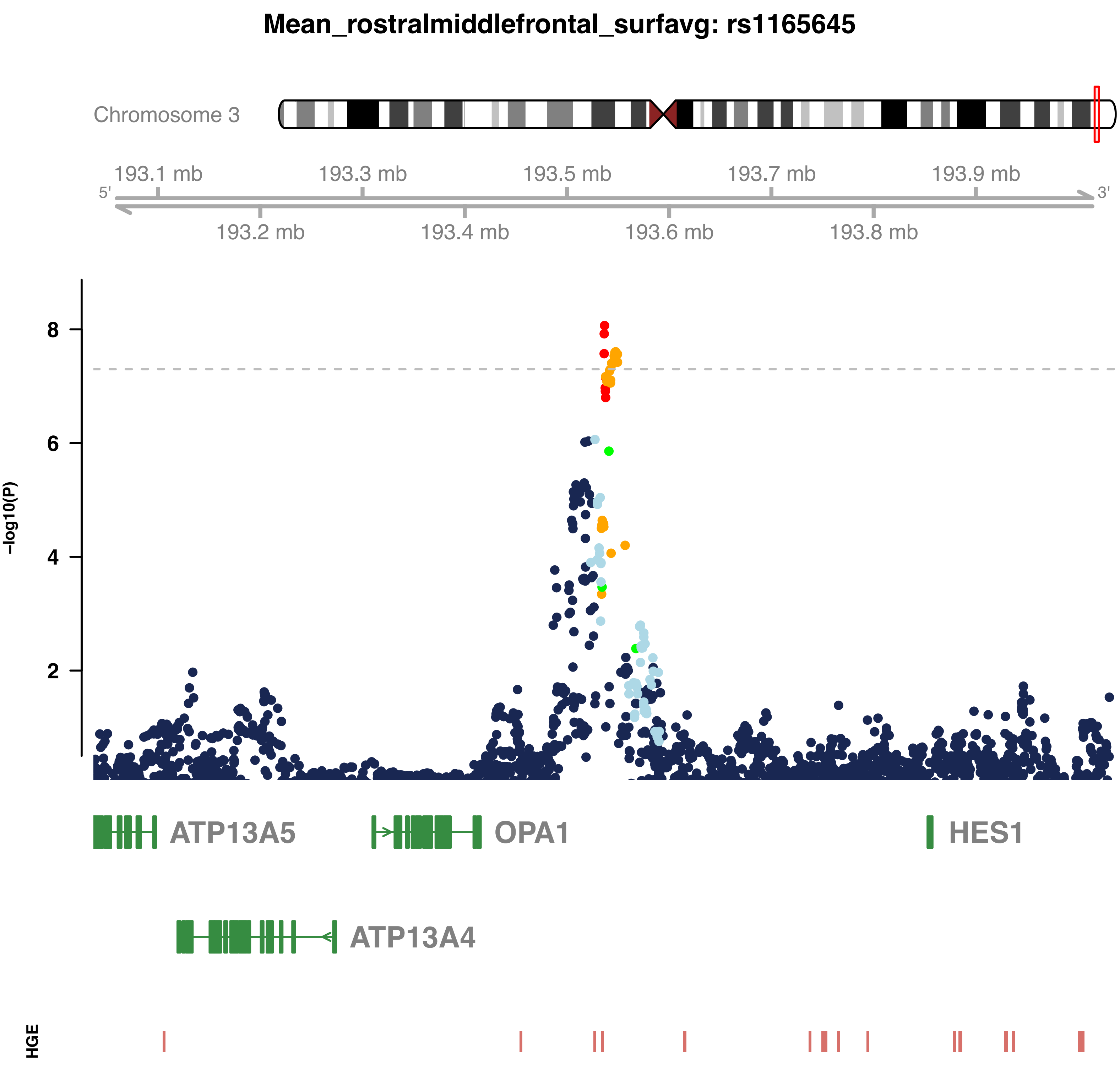
Mean_rostralmiddlefrontal_surfavg: rs1165645
Chromosome 3
193.1 mb
193.3 mb
193.5 mb
193.7 mb
193.9 mb
5'
3'
193.2 mb
193.4 mb
193.6 mb
193.8 mb
-log10(P)
8
6
4
2
ATP13A5
OPA1
HES1
ATP13A4
HGE

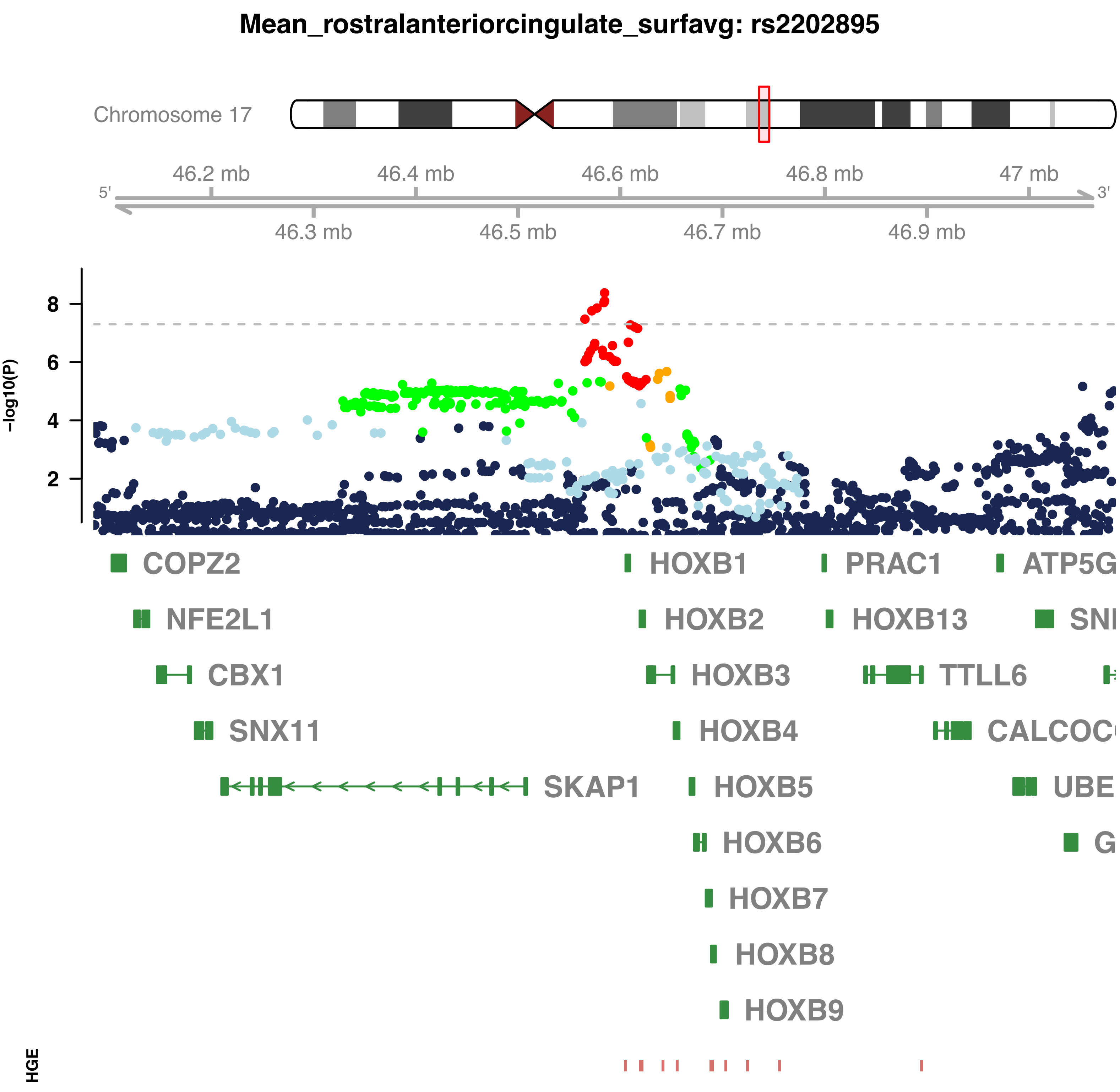

Mean_rostralanteriorcingulate_surfavg: rs2202895
Chromosome 17
46.2 mb
46.4 mb
46.6 mb
46.8 mb
47 mb
46.3 mb
46.5 mb
46.7 mb
46.9 mb
5'
3'
−log10(P)
8
6
4
2
COPZ2
NFE2L1
CBX1
SNX11
SKAP1
HOXB1
HOXB2
HOXB3
HOXB4
HOXB5
HOXB6
HOXB7
HOXB8
HOXB9
PRAC1
HOXB13
TTLL6
HGE

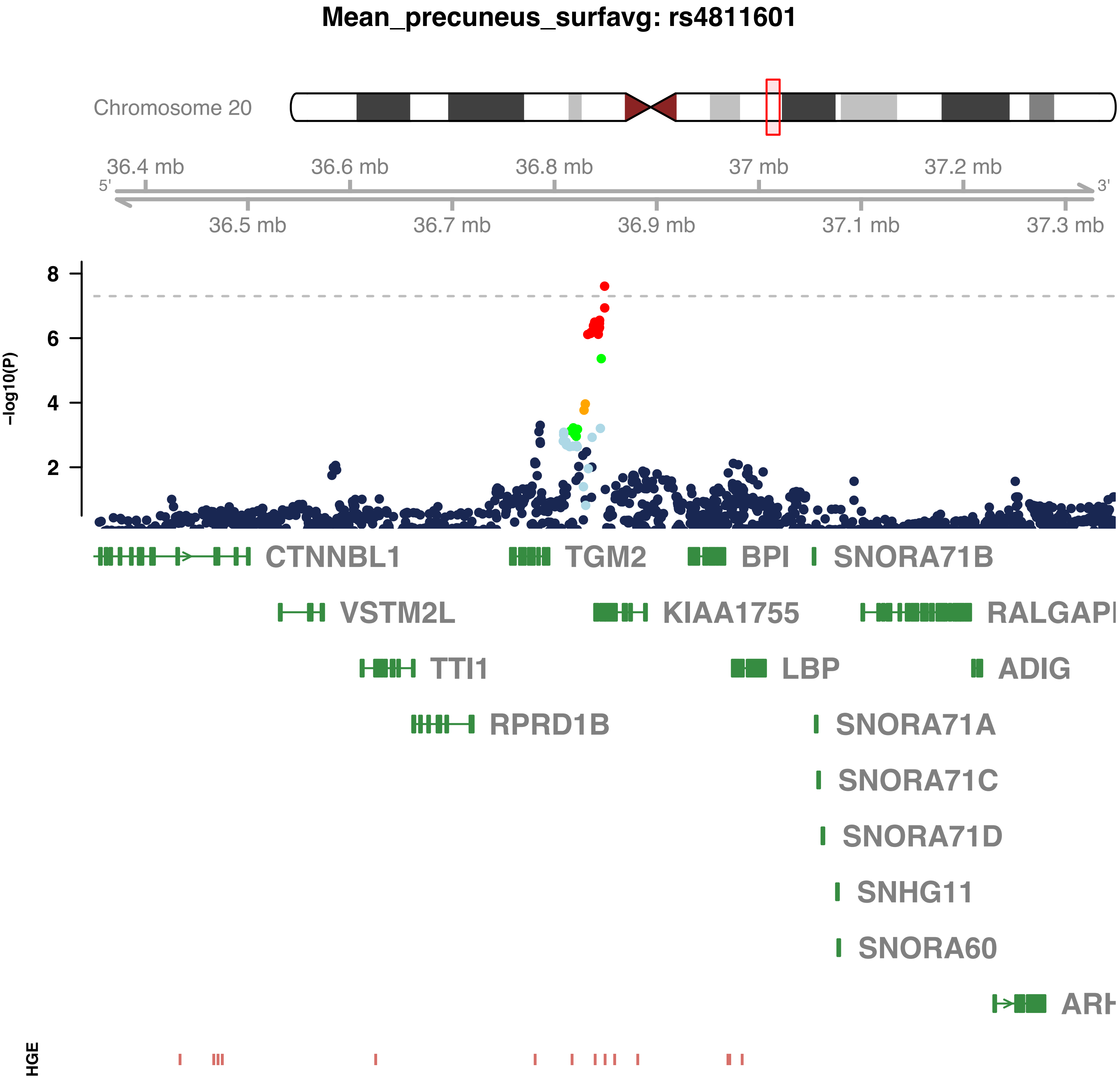
Mean_precuneus_surfavg: rs4811601
Chromosome 20
36.4 mb
36.6 mb
36.8 mb
37 mb
37.2 mb
5'
3'
36.5 mb
36.7 mb
36.9 mb
37.1 mb
37.3 mb
−log10(P)
8
6
4
2
CTNNBL1
TGM2
BPI
SNORA71B
VSTM2L
KIAA1755
TTI1
LBP
ADIG
RPRD1B
SNORA71A
SNORA71C
SNORA71D
SNHG11
SNORA60
HGE

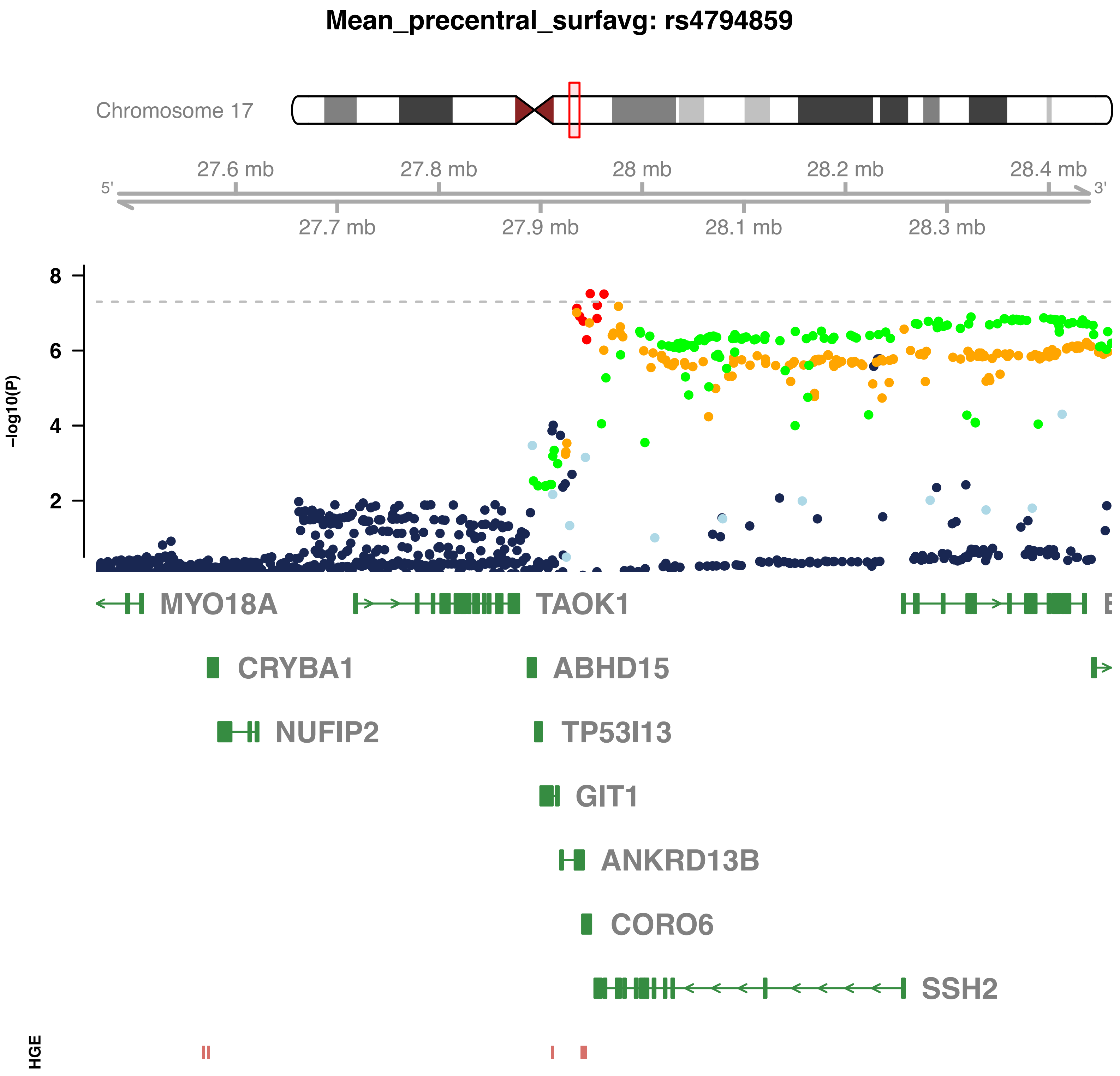
Mean_precentral_surfavg: rs4794859
Chromosome 17
27.6 mb
27.8 mb
28 mb
28.2 mb
28.4 mb
5'
3'
27.7 mb
27.9 mb
28.1 mb
28.3 mb
−log10(P)
8
6
4
2
MYO18A
TAOK1
CRYBA1
ABHD15
NUFIP2
TP53I13
GIT1
ANKRD13B
CORO6
SSH2
HGE

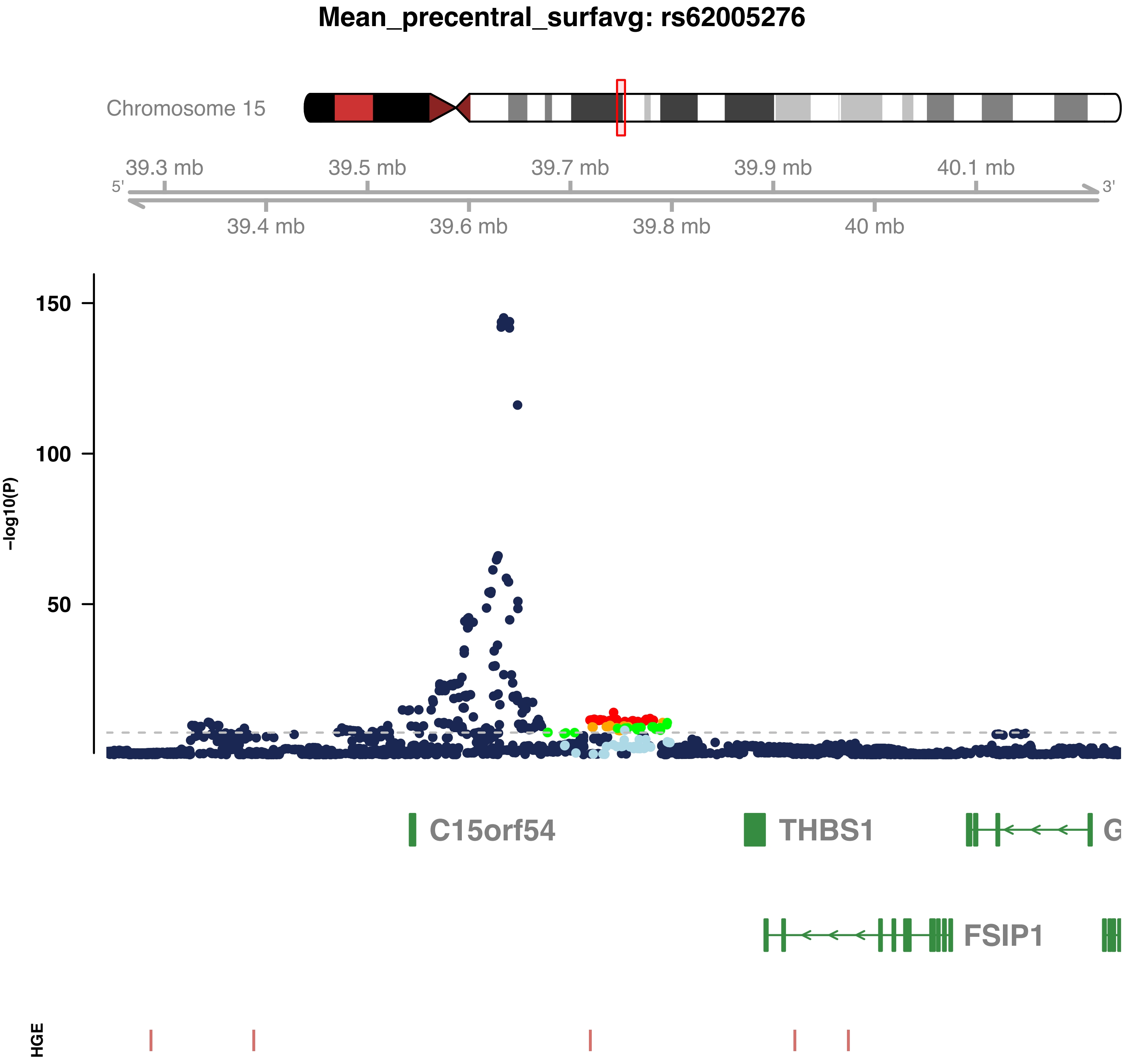

Mean_precentral_surfavg: rs62005276
Chromosome 15
39.3 mb
39.5 mb
39.7 mb
39.9 mb
40.1 mb
5'
3'
39.4 mb
39.6 mb
39.8 mb
40 mb
150
100
50
-log10(P)
C15orf54
THBS1
G
FSIP1
HGE

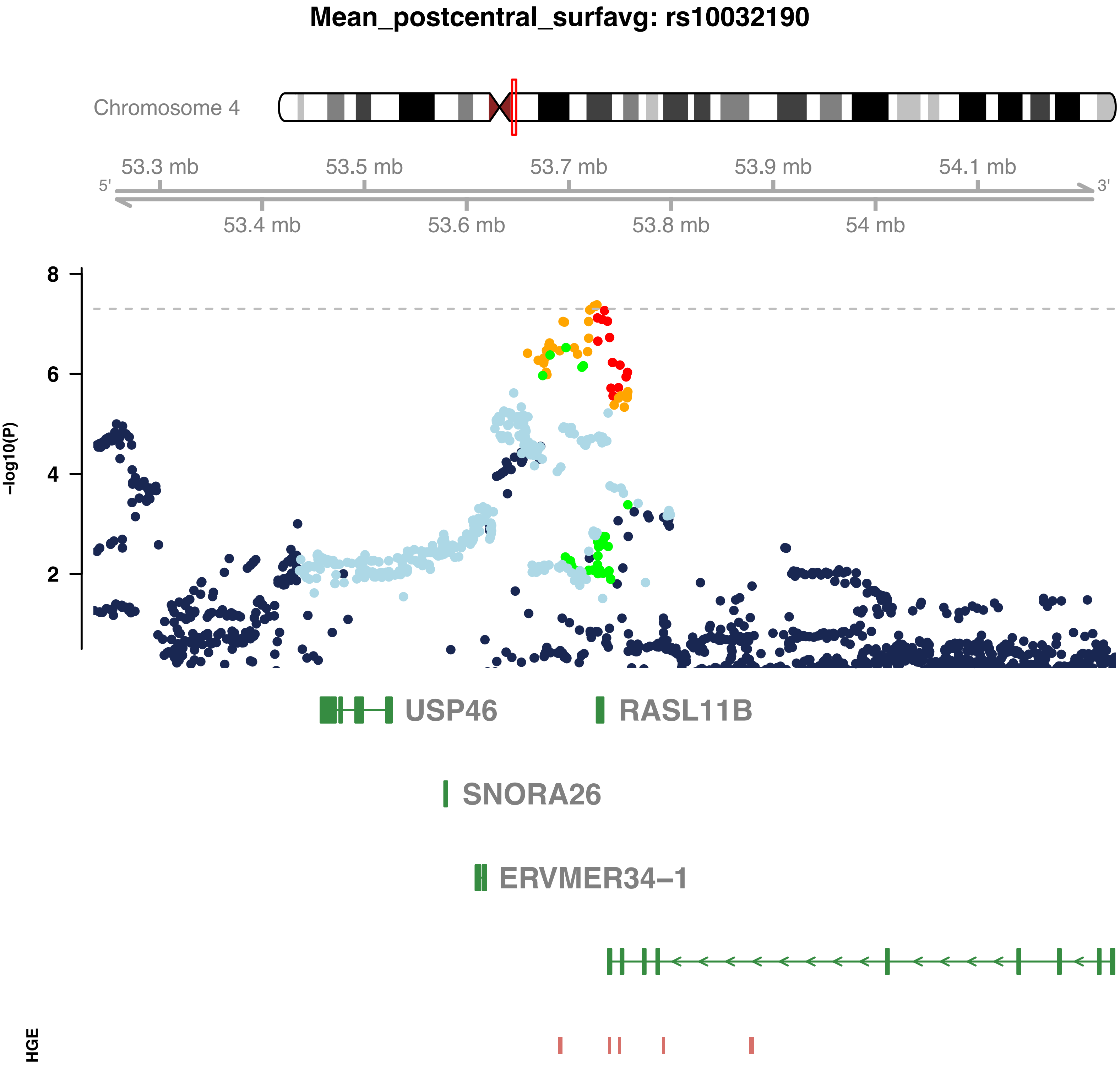
Mean_postcentral_surfavg: rs10032190
Chromosome 4
53.3 mb
53.5 mb
53.7 mb
53.9 mb
54.1 mb
5'
3'
53.4 mb
53.6 mb
53.8 mb
54 mb
−log10(P)
8
6
4
2
USP46
RASL11B
SNORA26
ERVMER34−1
HGE

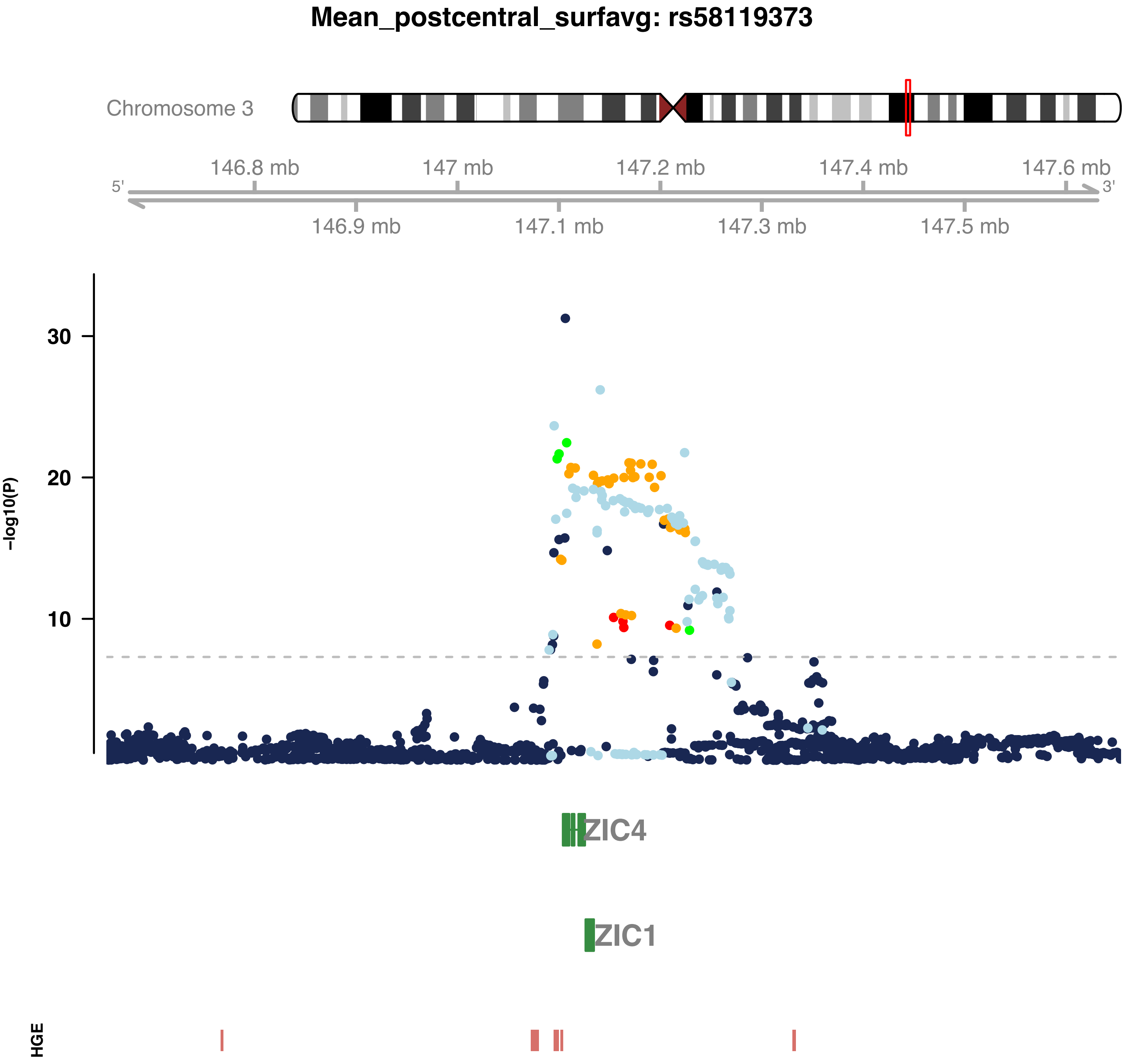

Mean_postcentral_surfavg: rs58119373
Chromosome 3
146.8 mb
147 mb
147.2 mb
147.4 mb
147.6 mb
5'
3'
146.9 mb
147.1 mb
147.3 mb
147.5 mb
30
20
10
-log10(P)
ZIC4
ZIC1
HGE

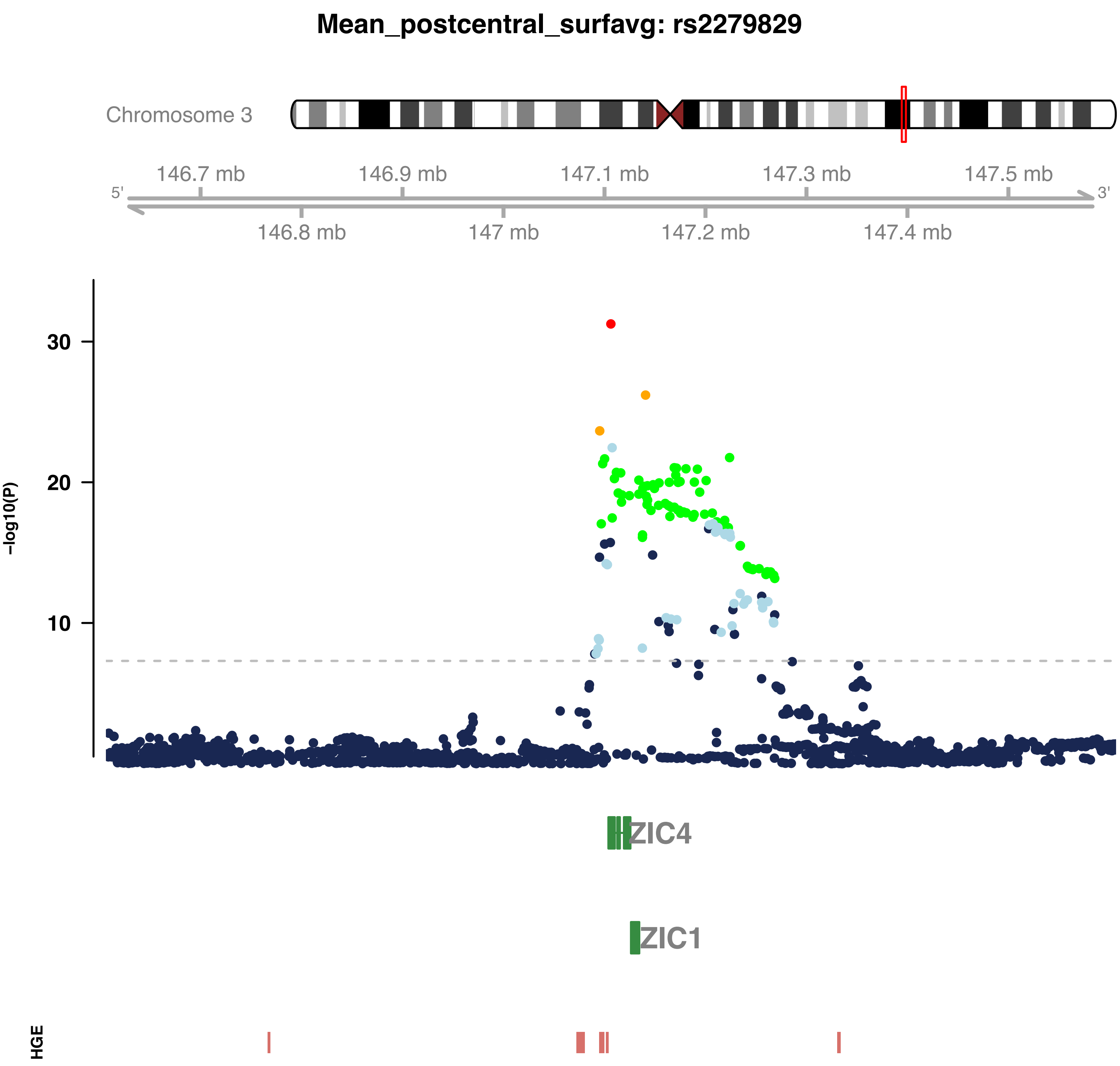
Mean_postcentral_surfavg: rs2279829
Chromosome 3
146.7 mb
146.9 mb
147.1 mb
147.3 mb
147.5 mb
5'
3'
146.8 mb
147 mb
147.2 mb
147.4 mb
30
20
10
-log10(P)
ZIC4
ZIC1
HGE

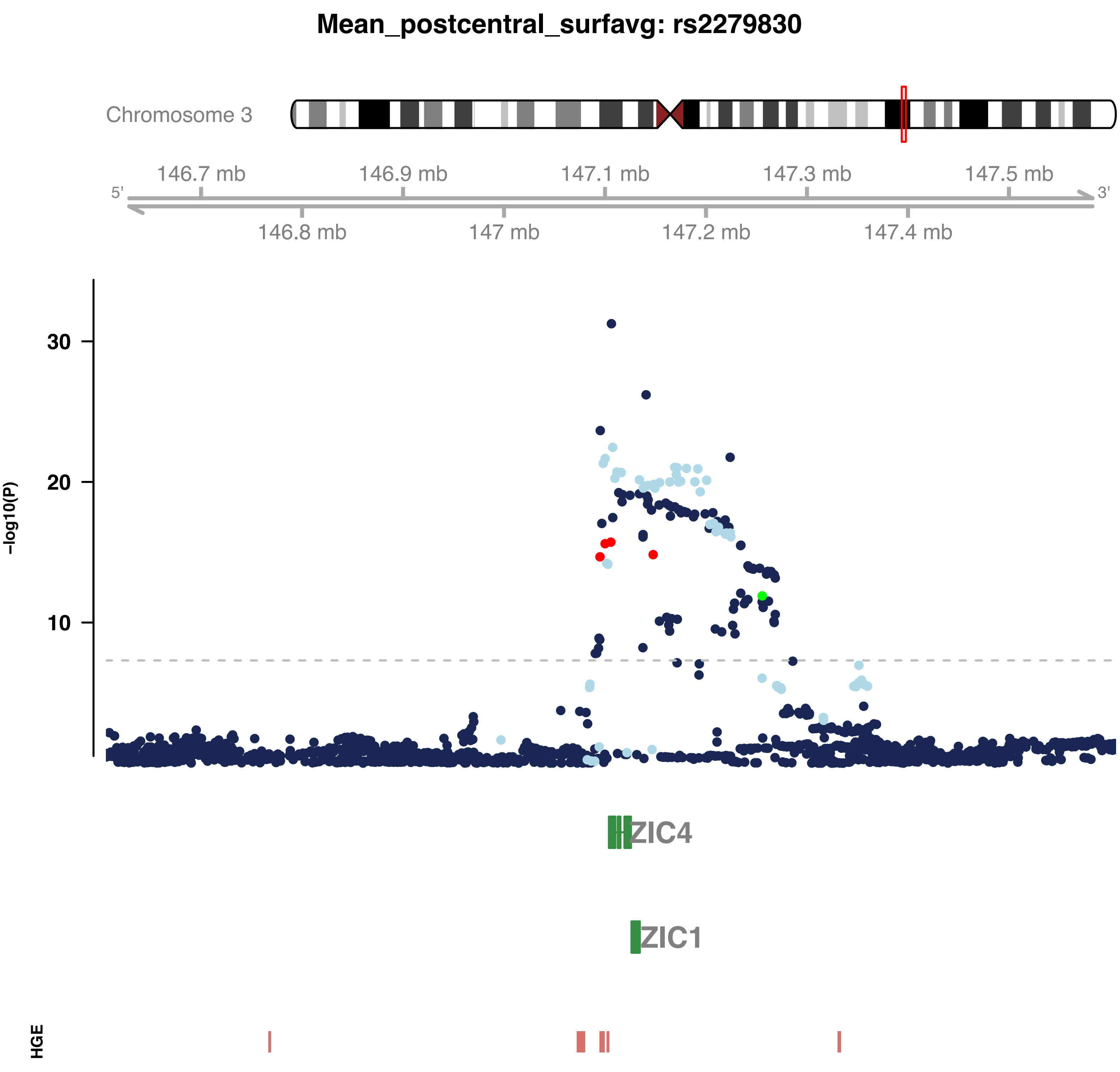
Mean_postcentral_surfavg: rs2279830
Chromosome 3
146.7 mb
146.9 mb
147.1 mb
147.3 mb
147.5 mb
146.8 mb
147 mb
147.2 mb
147.4 mb
5'
3'
-log10(P)
30
20
10
ZIC4
ZIC1
HGE

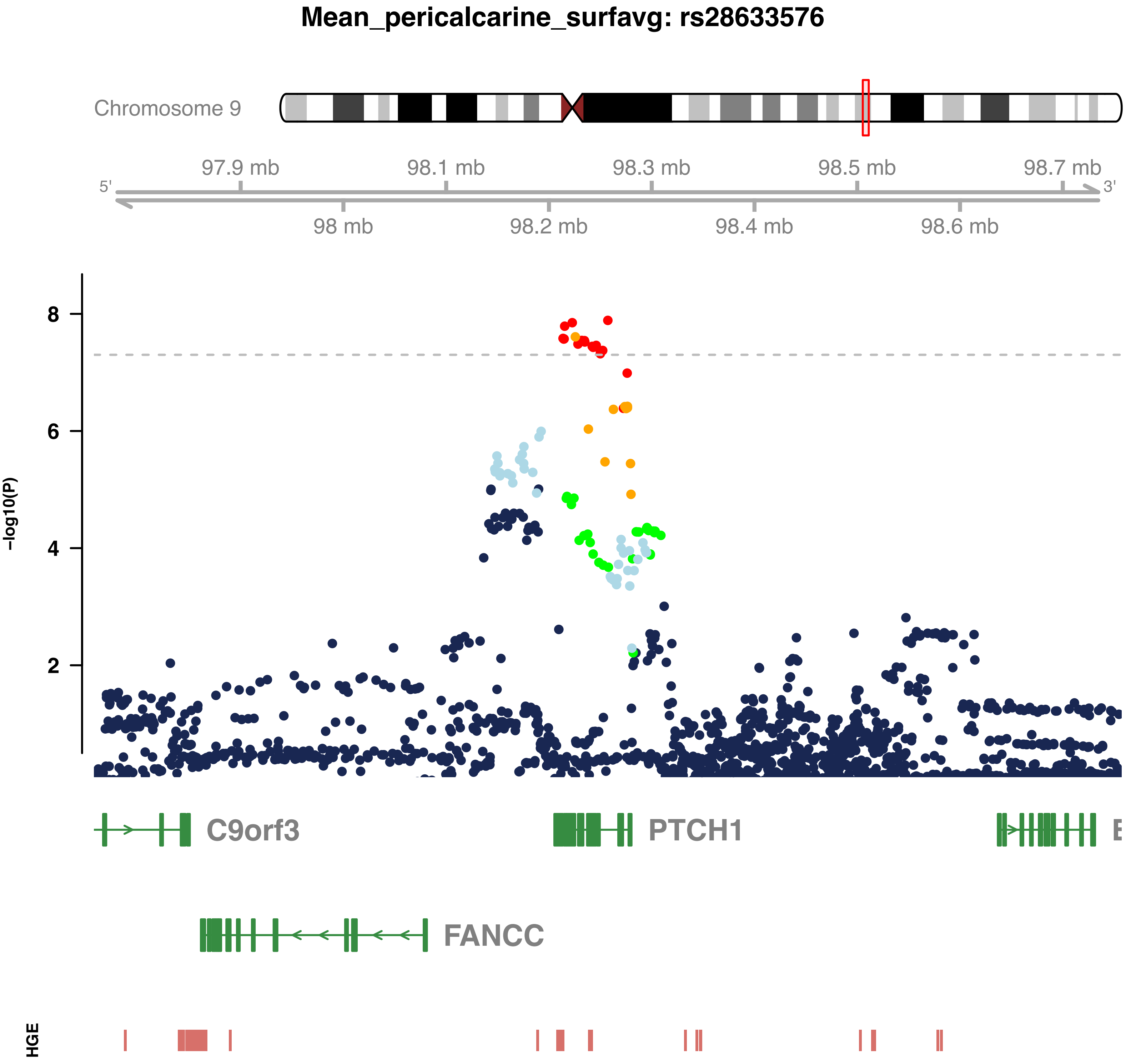

Mean_pericalcarine_surfavg: rs28633576
Chromosome 9
97.9 mb
98.1 mb
98.3 mb
98.5 mb
98.7 mb
5'
3'
98 mb
98.2 mb
98.4 mb
98.6 mb
−log10(P)
8
6
4
2
C9orf3
PTCH1
FANCC
HGE

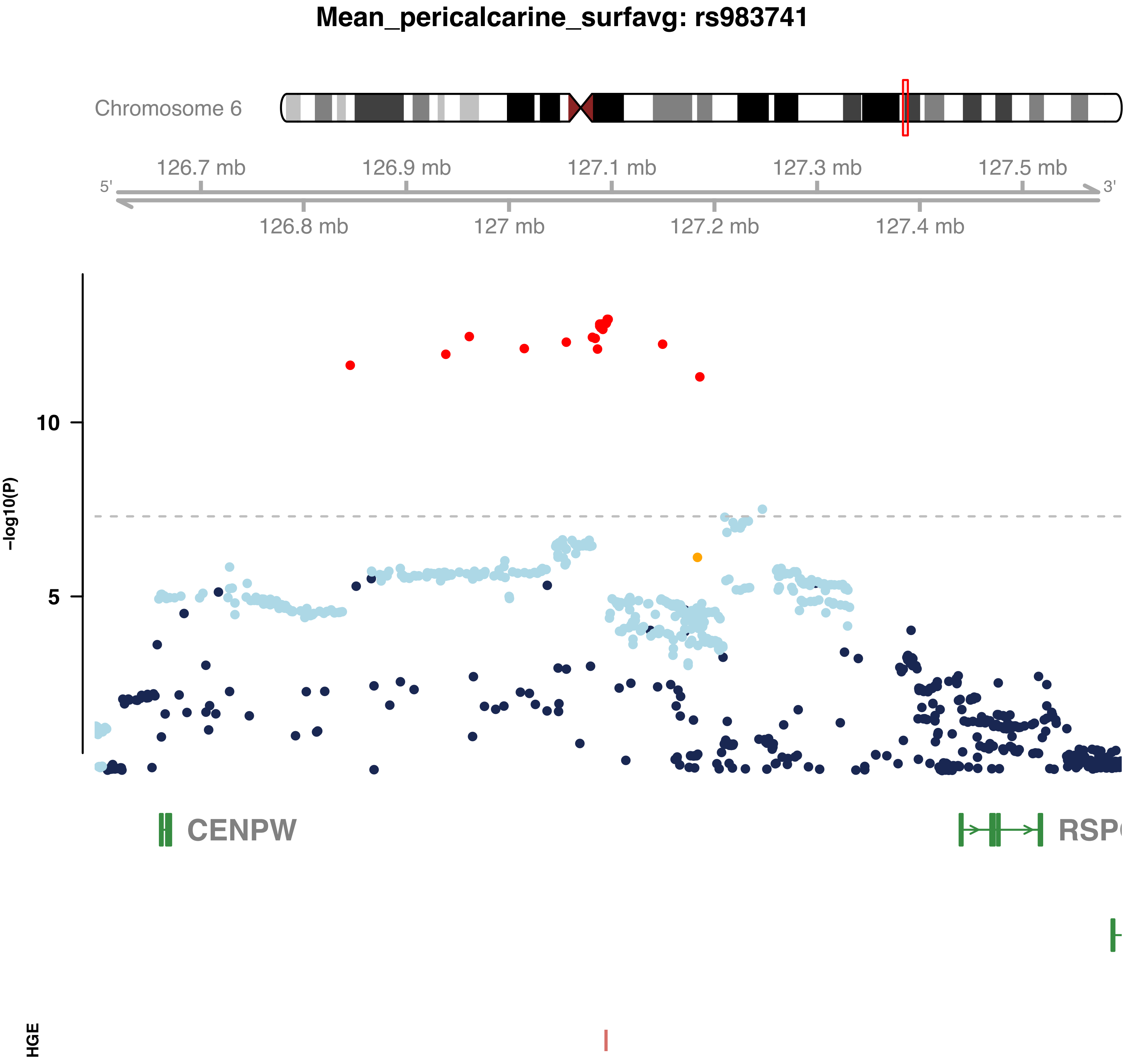

Mean_pericalcarine_surfavg: rs983741
Chromosome 6
126.7 mb
126.9 mb
127.1 mb
127.3 mb
127.5 mb
5'
3'
126.8 mb
127 mb
127.2 mb
127.4 mb
10
5
-log10(P)
CENPW
HGE

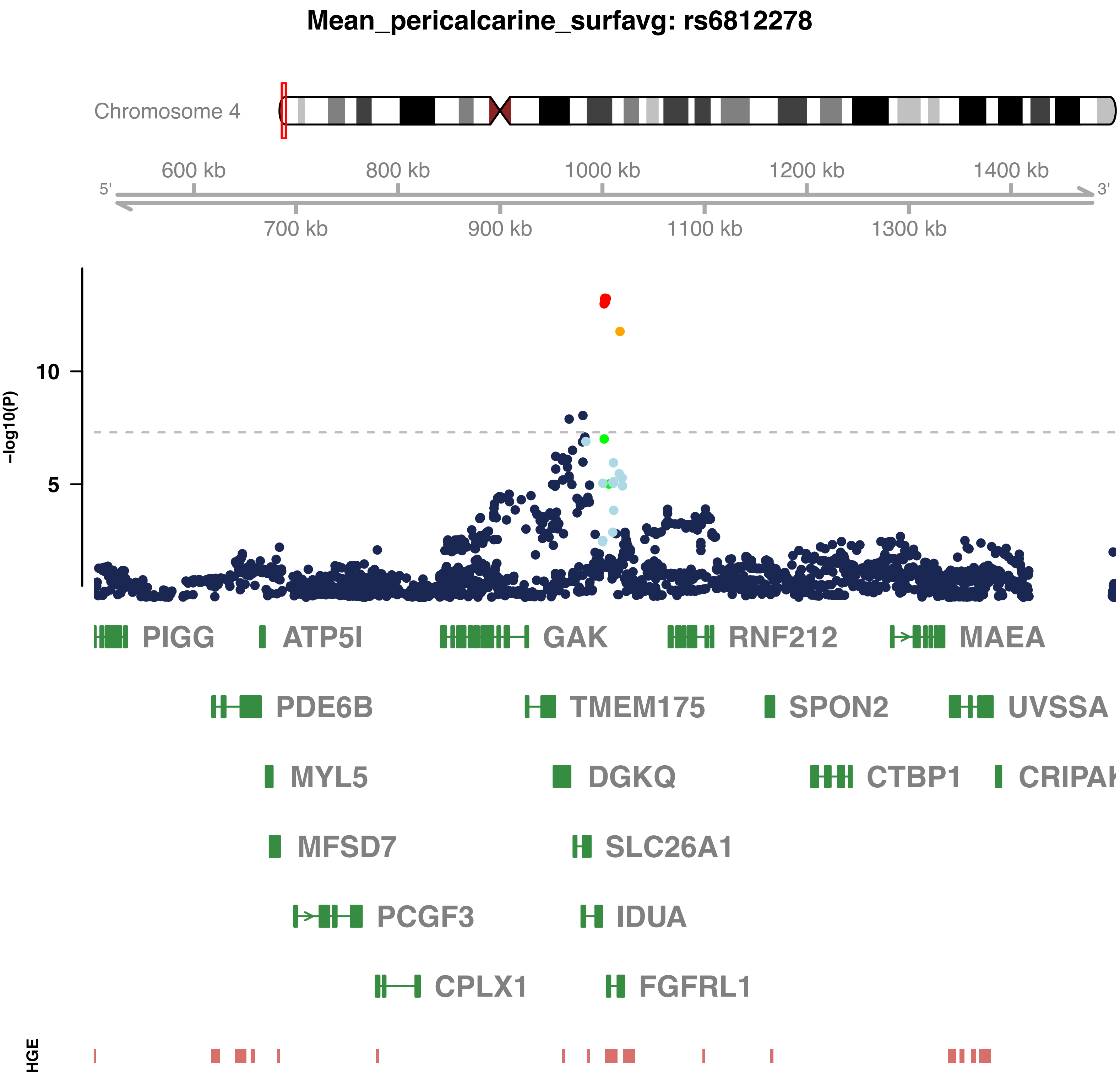

Mean_pericalcarine_surfavg: rs6812278
Chromosome 4
600 kb
800 kb
1000 kb
1200 kb
1400 kb
5'
3'
700 kb
900 kb
1100 kb
1300 kb
-log10(P)
10
5
PIGG
ATP5I
GAK
RNF212
MAEA
PDE6B
TMEM175
SPON2
UVSSA
MYL5
DGKQ
CTBP1
MFSD7
SLC26A1
PCGF3
IDUA
CPLX1
FGFRL1
HGE

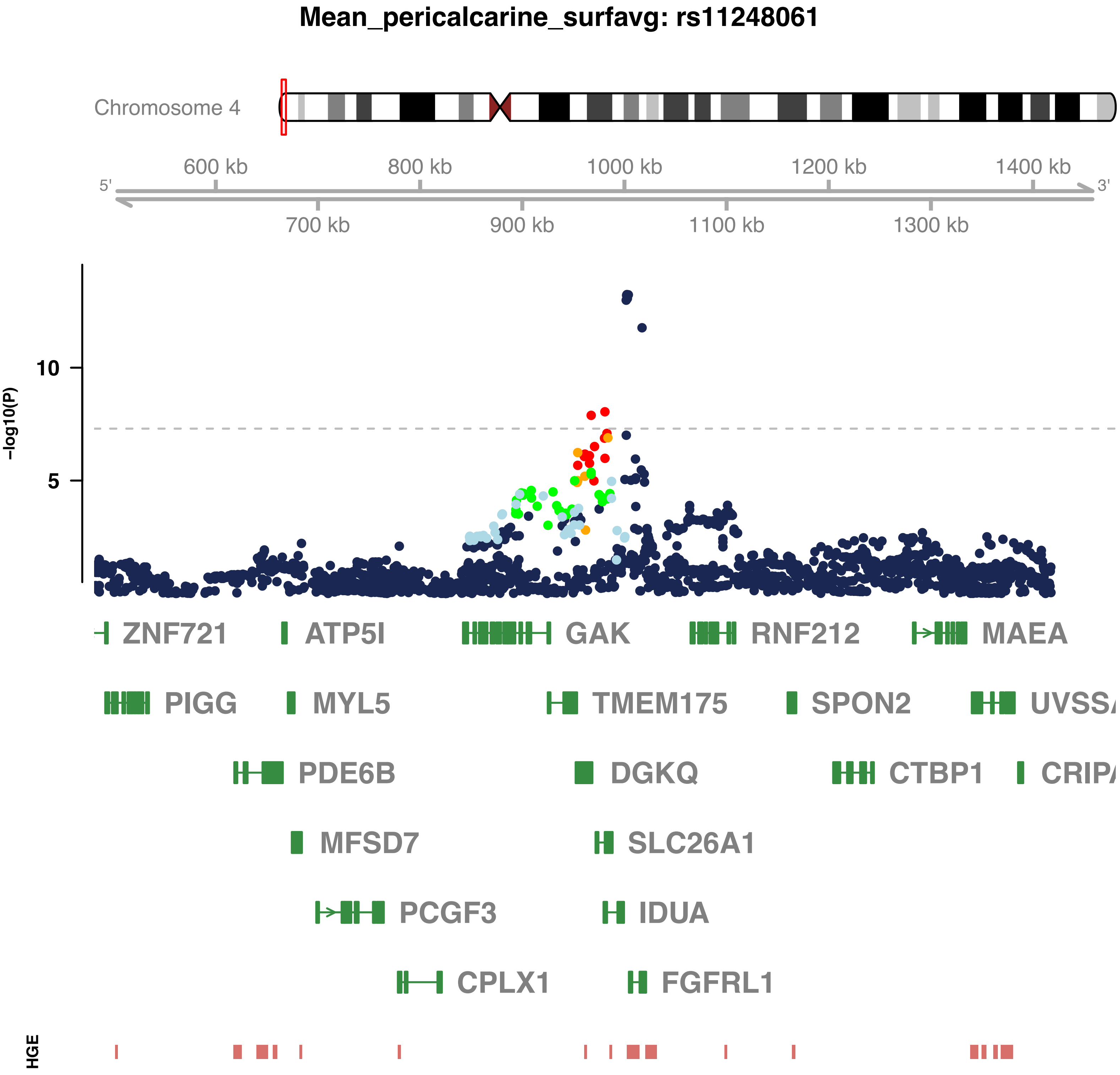

Mean_pericalcarine_surfavg: rs11248061
Chromosome 4
600 kb
800 kb
1000 kb
1200 kb
1400 kb
5'
3'
700 kb
900 kb
1100 kb
1300 kb
-log10(P)
10
5
ZNF721
ATP5I
GAK
RNF212
MAEA
PIGG
MYL5
TMEM175
SPON2
PDE6B
DGKQ
CTBP1
MFSD7
SLC26A1
PCGF3
IDUA
CPLX1
FGFRL1
HGE

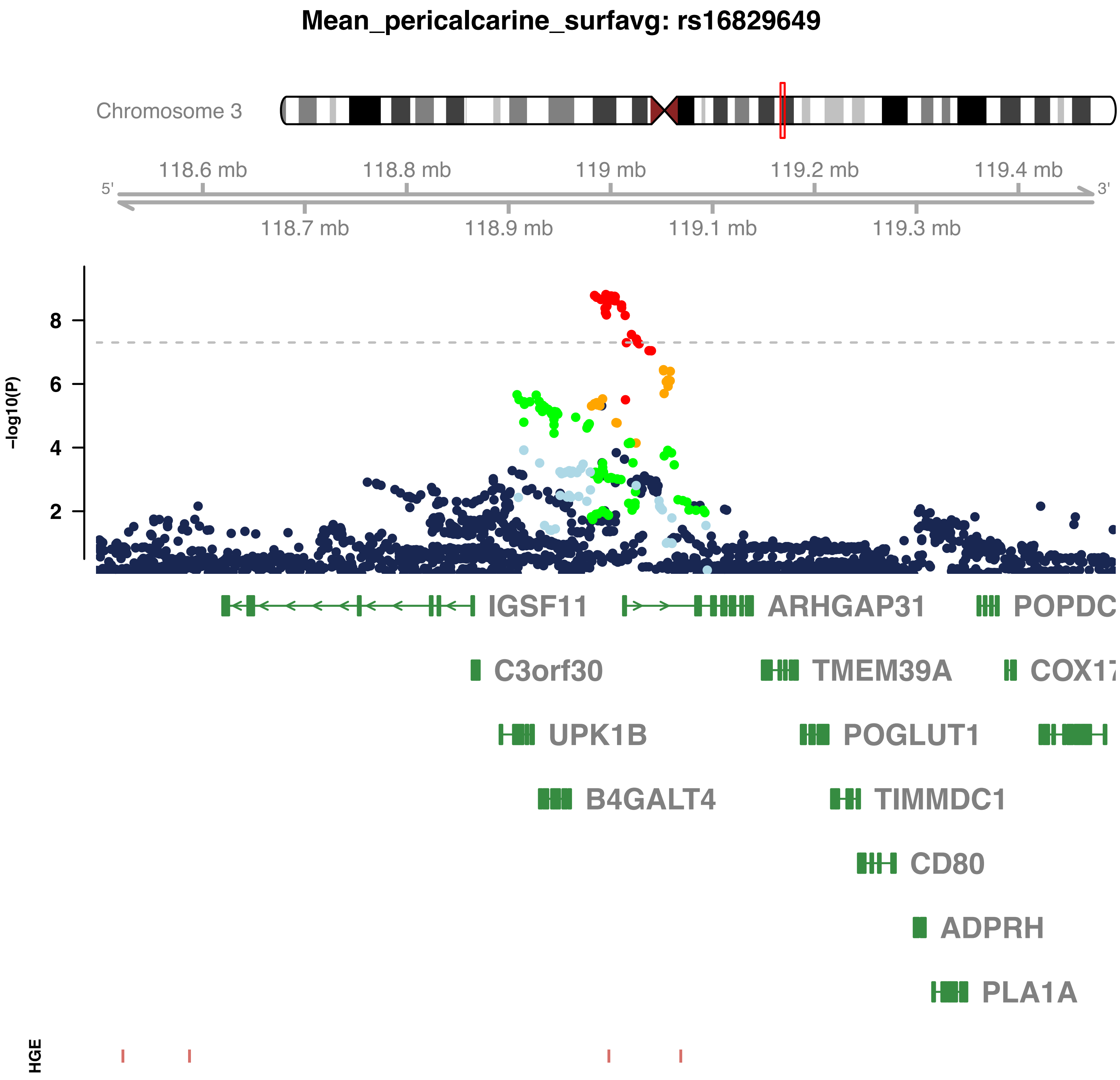

Mean_pericalcarine_surfavg: rs16829649
Chromosome 3
118.6 mb
118.8 mb
119 mb
119.2 mb
119.4 mb
118.7 mb
118.9 mb
119.1 mb
119.3 mb
5'
3'
-log10(P)
2
4
6
8
IGSF11
ARHGAP31
POPDC
C3orf30
TMEM39A
COX17
UPK1B
POGLUT1
B4GALT4
TIMMDC1
CD80
ADPRH
PLA1A
HGE

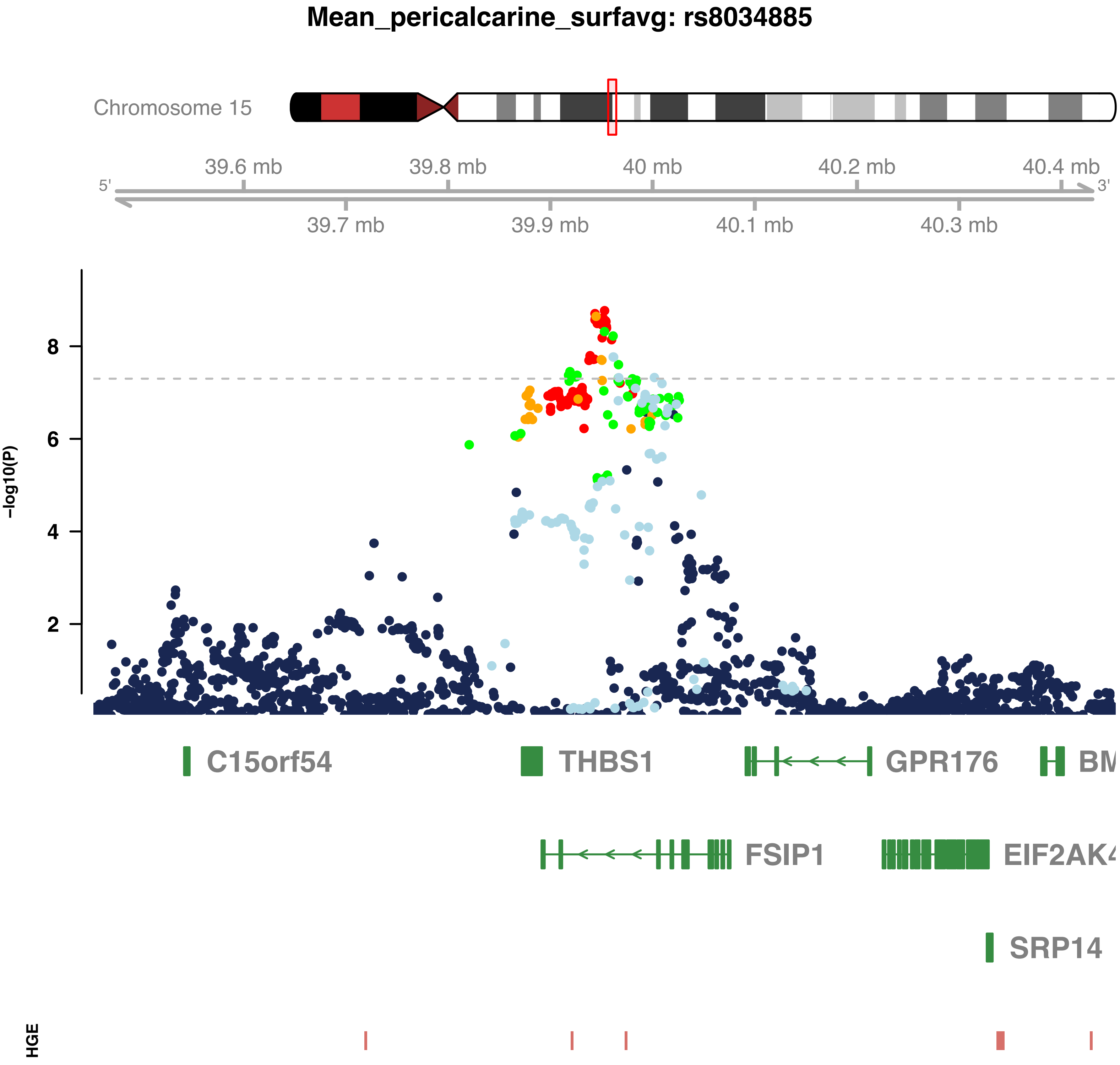
Mean_pericalcarine_surfavg: rs8034885
Chromosome 15
39.6 mb
39.8 mb
40 mb
40.2 mb
40.4 mb
5'
3'
39.7 mb
39.9 mb
40.1 mb
40.3 mb
-log10(P)
8
6
4
2
C15orf54
THBS1
GPR176
BM
FSIP1
EIF2AK4
SRP14
HGE

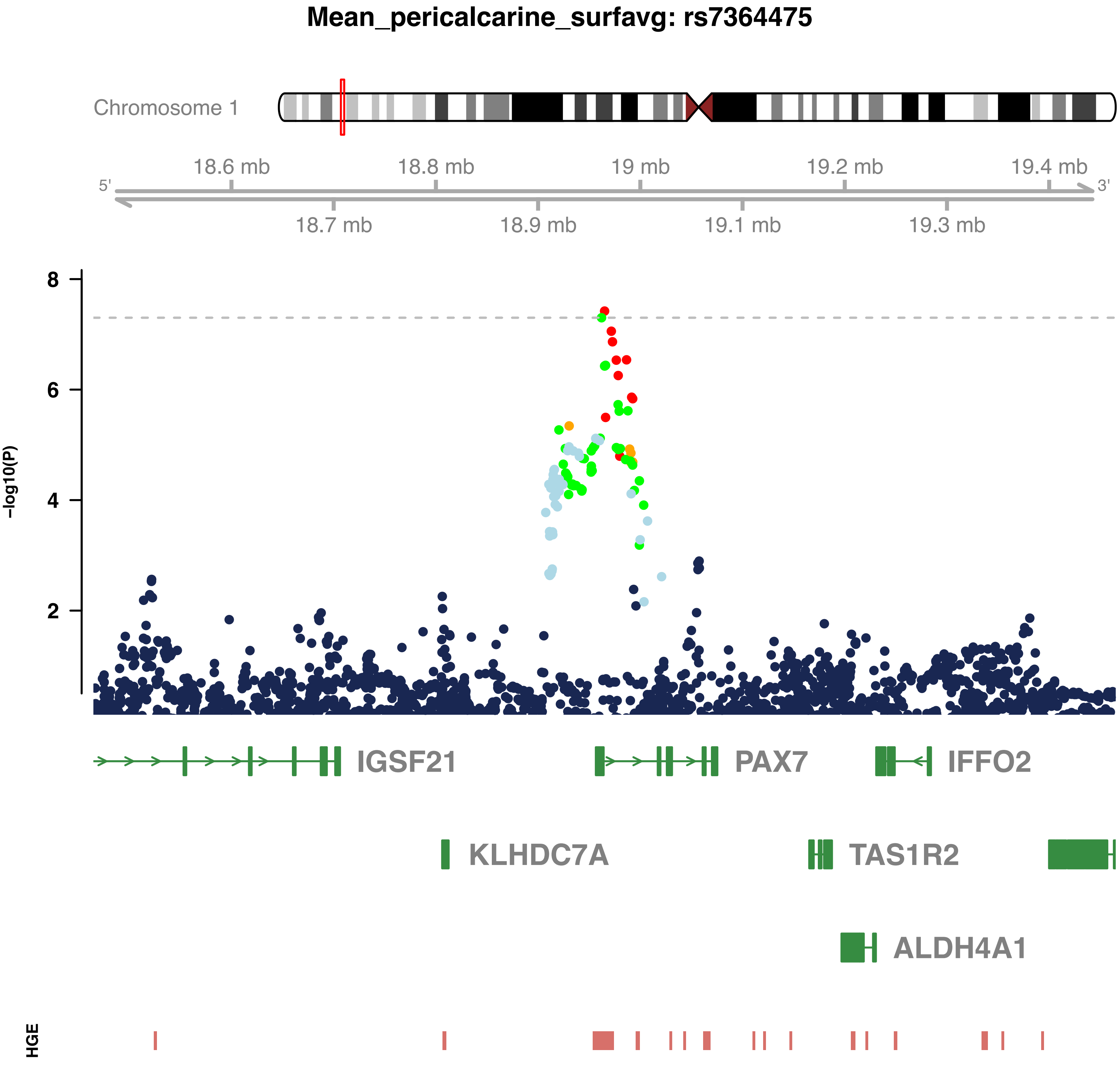
Mean_pericalcarine_surfavg: rs7364475
Chromosome 1
18.6 mb
18.8 mb
19 mb
19.2 mb
19.4 mb
5'
3'
18.7 mb
18.9 mb
19.1 mb
19.3 mb
8
6
4
2
-log10(P)
IGSF21
PAX7
IFFO2
KLHDC7A
TAS1R2
ALDH4A1
HGE

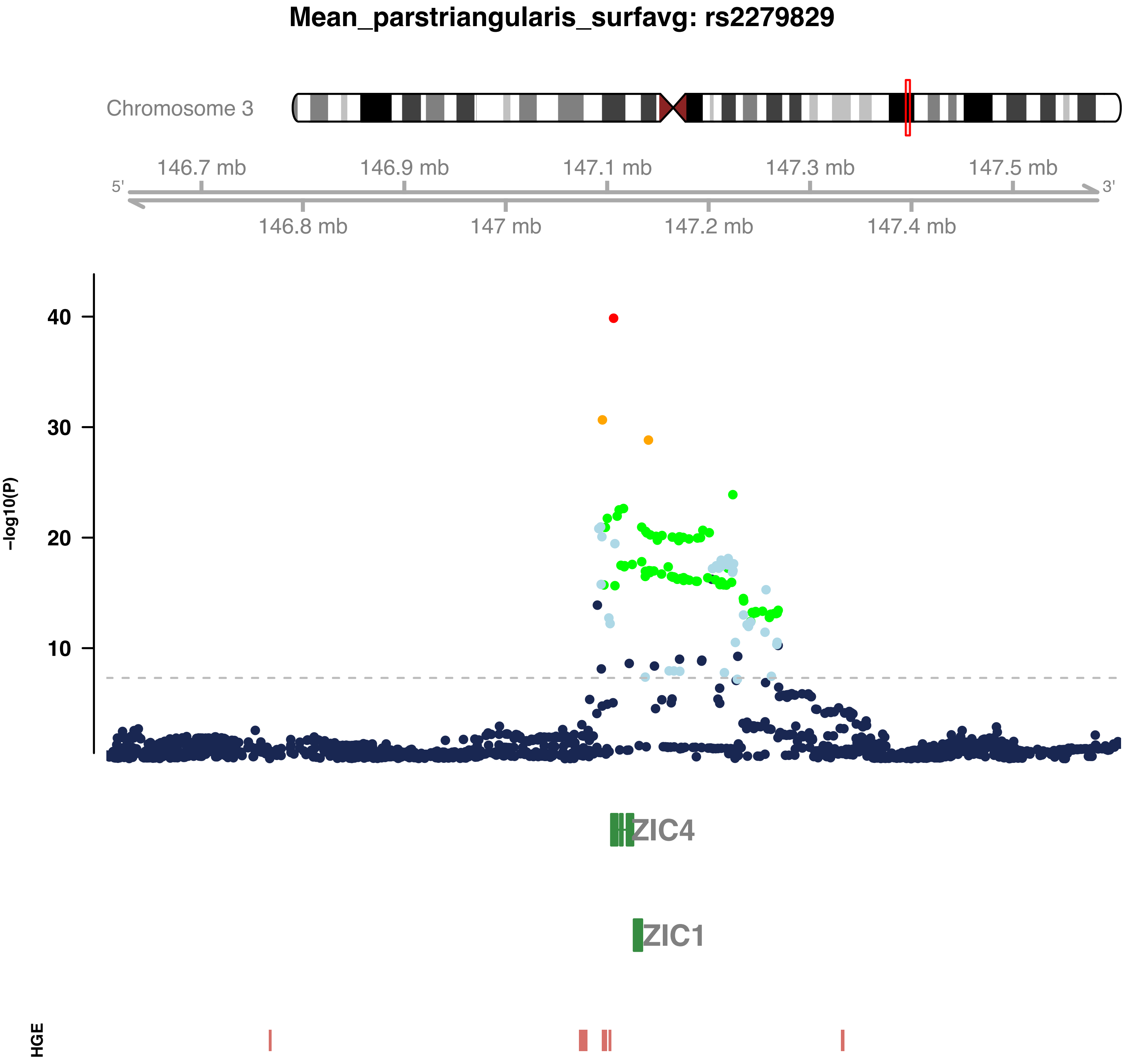

Mean_parstriangularis_surfavg: rs2279829
Chromosome 3
146.7 mb
146.9 mb
147.1 mb
147.3 mb
147.5 mb
5'
3'
146.8 mb
147 mb
147.2 mb
147.4 mb
-log10(P)
40
30
20
10
ZIC4
ZIC1
HGE

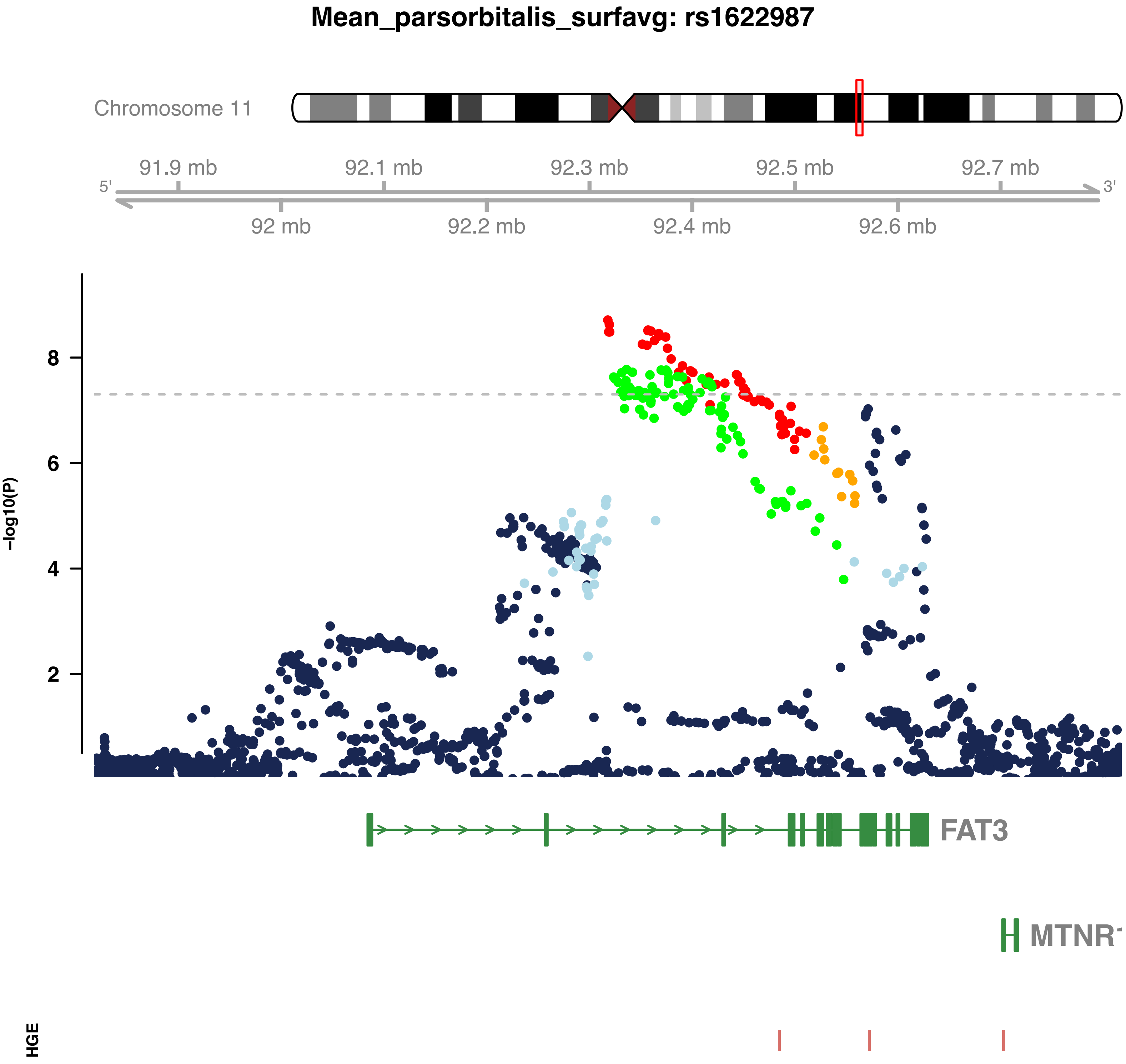

Mean_parsorbitalis_surfavg: rs1622987
Chromosome 11
91.9 mb
92.1 mb
92.3 mb
92.5 mb
92.7 mb
5'
3'
92 mb
92.2 mb
92.4 mb
92.6 mb
8
6
4
2
-log10(P)
FAT3
MTNR1
HGE

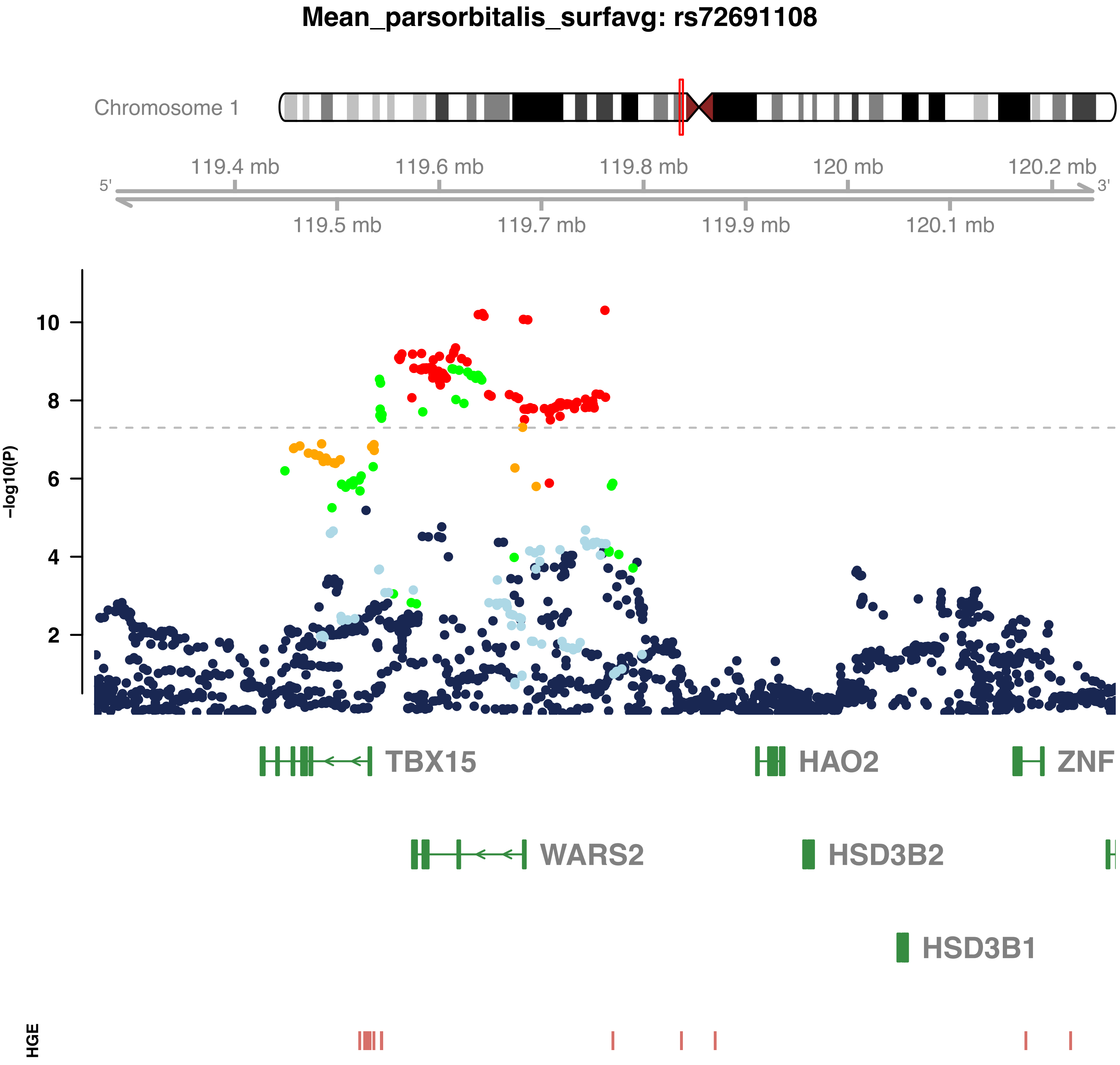

Mean_parsorbitalis_surfavg: rs72691108
Chromosome 1
119.4 mb
119.6 mb
119.8 mb
120 mb
120.2 mb
5'
3'
119.5 mb
119.7 mb
119.9 mb
120.1 mb
-log10(P)
10
8
6
4
2
TBX15
HAO2
ZNF
WARS2
HSD3B2
HSD3B1
HGE

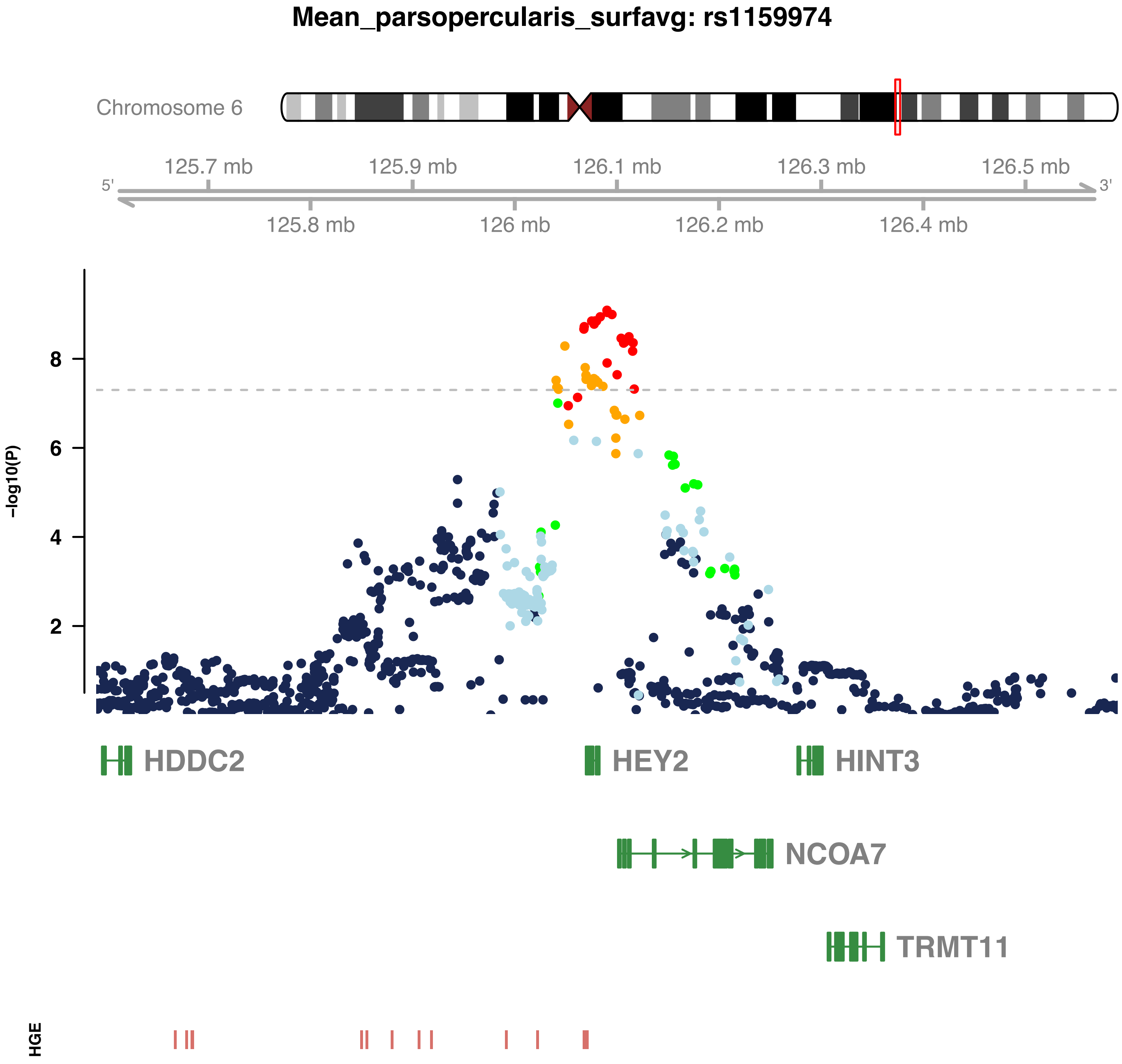

Mean_parsopercularis_surfavg: rs1159974
Chromosome 6
125.7 mb
125.9 mb
126.1 mb
126.3 mb
126.5 mb
5'
3'
125.8 mb
126 mb
126.2 mb
126.4 mb
−log10(P)
8
6
4
2
HDDC2
HEY2
HINT3
NCOA7
TRMT11
HGE

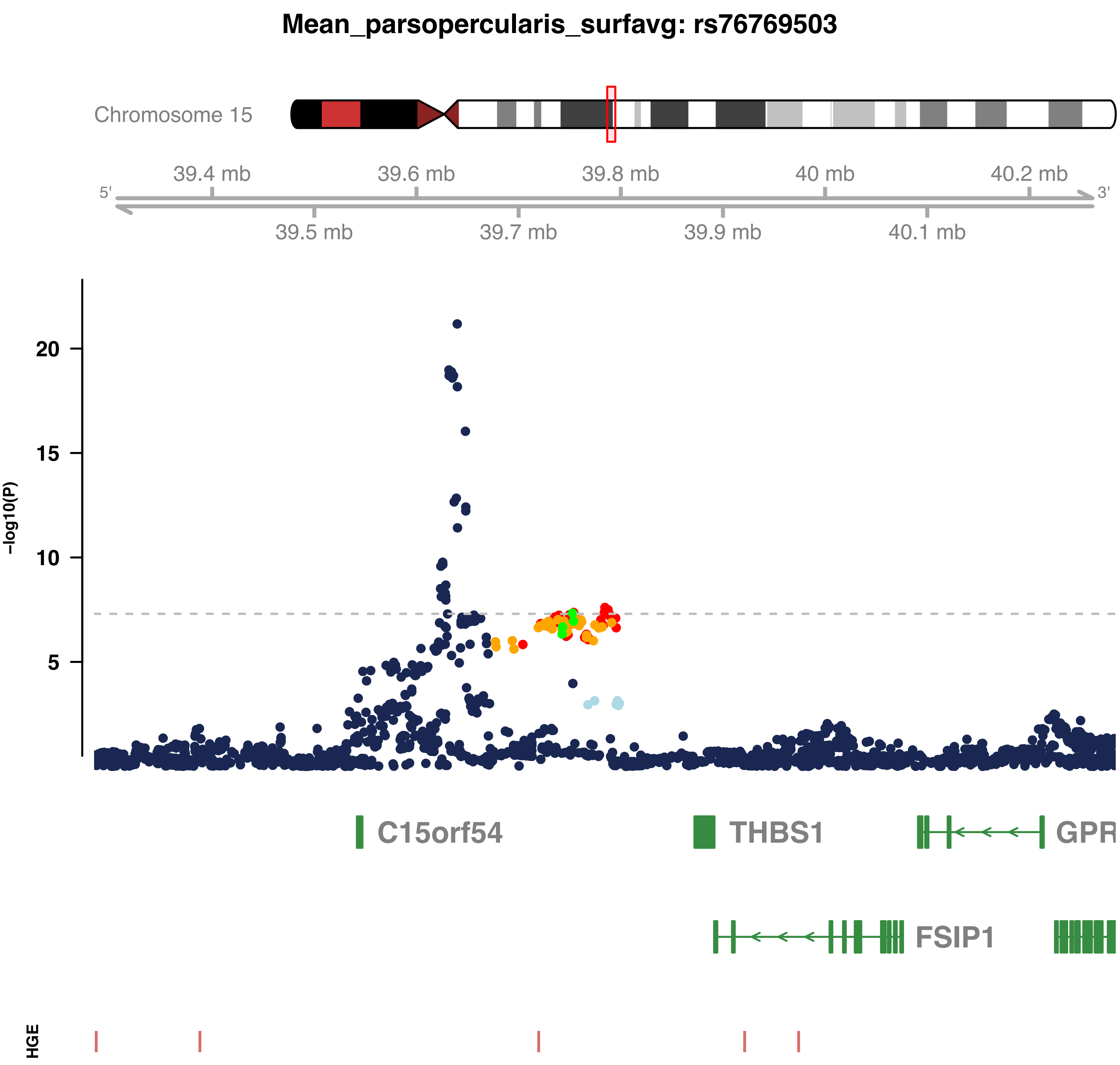

Mean_parsopercularis_surfavg: rs76769503
Chromosome 15
39.4 mb
39.6 mb
39.8 mb
40 mb
40.2 mb
5'
3'
39.5 mb
39.7 mb
39.9 mb
40.1 mb
−log10(P)
20
15
10
5
C15orf54
THBS1
GPR
FSIP1
HGE

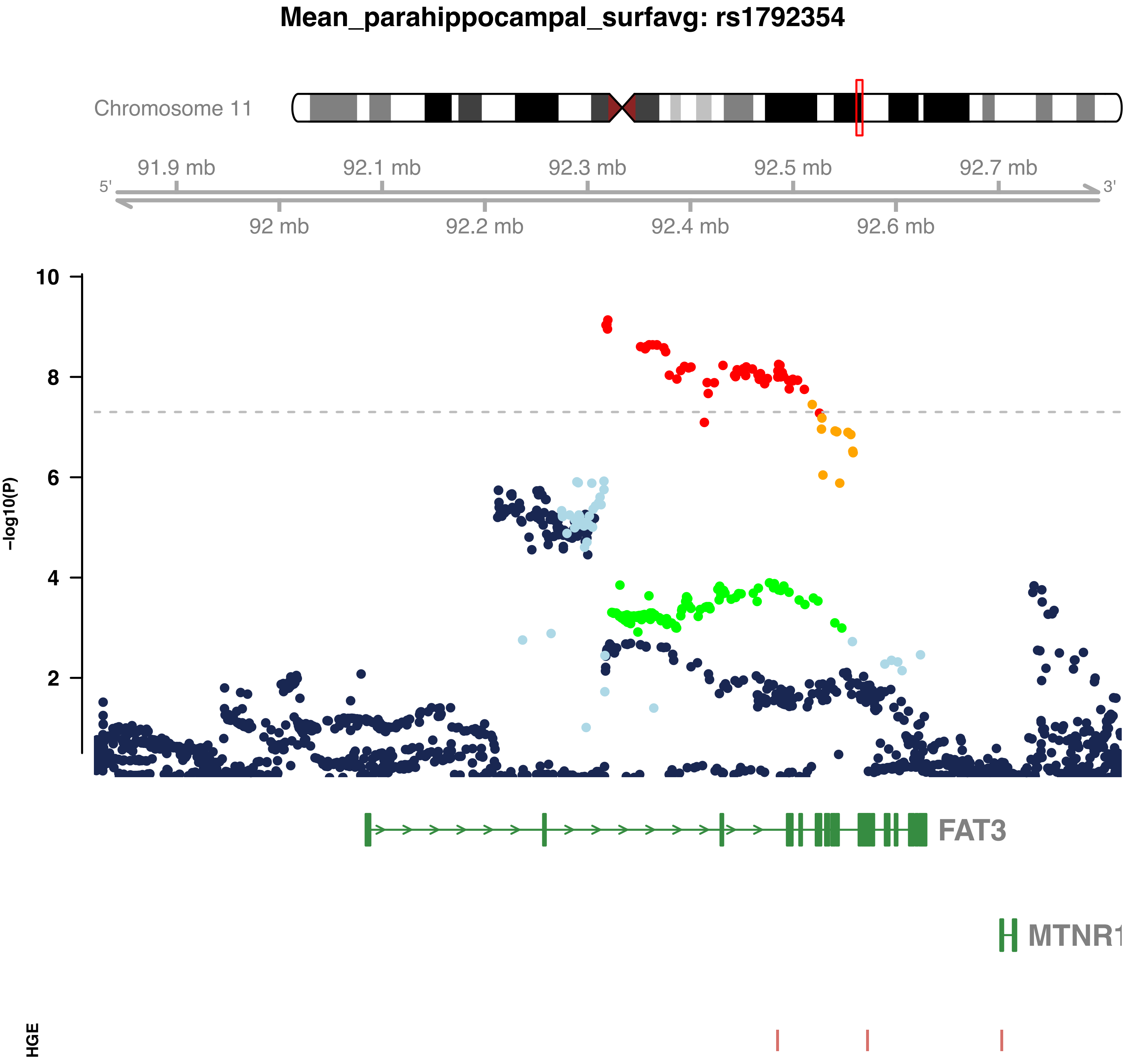

Mean_parahippocampal_surfavg: rs1792354
Chromosome 11
91.9 mb
92.1 mb
92.3 mb
92.5 mb
92.7 mb
5'
3'
92 mb
92.2 mb
92.4 mb
92.6 mb
10
8
6
4
2
-log10(P)
FAT3
MTNR1
HGE

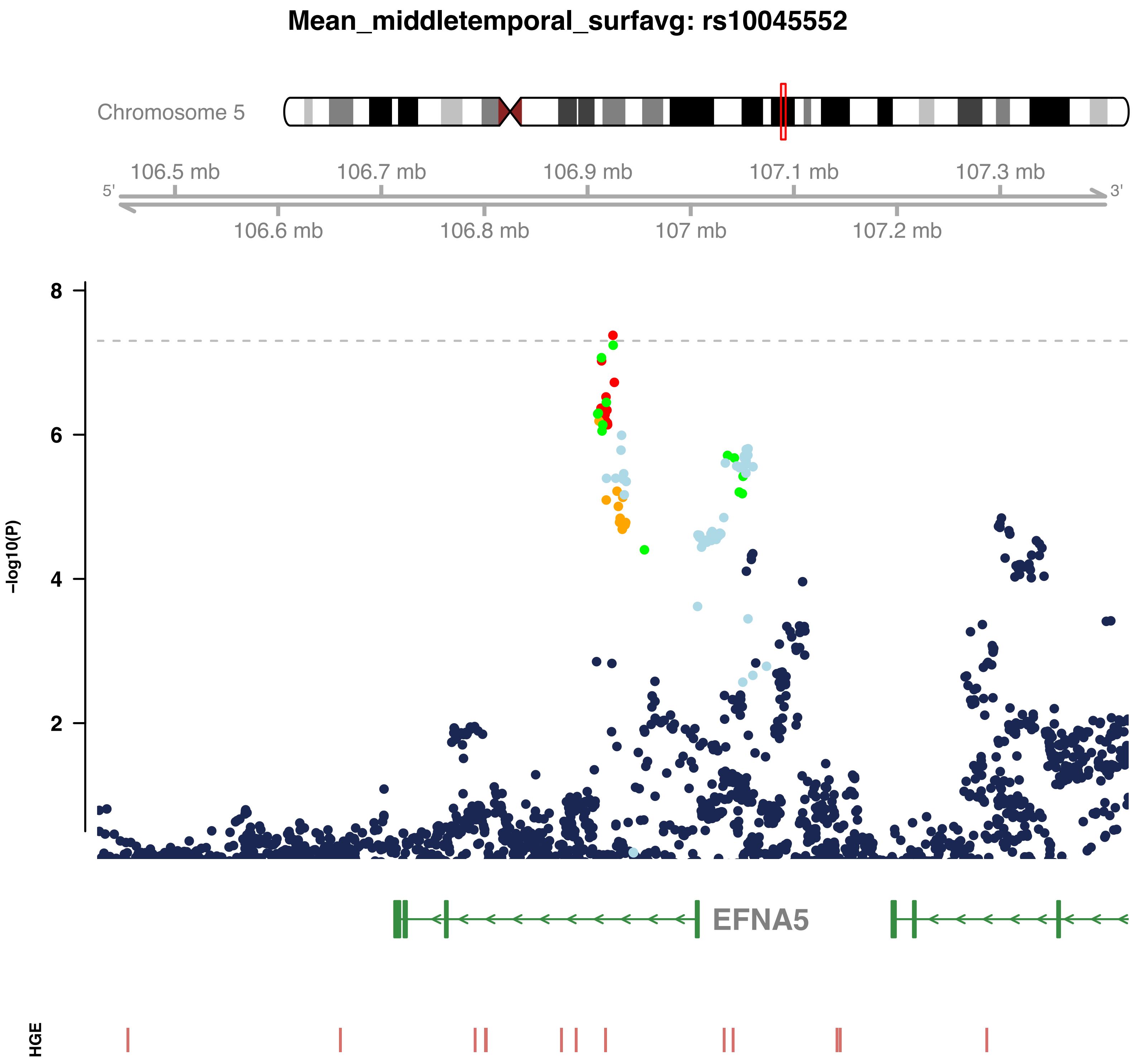

Mean_middletemporal_surfavg: rs10045552
Chromosome 5
106.5 mb
106.7 mb
106.9 mb
107.1 mb
107.3 mb
5'
3'
106.6 mb
106.8 mb
107 mb
107.2 mb
8
6
4
2
-log10(P)
EFNA5
HGE

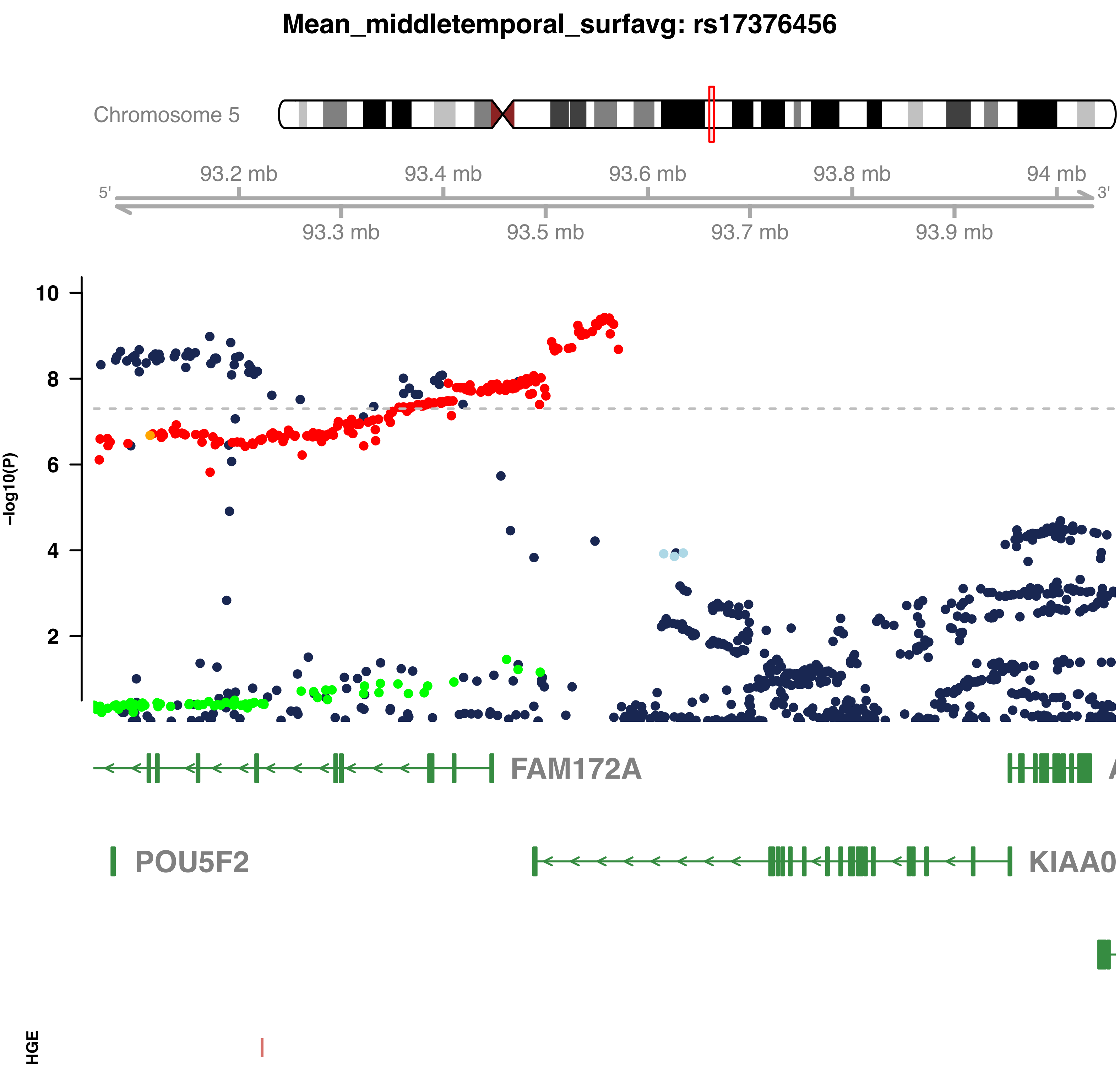
Mean_middletemporal_surfavg: rs17376456
Chromosome 5
93.2 mb
93.4 mb
93.6 mb
93.8 mb
94 mb
5'
3'
93.3 mb
93.5 mb
93.7 mb
93.9 mb
10
8
6
4
2
−log10(P)
FAM172A
POU5F2
KIAA0
HGE

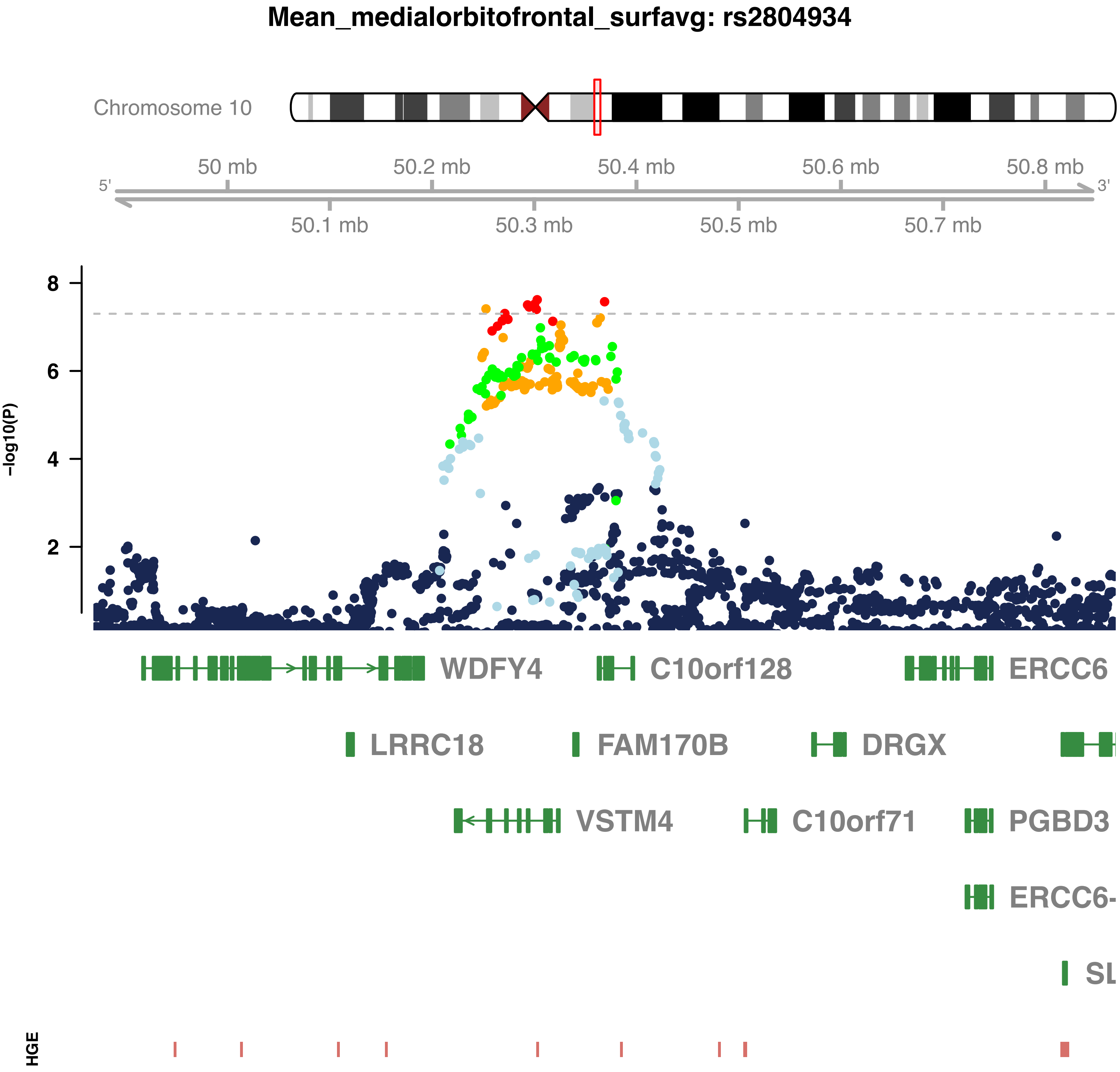

Mean_medialorbitofrontal_surfavg: rs2804934
Chromosome 10
50 mb
50.2 mb
50.4 mb
50.6 mb
50.8 mb
5'
3'
50.1 mb
50.3 mb
50.5 mb
50.7 mb
−log10(P)
8
6
4
2
WDFY4
C10orf128
ERCC6
LRRC18
FAM170B
DRGX
VSTM4
C10orf71
PGBD3
ERCC6-
SL
HGE

Mean_lingual_surfavg: rs28410513

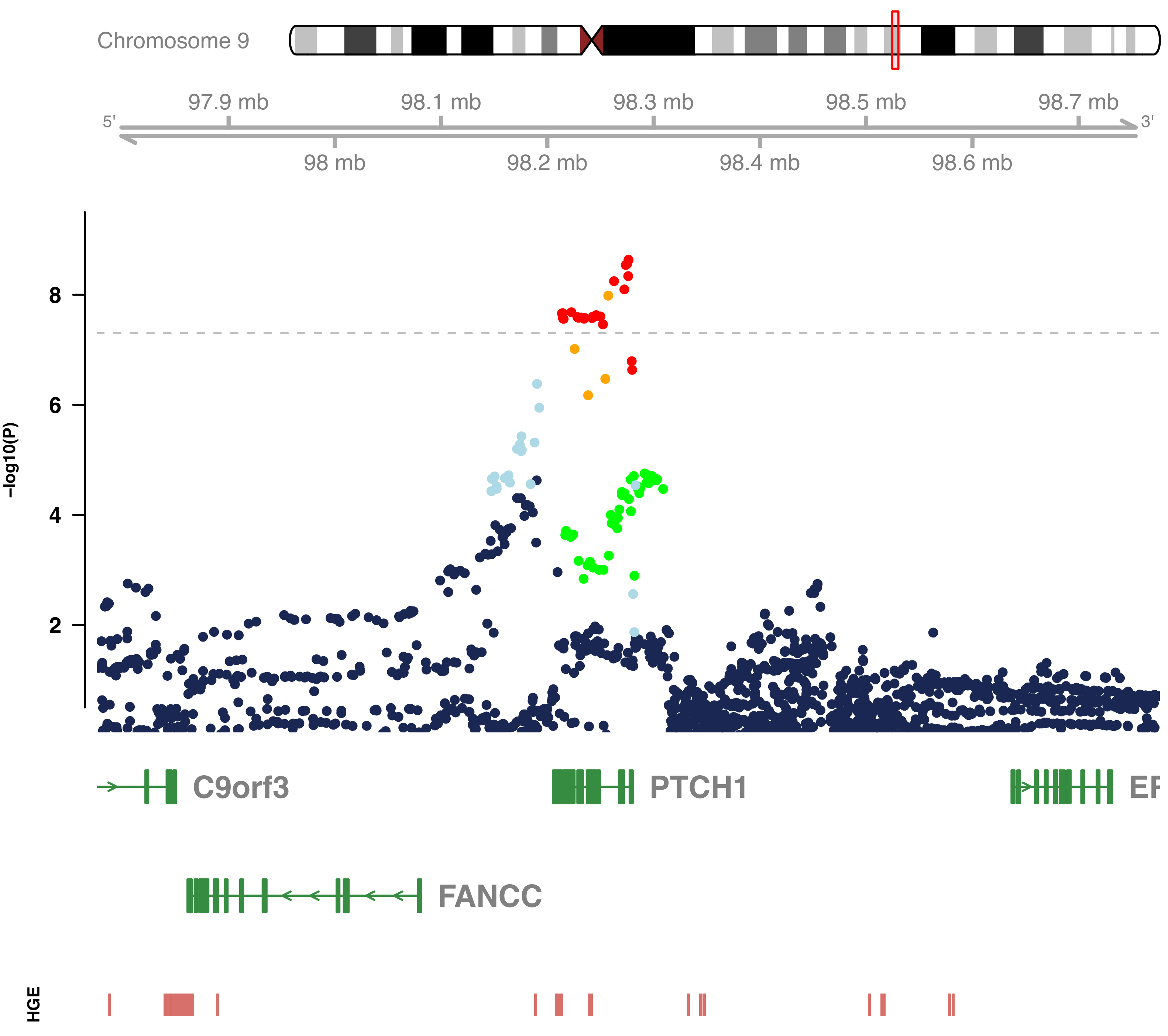

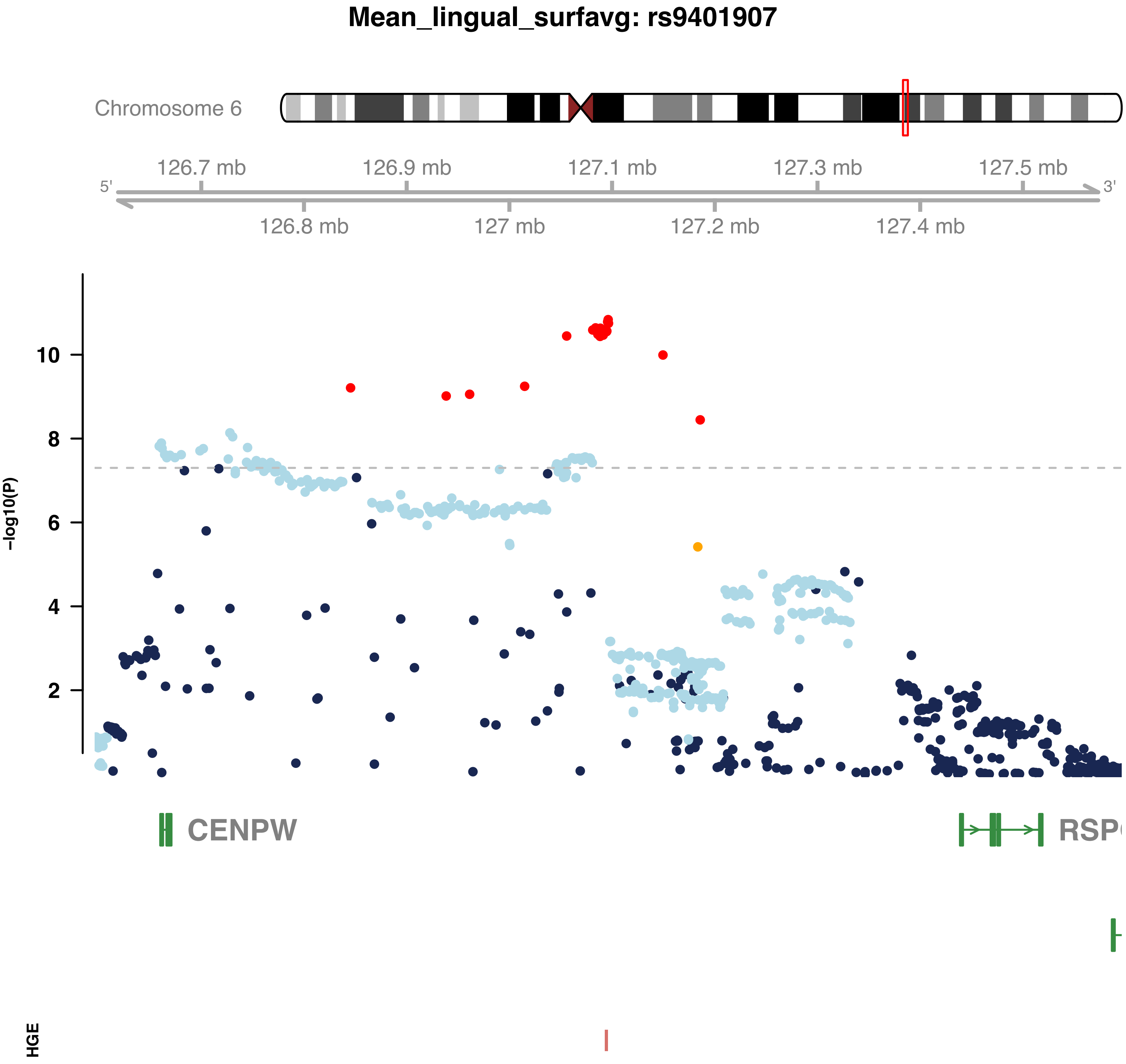
Mean_lingual_surfavg: rs9401907
Chromosome 6
126.7 mb
126.9 mb
127.1 mb
127.3 mb
127.5 mb
5'
3'
126.8 mb
127 mb
127.2 mb
127.4 mb
−log10(P)
10
8
6
4
2
CENPW
RSP
HGE

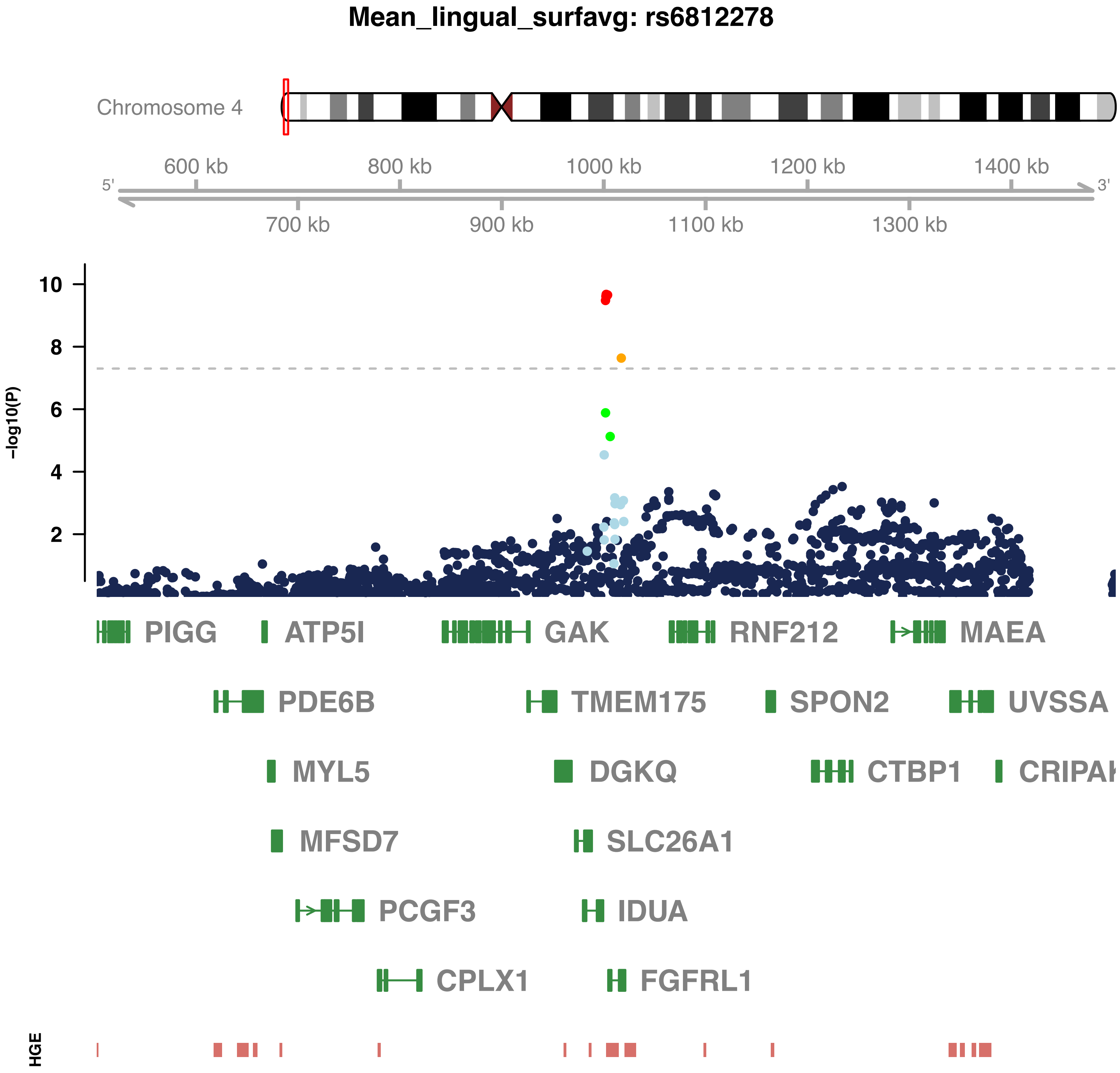

Mean_lingual_surfavg: rs6812278
Chromosome 4
600 kb
800 kb
1000 kb
1200 kb
1400 kb
5'
3'
700 kb
900 kb
1100 kb
1300 kb
-log10(P)
10
8
6
4
2
PIGG
ATP5I
GAK
RNF212
MAEA
PDE6B
TMEM175
SPON2
UVSSA
MYL5
DGKQ
CTBP1
MFSD7
SLC26A1
PCGF3
IDUA
CPLX1
FGFRL1
HGE

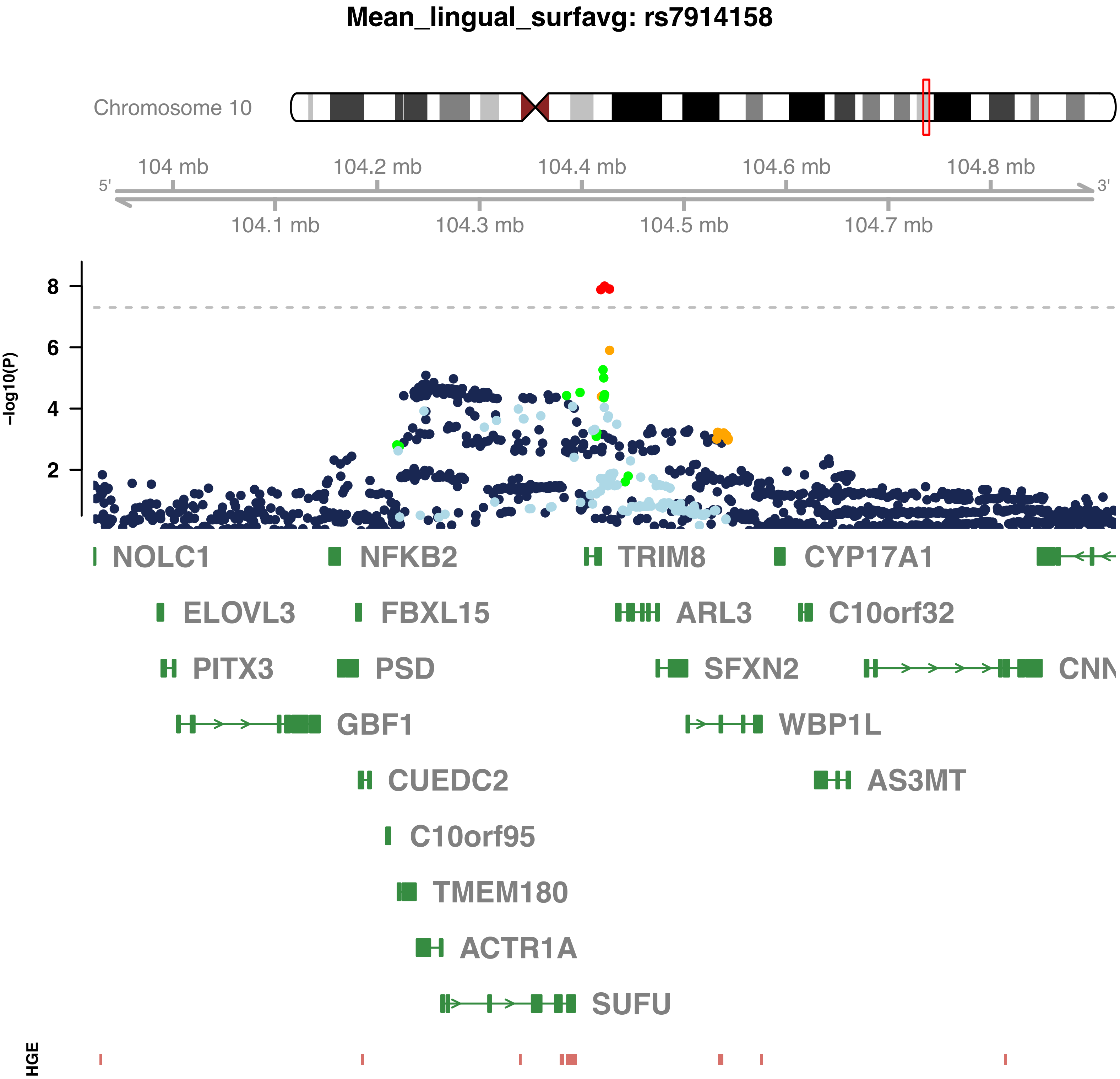

Mean_lingual_surfavg: rs7914158
Chromosome 10
104 mb
104.2 mb
104.4 mb
104.6 mb
104.8 mb
5'
3'
104.1 mb
104.3 mb
104.5 mb
104.7 mb
−log10(P)
8
6
4
2
NOLC1
NFKB2
TRIM8
CYP17A1
ELOVL3
FBXL15
ARL3
C10orf32
PITX3
PSD
SFXN2
CNN
GBF1
WBP1L
CUEDC2
AS3MT
C10orf95
TMEM180
ACTR1A
SUFU
HGE

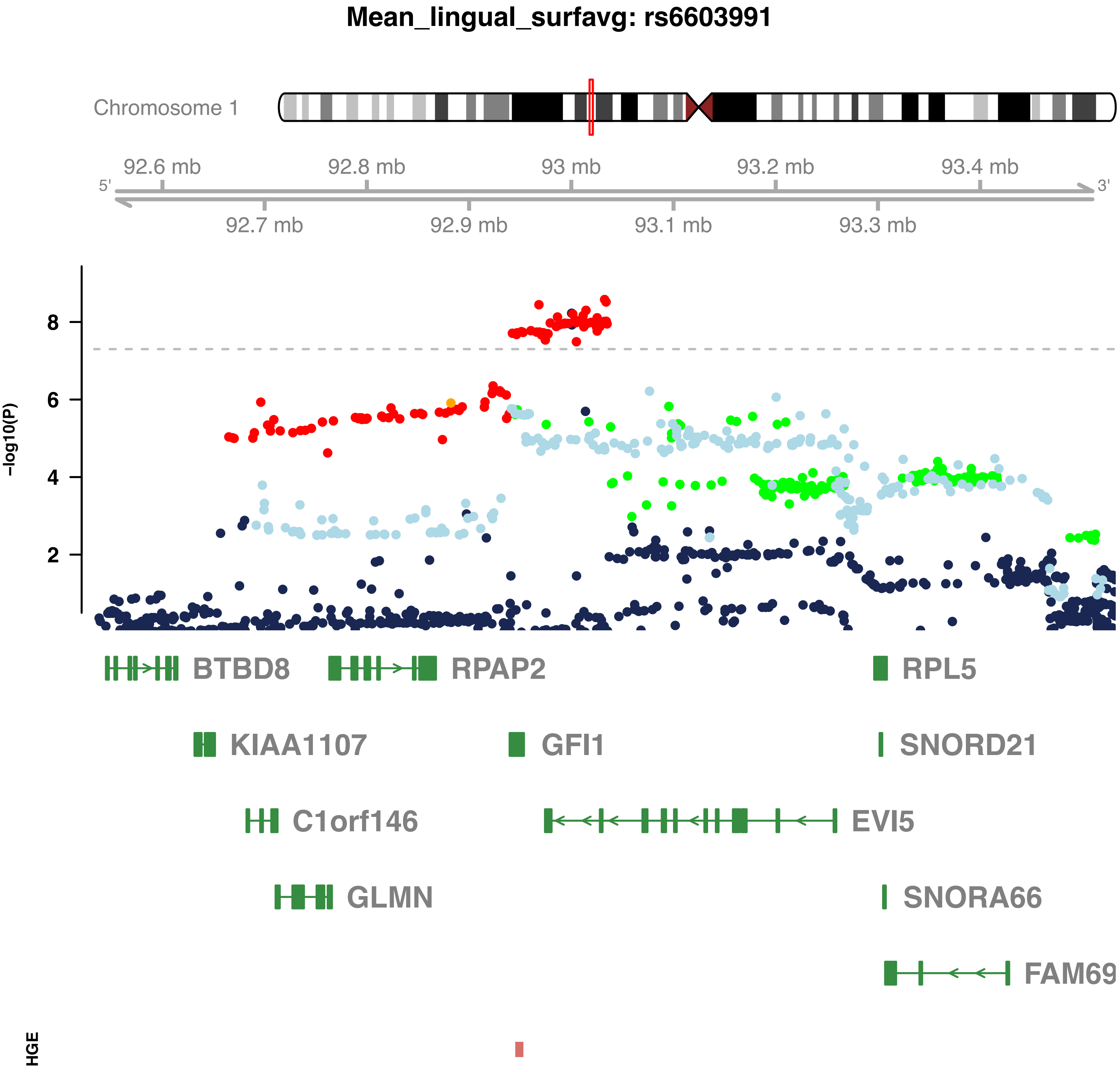

Mean_lingual_surfavg: rs6603991
Chromosome 1
92.6 mb
92.8 mb
93 mb
93.2 mb
93.4 mb
5'
3'
92.7 mb
92.9 mb
93.1 mb
93.3 mb
−log10(P)
8
6
4
2
BTBD8
RPAP2
RPL5
KIAA1107
GFI1
SNORD21
C1orf146
EVI5
GLMN
SNORA66
FAM69
HGE

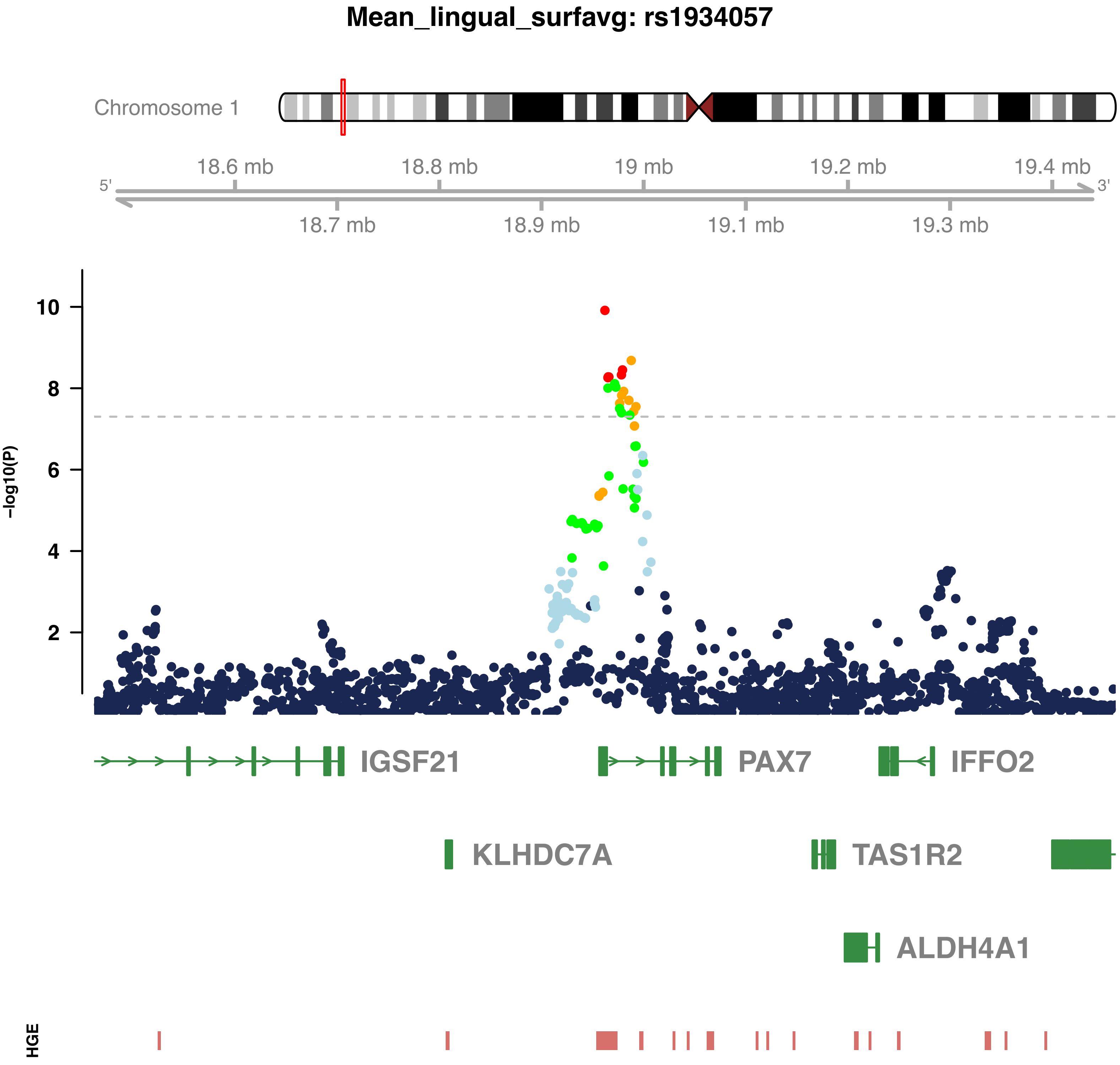

Mean_lingual_surfavg: rs1934057
Chromosome 1
18.6 mb
18.8 mb
19 mb
19.2 mb
19.4 mb
5'
3'
18.7 mb
18.9 mb
19.1 mb
19.3 mb
-log10(P)
10
8
6
4
2
IGSF21
PAX7
IFFO2
KLHDC7A
TAS1R2
ALDH4A1
HGE

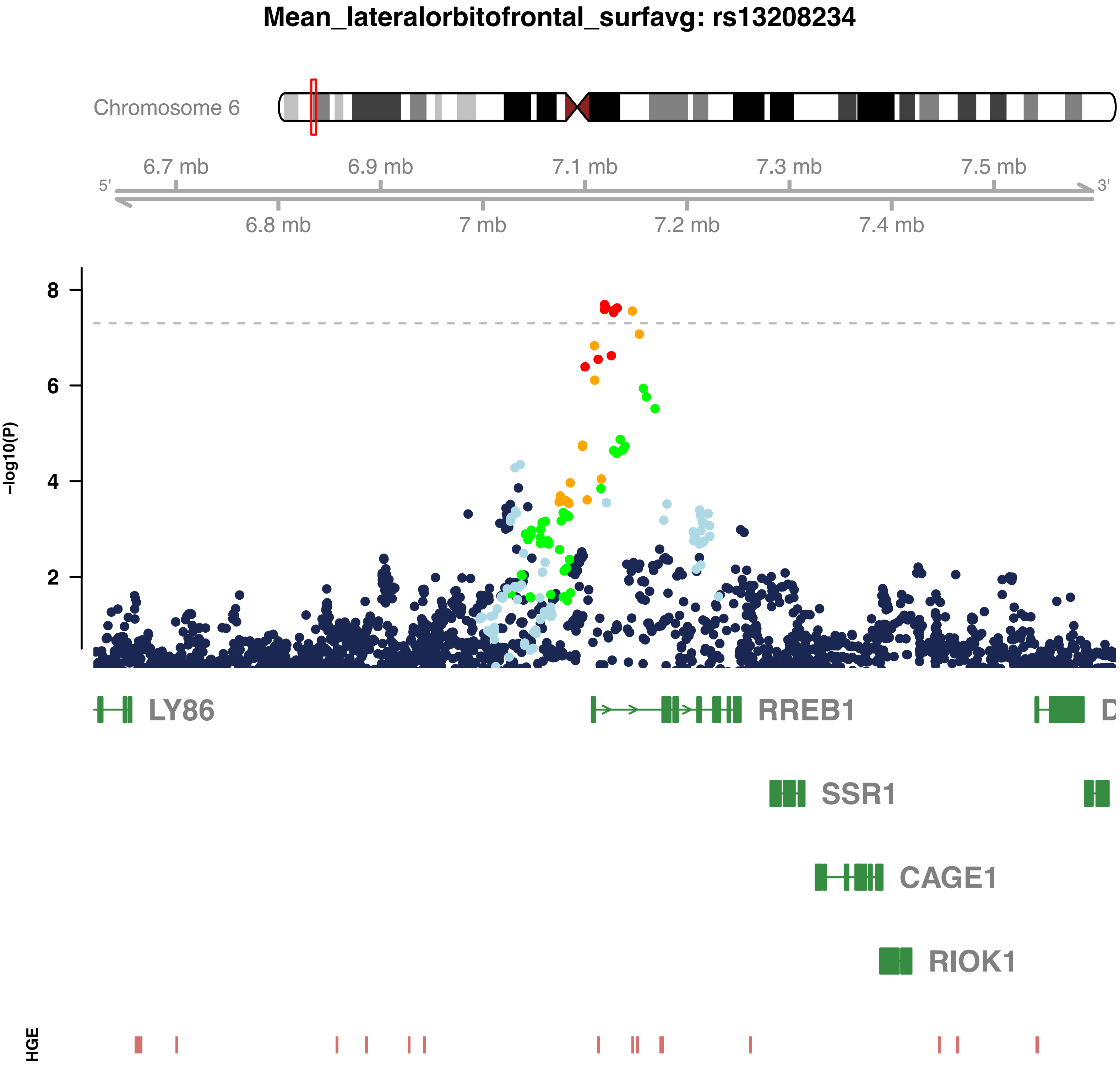

Mean_lateralorbitofrontal_surfavg: rs13208234
Chromosome 6
6.7 mb
6.9 mb
7.1 mb
7.3 mb
7.5 mb
5'
3'
6.8 mb
7 mb
7.2 mb
7.4 mb
-log10(P)
8
6
4
2
LY86
RREB1
SSR1
CAGE1
RIOK1
HGE

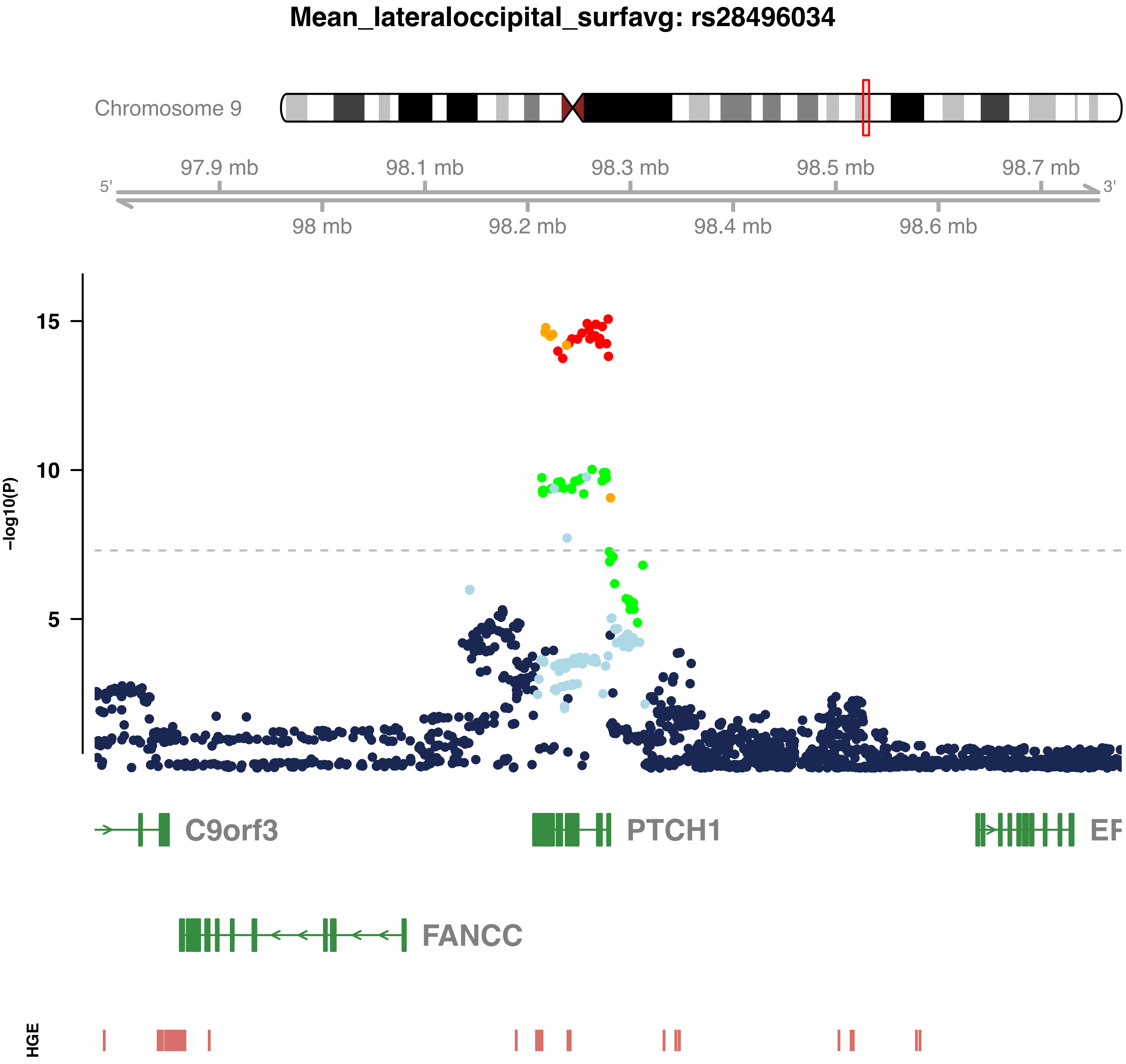

Mean_lateraloccipital_surfavg: rs28496034
Chromosome 9
97.9 mb
98.1 mb
98.3 mb
98.5 mb
98.7 mb
5'
3'
98 mb
98.2 mb
98.4 mb
98.6 mb
15
10
5
-log10(P)
C9orf3
PTCH1
FANCC
HGE

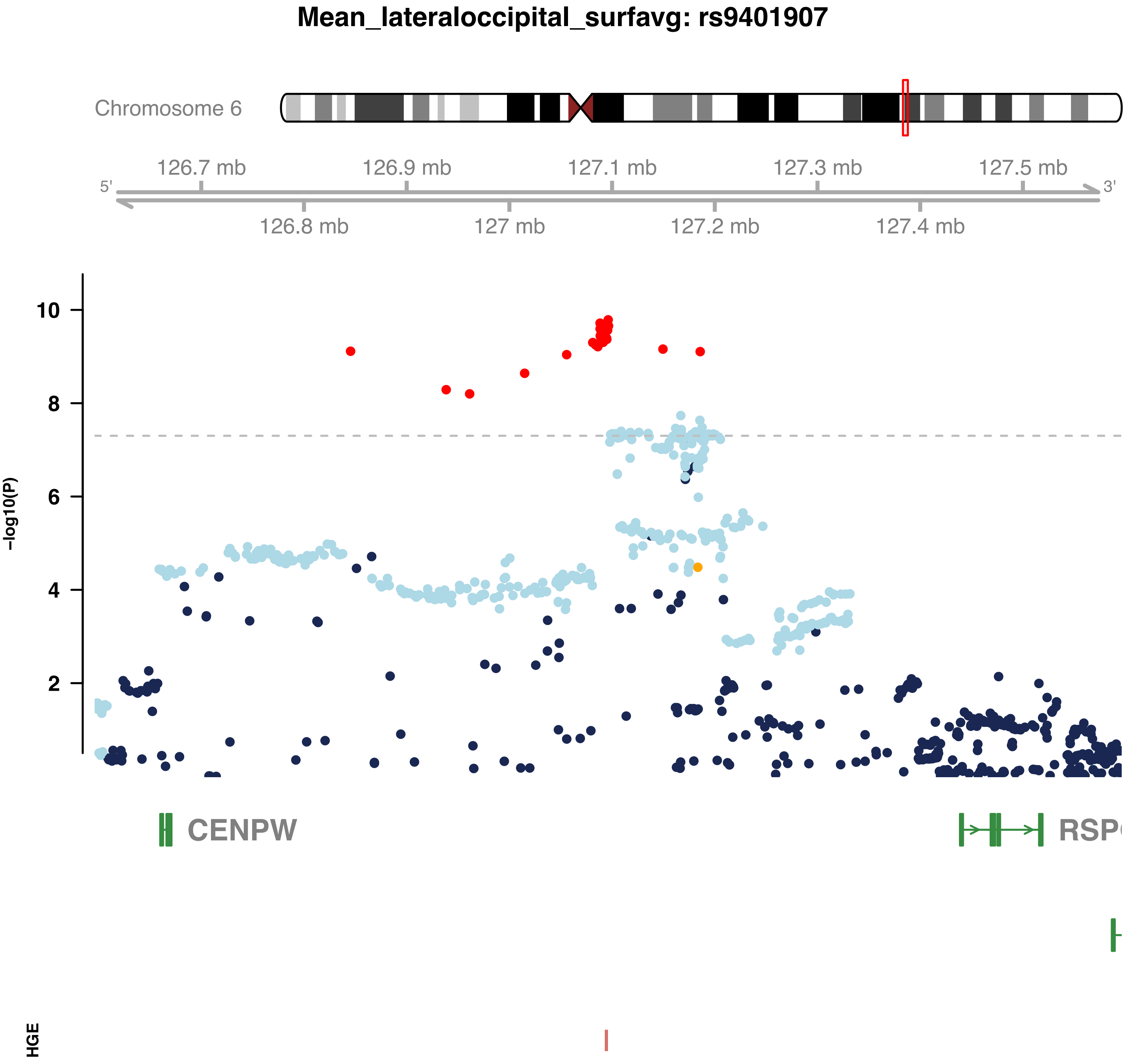
Mean_lateraloccipital_surfavg: rs9401907
Chromosome 6
126.7 mb
126.9 mb
127.1 mb
127.3 mb
127.5 mb
5'
3'
126.8 mb
127 mb
127.2 mb
127.4 mb
-log10(P)
10
8
6
4
2
CENPW
RSP
HGE

Mean_lateraloccipital_surfavg: rs56007616

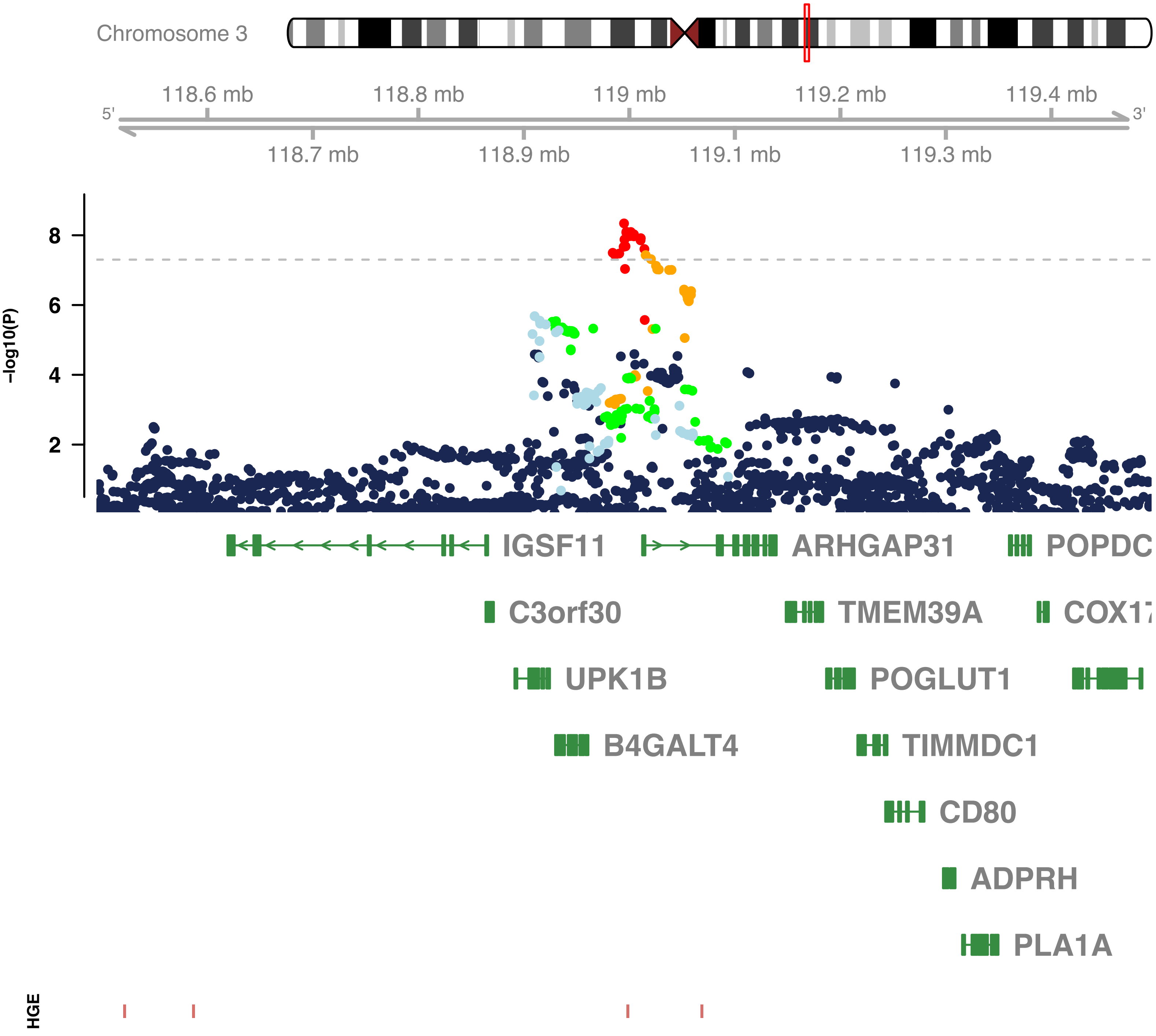

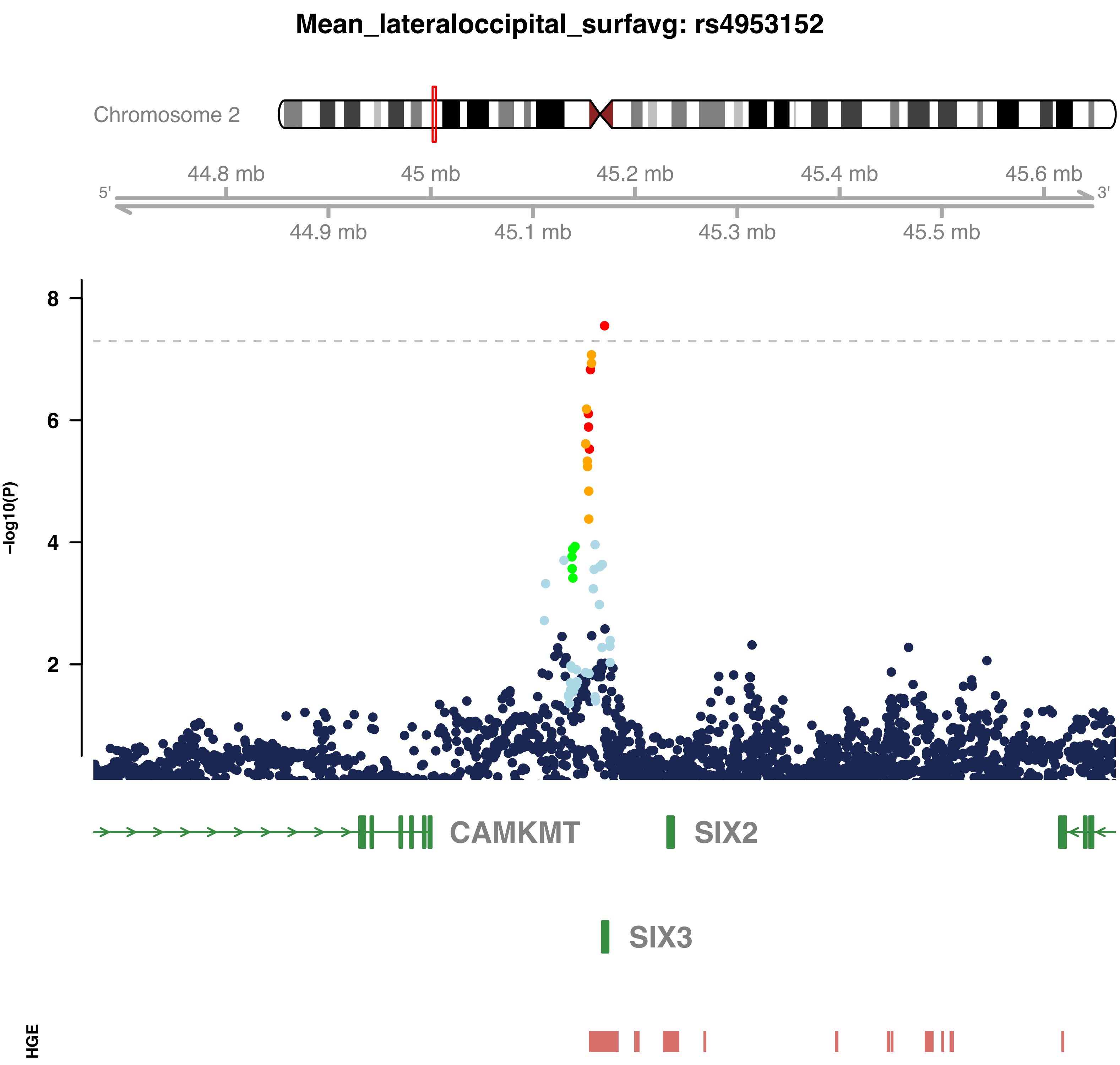

Mean_lateraloccipital_surfavg: rs4953152
Chromosome 2
44.8 mb
45 mb
45.2 mb
45.4 mb
45.6 mb
5'
3'
44.9 mb
45.1 mb
45.3 mb
45.5 mb
8
6
4
2
−log10(P)
CAMKMT
SIX2
SIX3
HGE

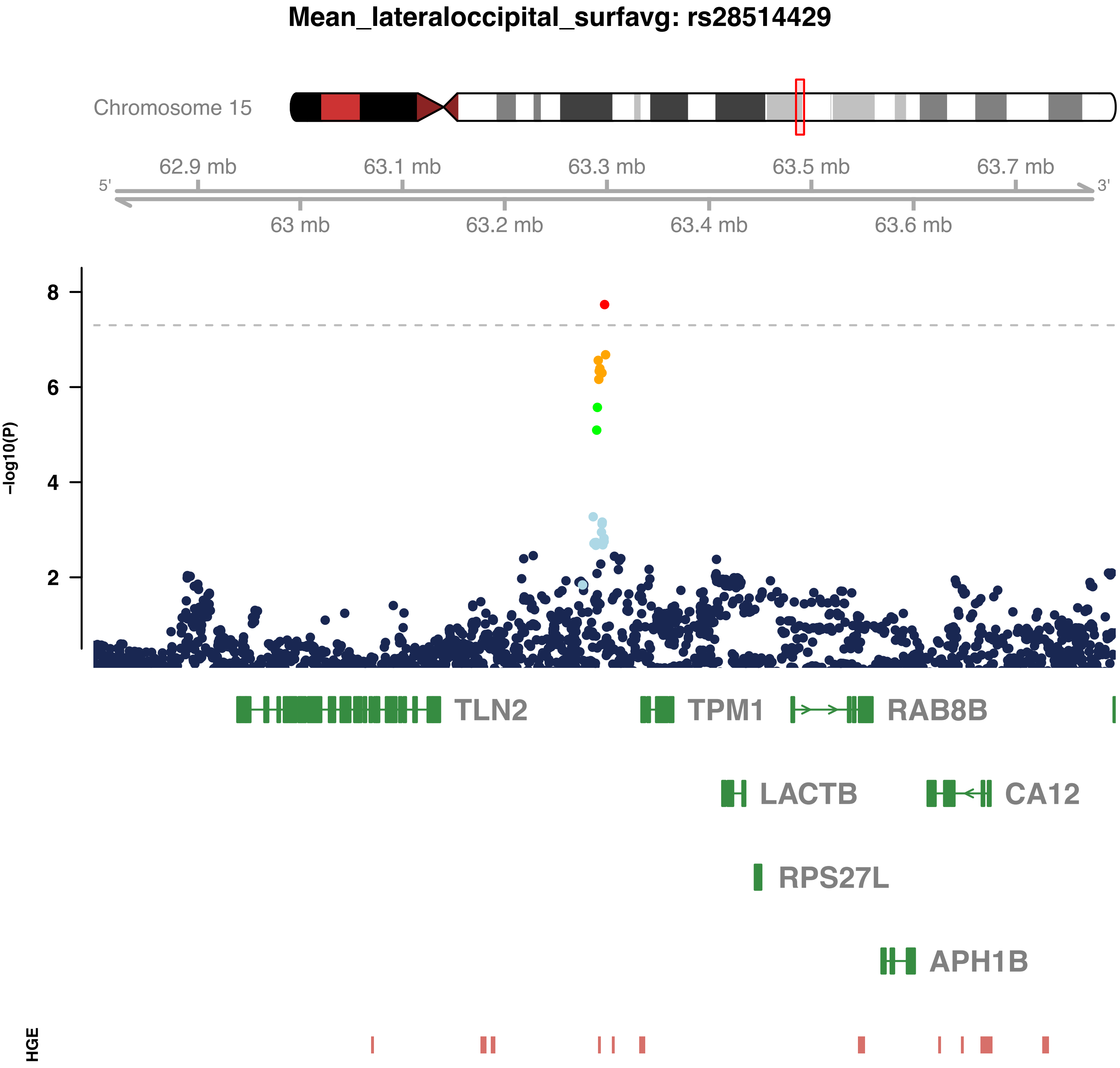

Mean_lateraloccipital_surfavg: rs28514429
Chromosome 15
62.9 mb
63.1 mb
63.3 mb
63.5 mb
63.7 mb
5'
3'
63 mb
63.2 mb
63.4 mb
63.6 mb
8
6
4
2
−log10(P)
TLN2
TPM1
RAB8B
LACTB
CA12
RPS27L
APH1B
HGE

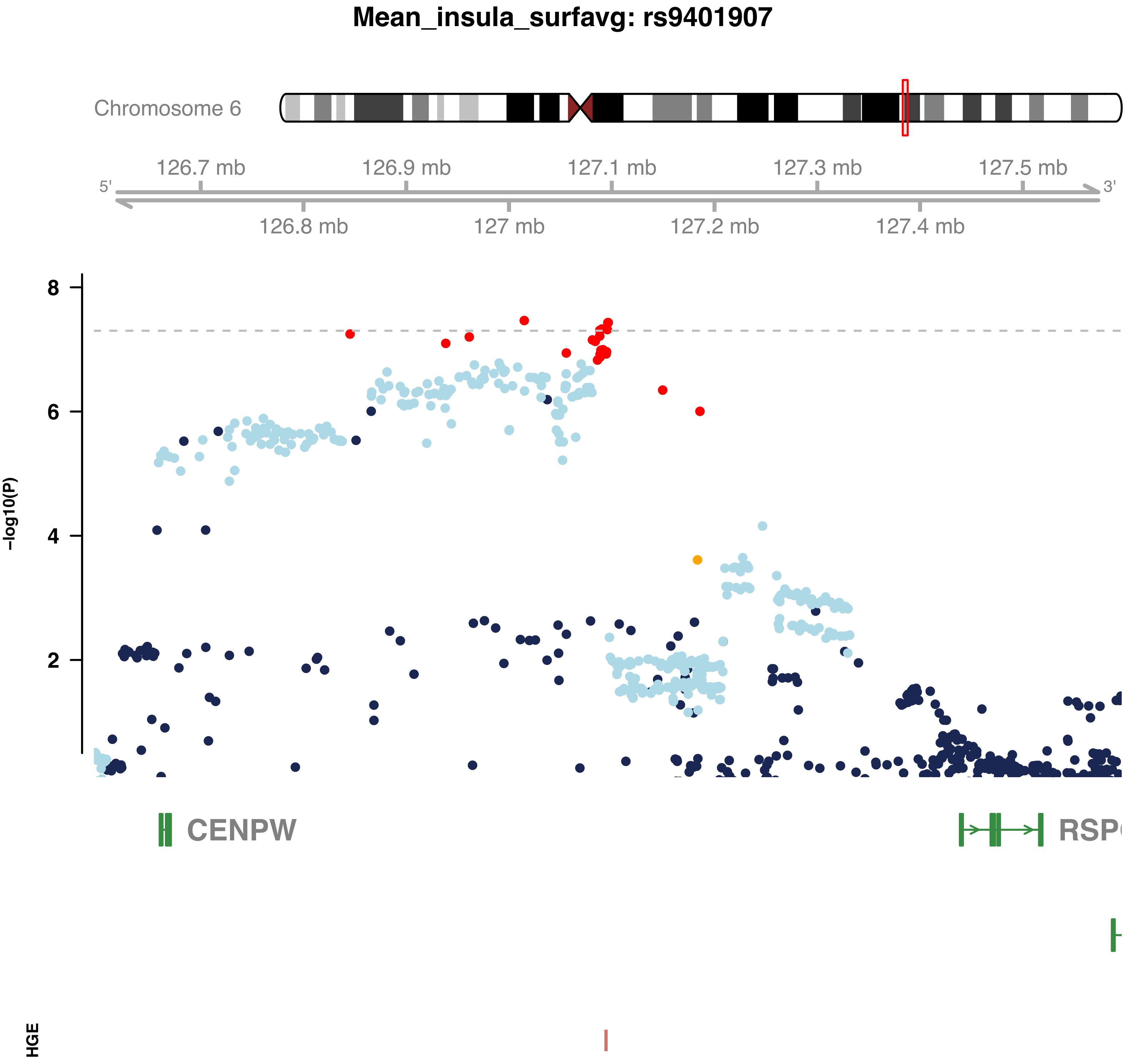

Mean_insula_surfavg: rs9401907
Chromosome 6
126.7 mb
126.9 mb
127.1 mb
127.3 mb
127.5 mb
5'
3'
126.8 mb
127 mb
127.2 mb
127.4 mb
8
6
4
2
−log10(P)
CENPW
RSP
HGE

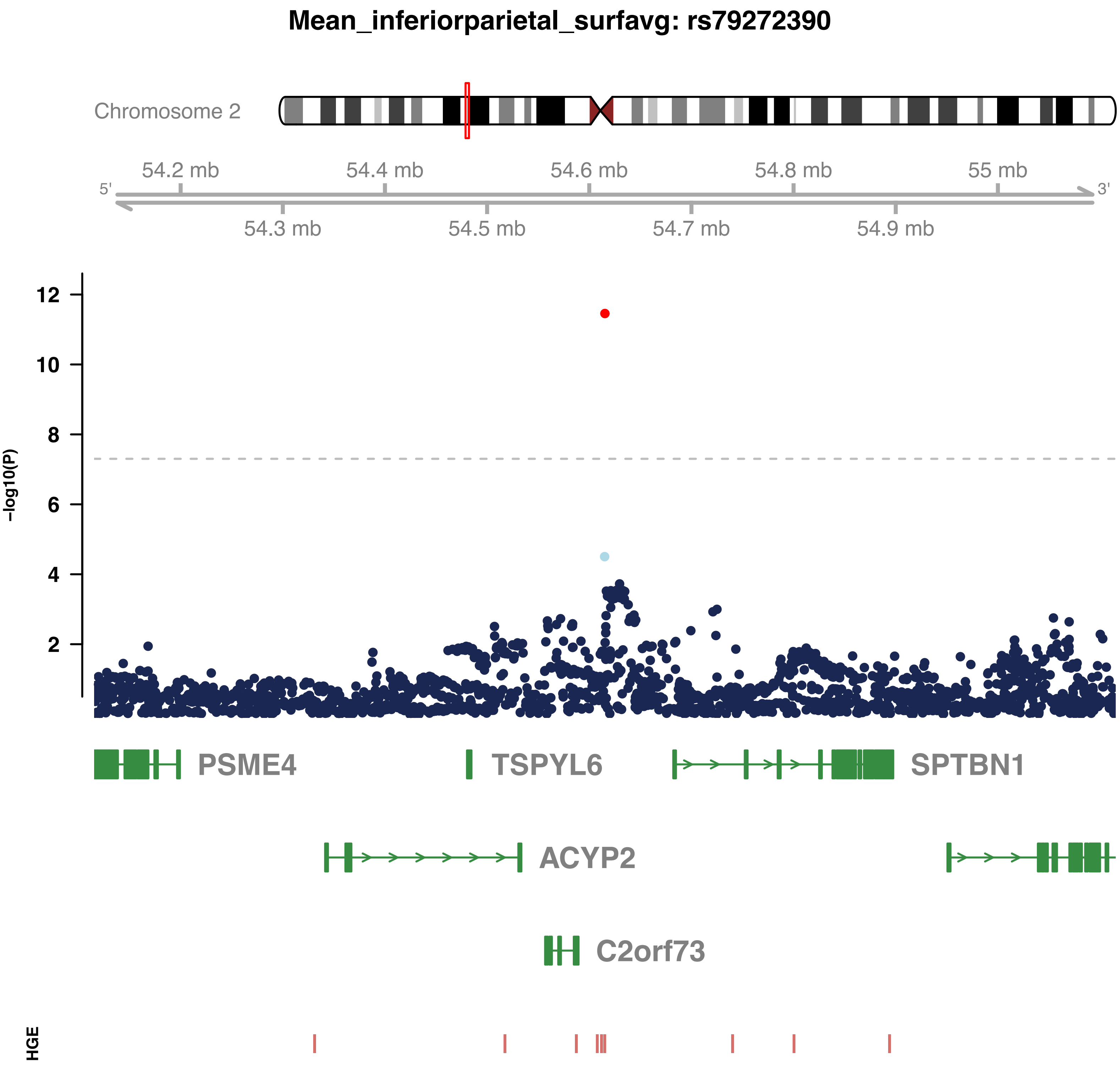
Mean_inferiorparietal_surfavg: rs79272390
Chromosome 2
54.2 mb
54.4 mb
54.6 mb
54.8 mb
55 mb
5'
3'
54.3 mb
54.5 mb
54.7 mb
54.9 mb
-log10(P)
12
10
8
6
4
2
PSME4
TSPYL6
SPTBN1
ACYP2
C2orf73
HGE

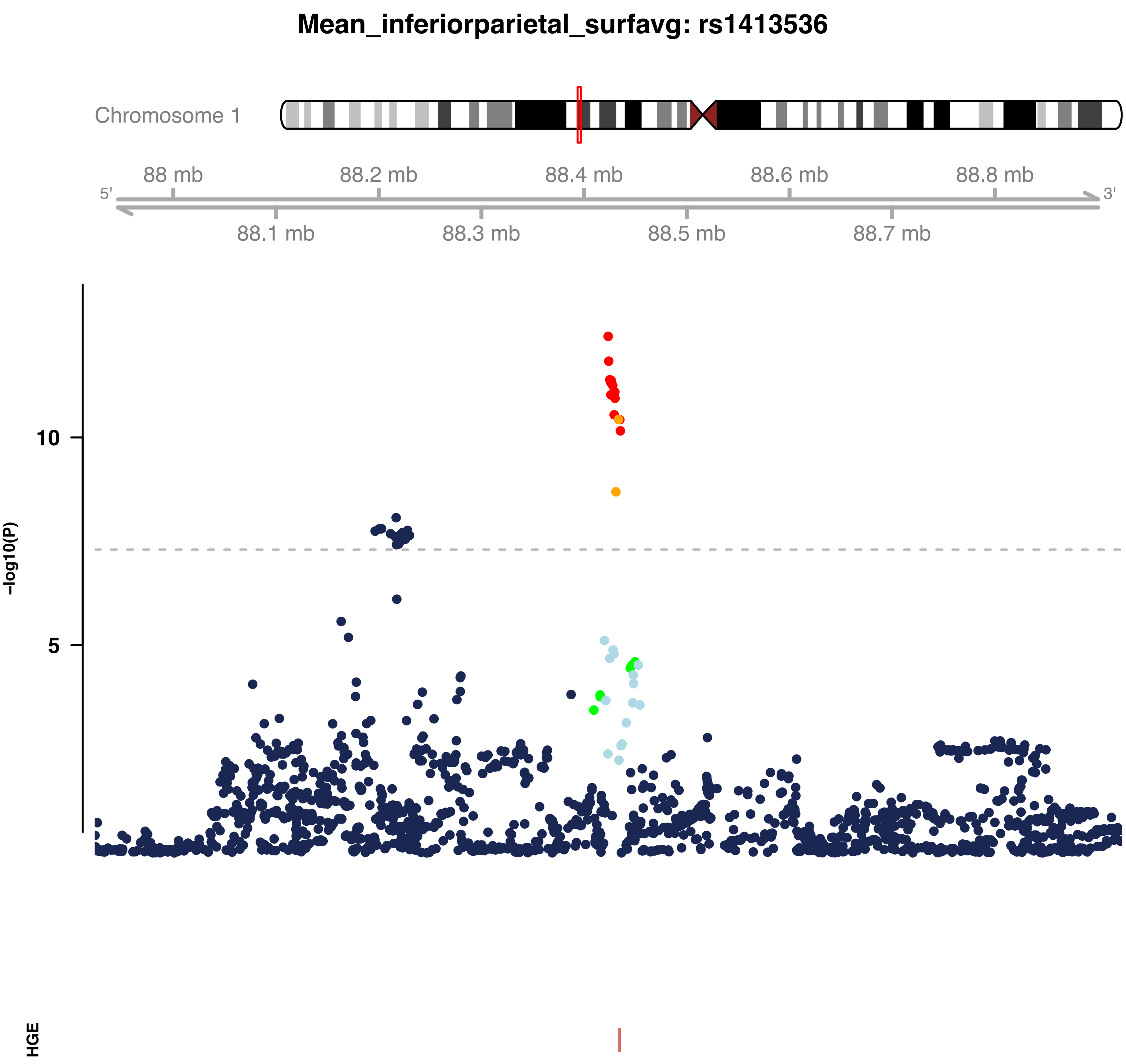

Mean_inferiorparietal_surfavg: rs1413536
Chromosome 1
88 mb
88.2 mb
88.4 mb
88.6 mb
88.8 mb
5'
3'
88.1 mb
88.3 mb
88.5 mb
88.7 mb
10
5
-log10(P)
HGE

Mean_fusiform_surfavg: rs2074404

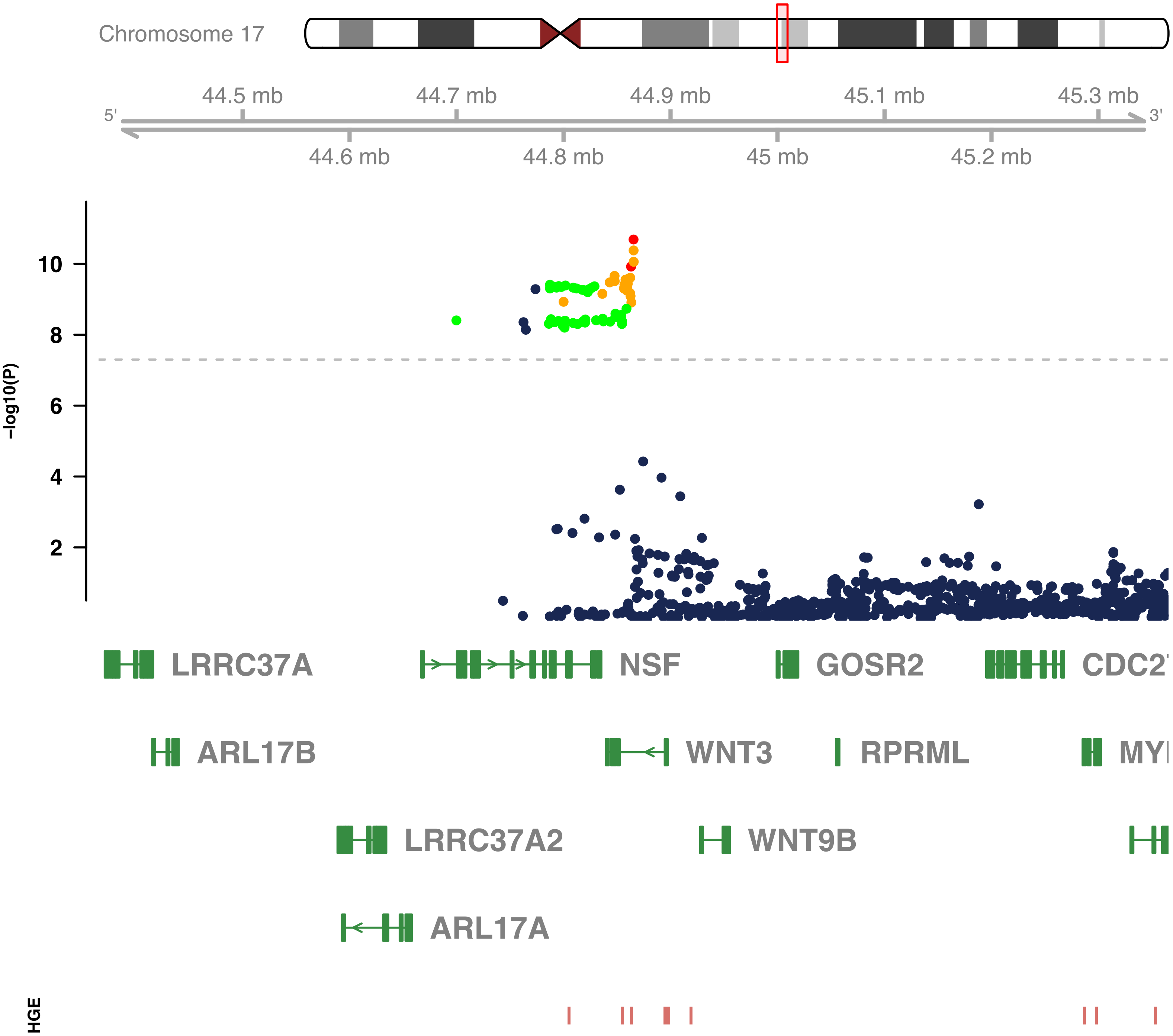

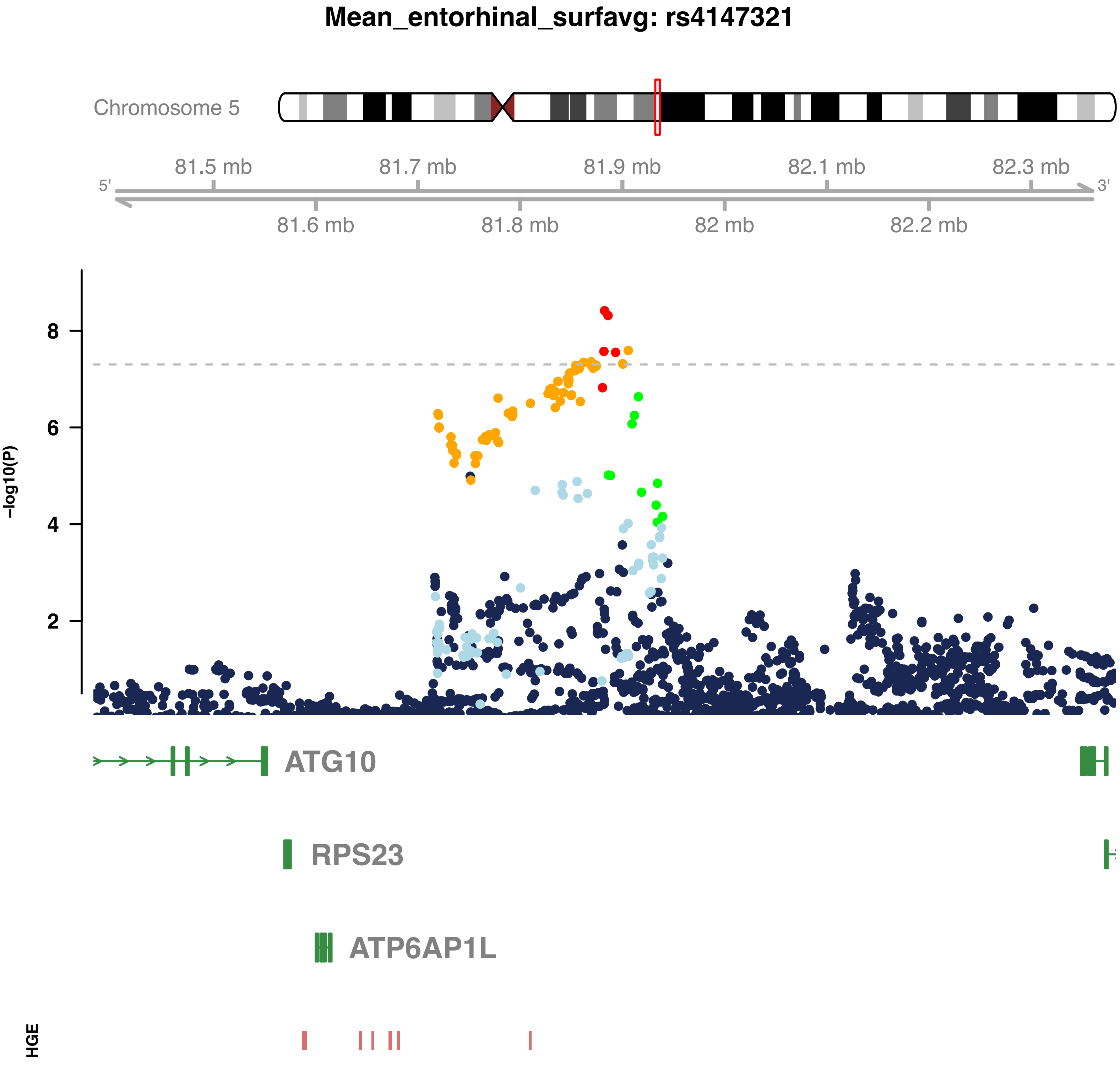

Mean_entorhinal_surfavg: rs4147321
Chromosome 5
81.5 mb
81.7 mb
81.9 mb
82.1 mb
82.3 mb
5'
3'
81.6 mb
81.8 mb
82 mb
82.2 mb
-log10(P)
8
6
4
2
ATG10
RPS23
ATP6AP1L
HGE

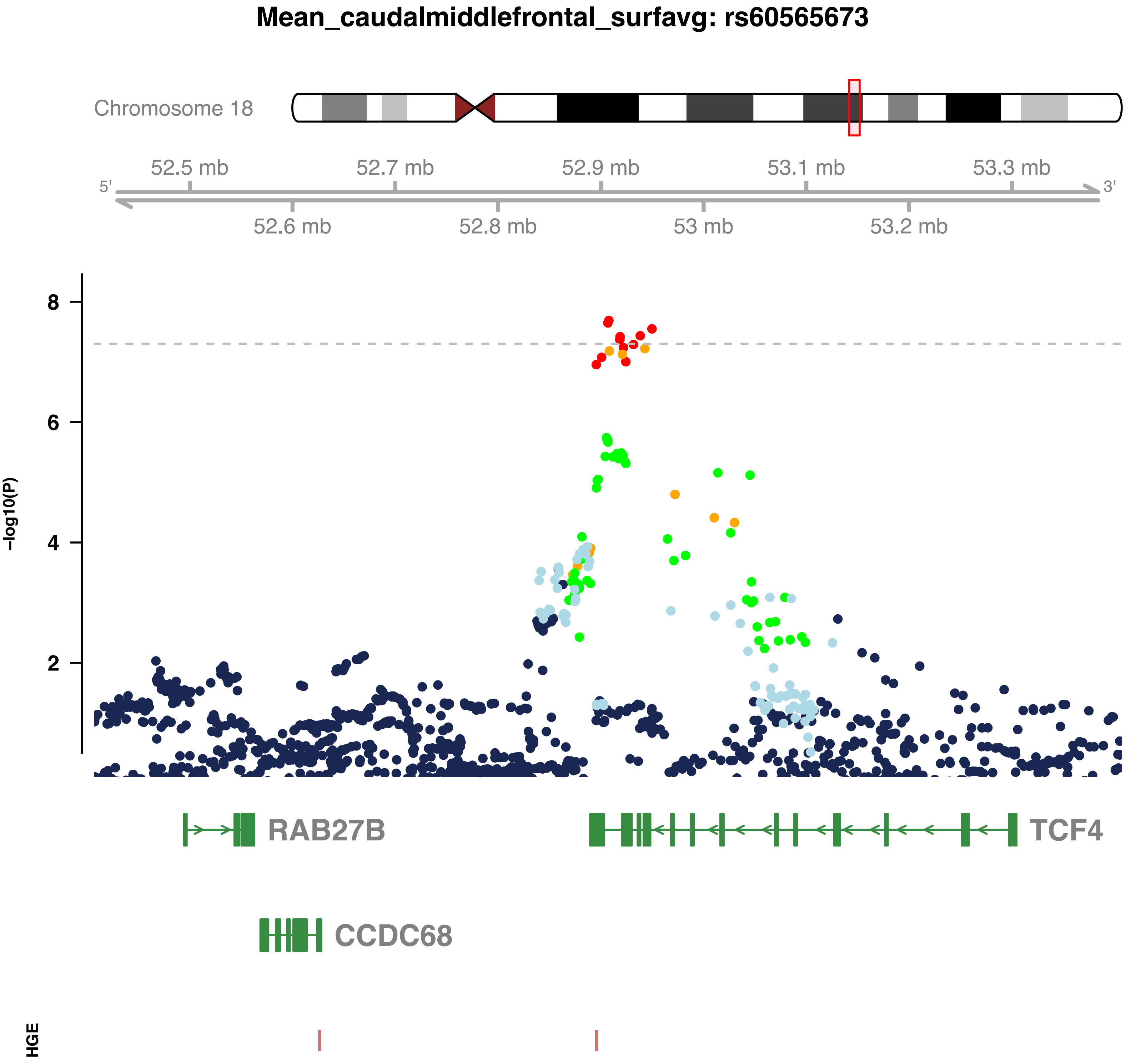
Mean_caudalmiddlefrontal_surfavg: rs60565673
Chromosome 18
52.5 mb
52.7 mb
52.9 mb
53.1 mb
53.3 mb
5'
3'
52.6 mb
52.8 mb
53 mb
53.2 mb
8
6
4
2
-log10(P)
RAB27B
TCF4
CCDC68
HGE

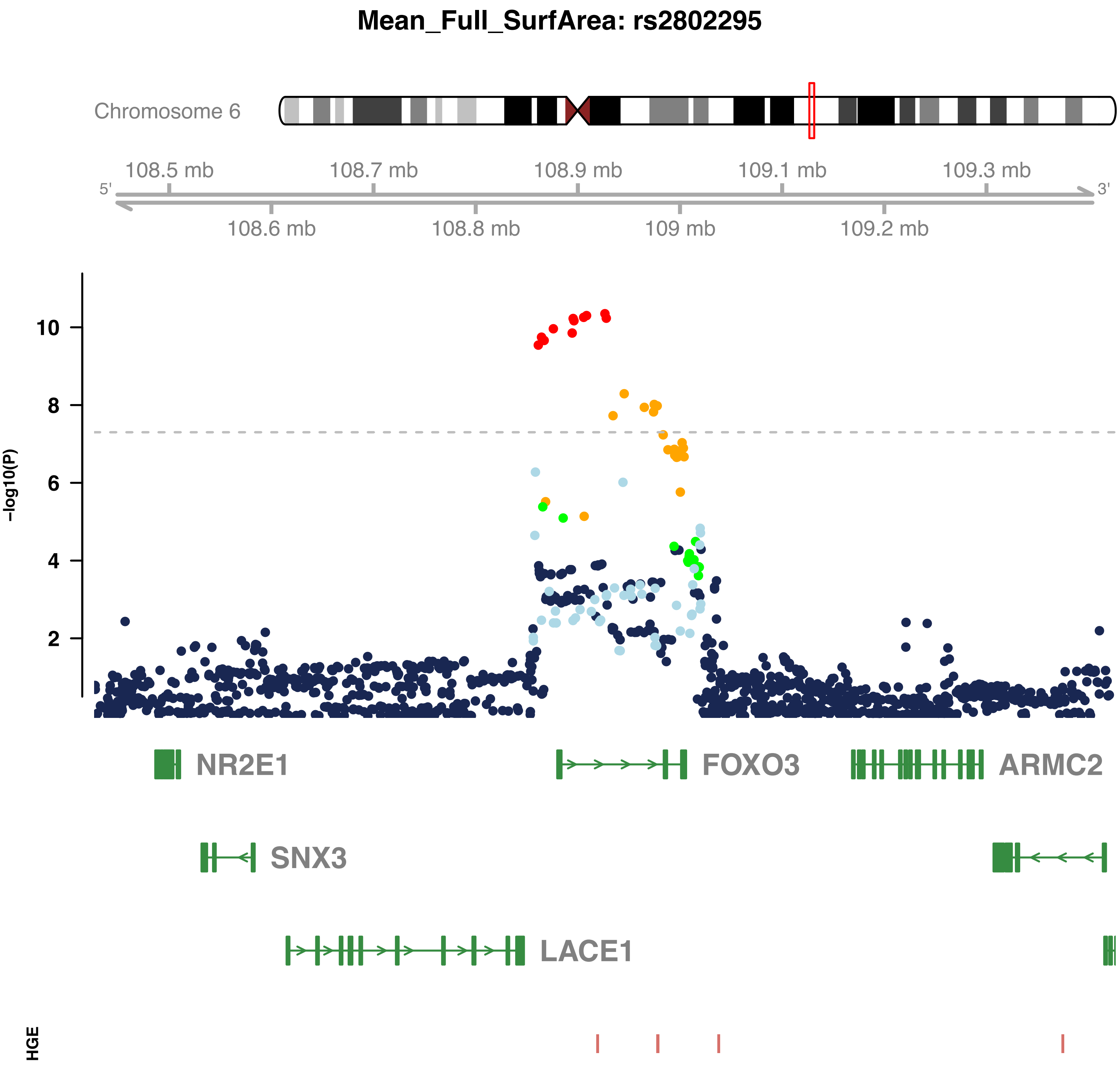

Mean_Full_SurfArea: rs2802295
Chromosome 6
108.5 mb
108.7 mb
108.9 mb
109.1 mb
109.3 mb
5'
3'
108.6 mb
108.8 mb
109 mb
109.2 mb
-log10(P)
10
8
6
4
2
NR2E1
FOXO3
ARMC2
SNX3
LACE1
HGE

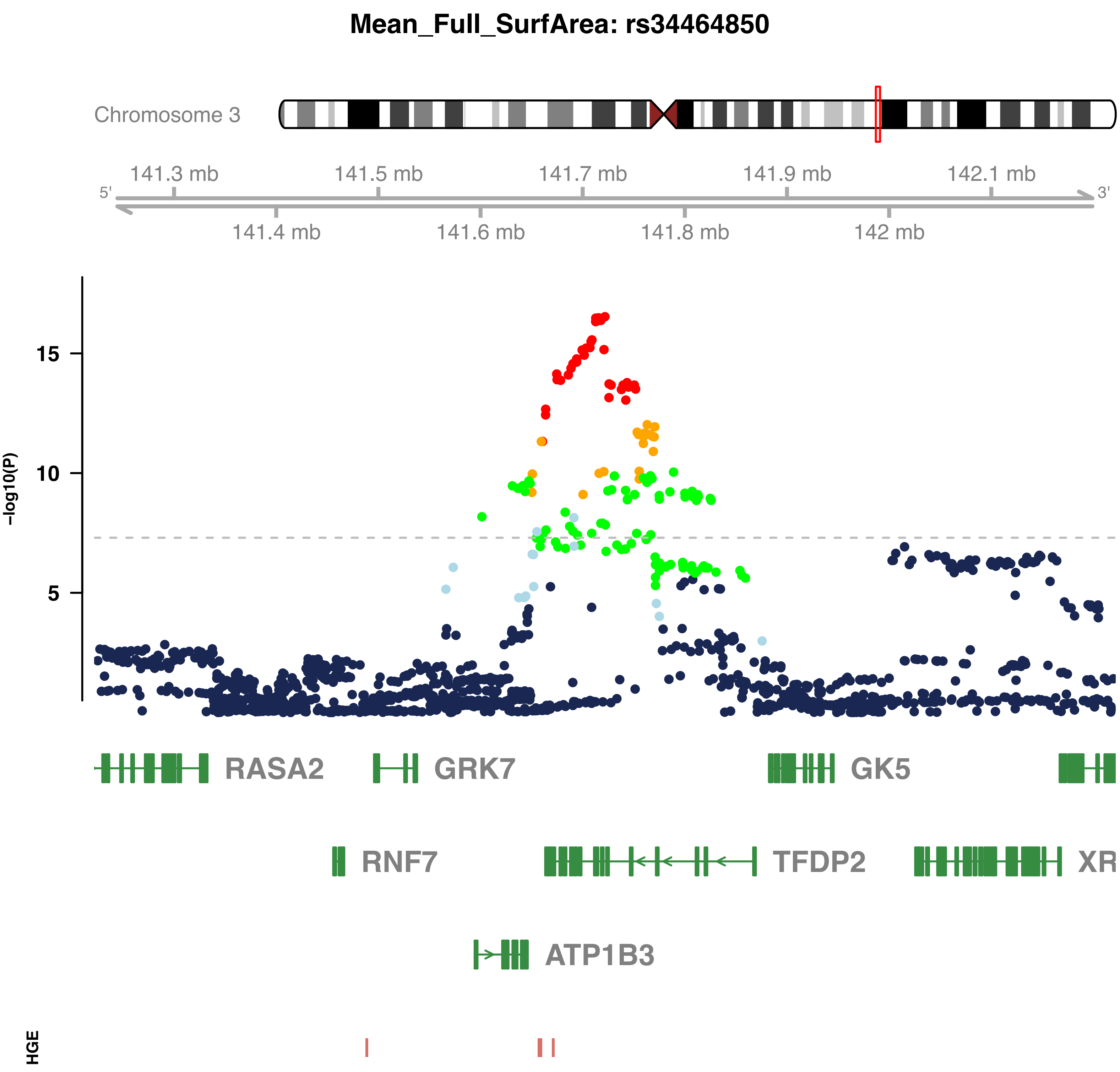

Mean_Full_SurfArea: rs34464850
Chromosome 3
141.3 mb
141.5 mb
141.7 mb
141.9 mb
142.1 mb
141.4 mb
141.6 mb
141.8 mb
142 mb
5'
3'
-log10(P)
15
10
5
RASA2
GRK7
GK5
RNF7
TFDP2
XR
ATP1B3
HGE

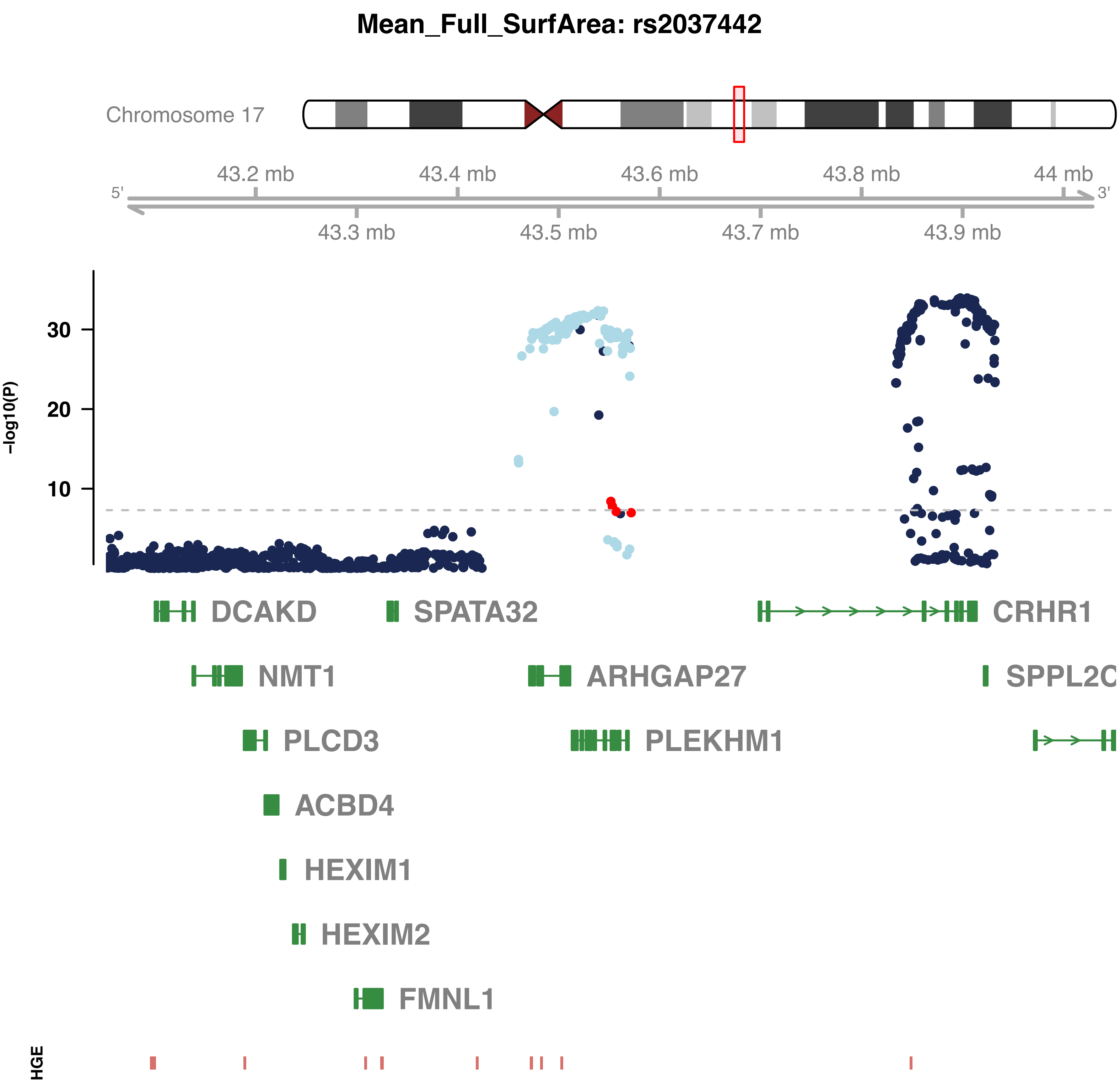

Mean_Full_SurfArea: rs2037442
Chromosome 17
43.2 mb
43.4 mb
43.6 mb
43.8 mb
44 mb
5'
3'
43.3 mb
43.5 mb
43.7 mb
43.9 mb
−log10(P)
30
20
10
DCAKD
SPATA32
CRHR1
NMT1
ARHGAP27
SPPL2C
PLCD3
PLEKHM1
ACBD4
HEXIM1
HEXIM2
FMNL1
HGE

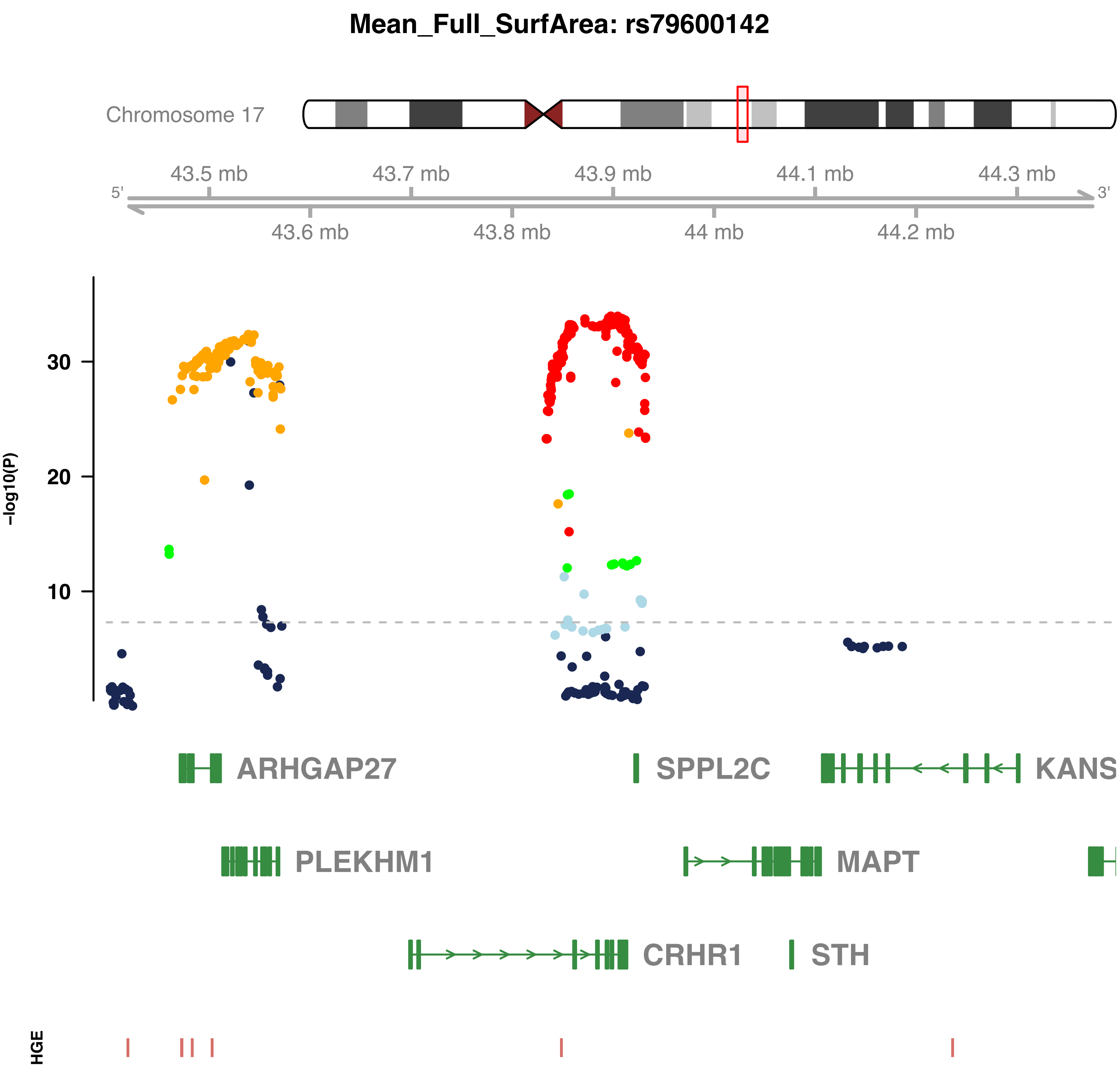

Mean_Full_SurfArea: rs79600142
Chromosome 17
43.5 mb
43.7 mb
43.9 mb
44.1 mb
44.3 mb
5'
3'
43.6 mb
43.8 mb
44 mb
44.2 mb
−log10(P)
30
20
10
ARHGAP27
SPPL2C
KANS
PLEKHM1
MAPT
CRHR1
STH
HGE

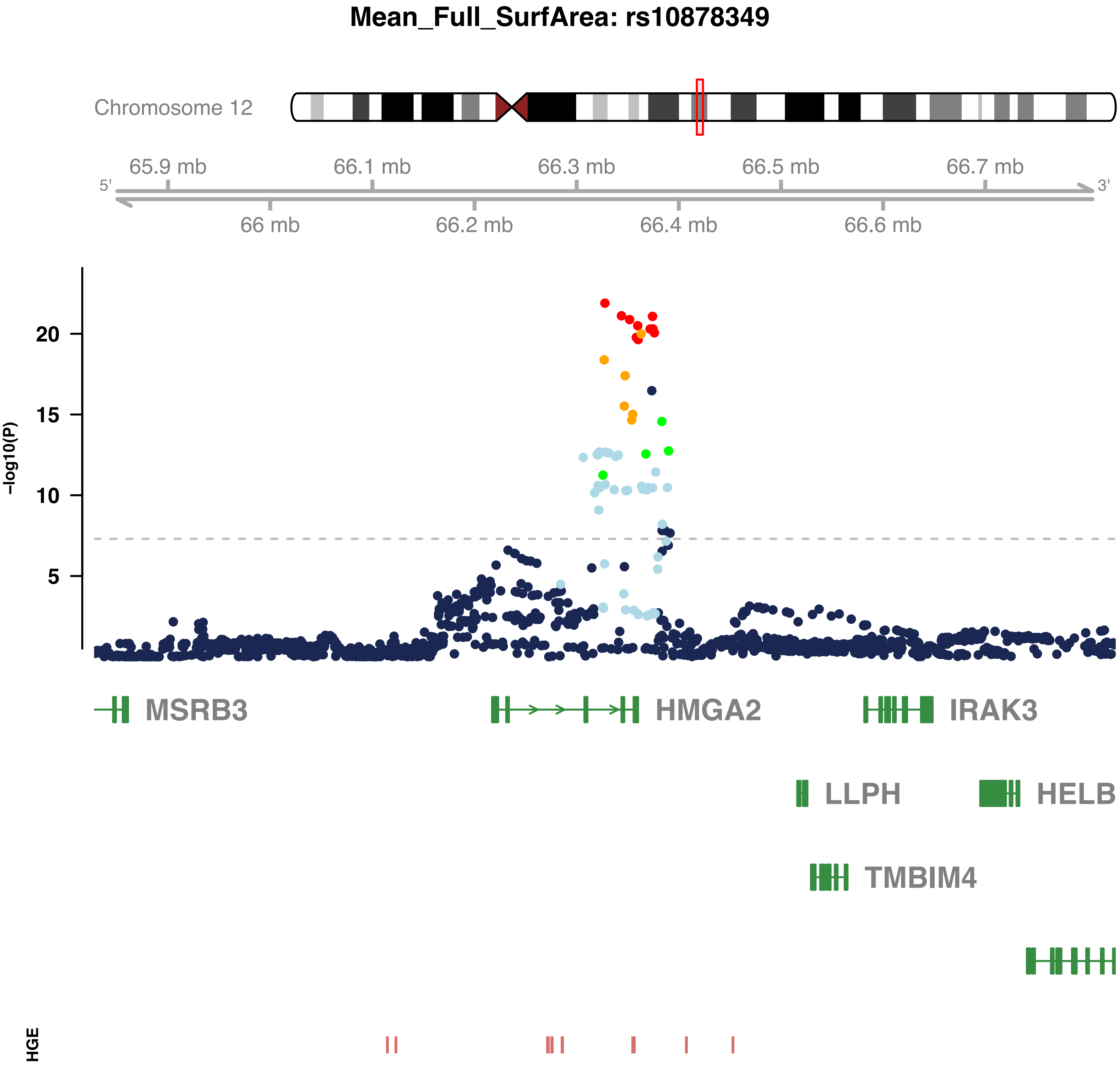
Mean_Full_SurfArea: rs10878349
Chromosome 12
65.9 mb
66.1 mb
66.3 mb
66.5 mb
66.7 mb
5'
3'
66 mb
66.2 mb
66.4 mb
66.6 mb
-log10(P)
20
15
10
5
MSRB3
HMGA2
IRAK3
LLPH
HELB
TMBIM4
HGE

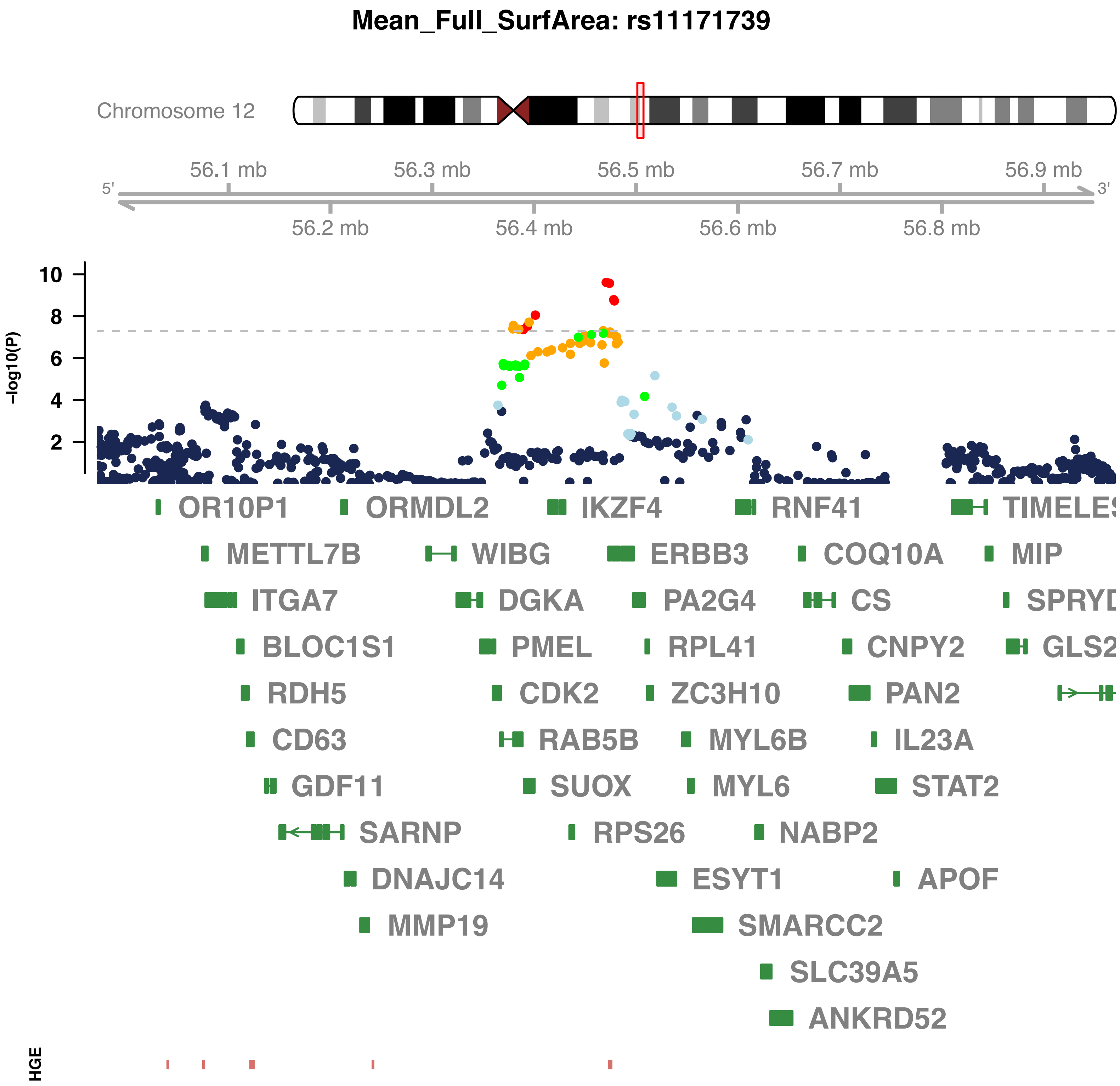
Mean_Full_SurfArea: rs11171739
Chromosome 12
56.1 mb
56.3 mb
56.5 mb
56.7 mb
56.9 mb
5'
3'
56.2 mb
56.4 mb
56.6 mb
56.8 mb
-log10(P)
10
8
6
4
2
OR10P1
ORMDL2
IKZF4
RNF41
TIMELE
METTL7B
WIBG
ERBB3
COQ10A
MIP
ITGA7
DGKA
PA2G4
CS
SPRYD
BLOC1S1
PMEL
RPL41
CNPY2
GLS2
RDH5
CDK2
ZC3H10
PAN2
CD63
RAB5B
MYL6B
IL23A
GDF11
SUOX
MYL6
STAT2
SARNP
RPS26
NABP2
DNAJC14
ESYT1
APOF
MMP19
SMARCC2
SLC39A5
ANKRD52
HGE

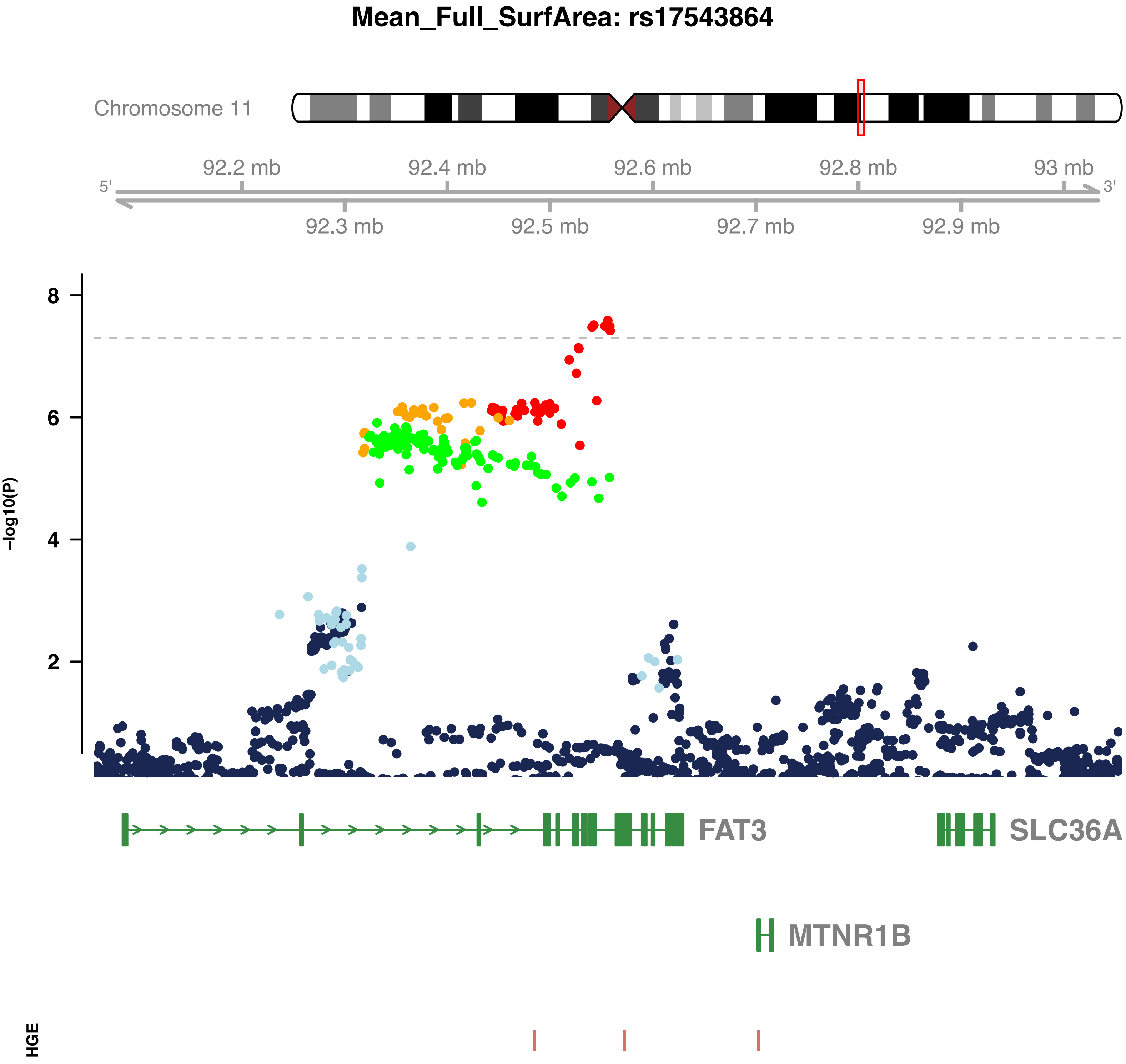

Mean_Full_SurfArea: rs17543864
Chromosome 11
92.2 mb
92.4 mb
92.6 mb
92.8 mb
93 mb
5'
3'
92.3 mb
92.5 mb
92.7 mb
92.9 mb
−log10(P)
8
6
4
2
FAT3
SLC36A
MTNR1B
HGE
